# Microglial Fkbp5 Impairs Post‐Stroke Vascular Integrity and Regeneration by Promoting Yap1‐Mediated Glycolysis and Oxidative Phosphorylation

**DOI:** 10.1002/advs.202512499

**Published:** 2025-12-08

**Authors:** Yanan Li, Yanmei Qiu, Yunlei Yang, Yanhao Wei, Haokun Peng, Longhai Zeng, Pengcheng Li, Rentang Bi, Bo Hu

**Affiliations:** ^1^ Department of Neurology Union Hospital Tongji Medical College Huazhong University of Science and Technology Wuhan 430022 China; ^2^ Department of Medicine Division of Endocrinology Albert Einstein College of Medicine Bronx NY 10461 USA; ^3^ Department of Neuroscience Albert Einstein College of Medicine Bronx NY 10461 USA; ^4^ Einstein‐Mount Sinai Diabetes Research Center Albert Einstein College of Medicine Bronx NY 10461 USA; ^5^ The Fleischer Institute for Diabetes and Metabolism Albert Einstein College of Medicine Bronx NY 10461 USA; ^6^ Department of Ophthalmology Union Hospital Tongji Medical College Huazhong University of Science and Technology Wuhan 430022 China

**Keywords:** BBB, Fkbp5, ischemic stroke, neovascularization, vascular associated microglia

## Abstract

The role of microglia in blood–brain barrier (BBB) leakage and neovascularization after ischemic stroke remains unclear. Here, a post‐stroke perivascular niche of microglia characterized by low expression of M2 markers and elevated glycolysis, oxidative phosphorylation (OXPHOS), and phagocytic activity is identified, which is termed stroke‐activated vascular‐associated microglia (stroke‐VAM). It is found that Fkbp5 acts as a central regulator driving BBB disruption and impaired neovascularization through stroke‐VAM. Single‐nucleus RNA sequencing (snRNA‐seq) analysis of *Cx3cr1* ^Cre^ *Fkbp5* ^flox/flox^ (*Fkbp5* cKO) mice in the ipsilateral hemisphere reveals enhanced interactions between stroke‐VAM and endothelial cells, influencing signaling pathways that maintain BBB integrity and promote neovascularization. After ischemic injury, microglia in *Fkbp5* cKO mice exhibits higher M2 marker expression and reduces glycolysis, OXPHOS, and phagocytosis, resulting in decreased BBB leakage and enhanced angiogenesis. Mechanistically, unbiased snRNA‐seq analysis shows that the Hippo signaling pathway is altered in *Fkbp5* cKO stroke‐VAM. Fkbp5 inhibits Yap1 phosphorylation, facilitating its nuclear translocation. These findings provide new insights into how the perivascular microglial niche contributes to both the degradation and regeneration of cerebral vasculature, offering potential therapeutic avenues for acute ischemic stroke.

## Graphical abstract

A post‐stroke perivascular niche of microglia characterized by low expression of M2 markers and elevated glycolysis, oxidative phosphorylation (OXPHOS), and phagocytic activity is identified, which is termed stroke‐activated vascular‐associated microglia (stroke‐VAM). Fkbp5 acts as a central regulator driving BBB disruption and impaired neovascularization through stroke‐VAM.

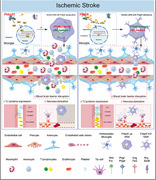

## 1 Introduction

Acute ischemic stroke (AIS) is a leading cause of mortality and adult disability worldwide, with current clinical interventions limited to thrombolysis and mechanical thrombectomy. These therapies benefit only 5–10% of patients with AIS due to a narrow therapeutic time window.^[^ ^1^ ^]^ A sudden cessation of cerebral blood flow impairs ionic balance and induces massive cell death minutes after stroke. This is followed by striking blood–brain barrier (BBB) disruption and overwhelming immune cell extravasation, resulting in vasogenic edema and inflammation in the subacute phase of AIS (1—3 days post‐ishcemia).^[^ ^2^ ^]^ Loss of BBB integrity, which is tightly regulated by immune cells, is characterized by reduced of tight junctions (TJs) and loss of the endothelial cell (EC) syncytium. This BBB integrity loss plays a detrimental role in AIS by facilitating an enormous influx of water, blood‐borne components, and peripheral immune cells into the cerebral parenchyma.^[^ ^3^, ^4^ ^]^ Moreover, post‐ischemic cerebrovascular regeneration and functional BBB restoration are critical steps that enable adequate oxygen and nutrient supply for tissue regeneration and functional recovery in the chronic phase of AIS.^[^ ^5^ ^]^ Angiogenesis begins with the proliferation and migration of EC, which occurs as early as 12–24 h post‐stroke. This is followed by lumen formation, extracellular matrix reconstruction, and BBB tight junction establishment. This stage persists for up to 21 days after AIS onset.^[^ ^6^ ^]^ Close cell–cell interactions and ligand–receptor communications between immune cells and EC significantly impact vascular formation and maturation.^[^ ^7^ ^]^ Hence, understanding the inflammatory mechanisms governing the loss of BBB integrity and cerebrovasculature re‐establishment is of particular research interest as this could identify valuable therapeutic opportunities.

Microglia are brain‐resident innate immune cells that constantly survey the cerebral parenchyma. They have a small cell body with thin, ramified processes.^[^ ^8^ ^]^ Microglia are activated within several minutes of stroke onset, transforming into amoeboid‐like cells with enlarged cell bodies and thick processes.^[^ ^9^ ^]^ Extensive research on microglia has yielded conflicting reports on their contribution to BBB integrity and neovascularization. Some studies in central nervous system disorders, including stroke and multiple sclerosis, demonstrated that microglia accelerate BBB degradation by directly engulfing ECs and downregulating TJ proteins in ECs through pro‐inflammatory factors and reactive oxygen species (ROS).^[^ ^10^, ^11^ ^]^ However, recent studies showed that postnatal deletion of microglia did not influence BBB integrity.^[^ ^12^ ^]^ Haruwaka et al. discovered that microglia migrate, physically contact EC, and express TJ proteins to protect BBB integrity under systemic inflammation.^[^ ^13^ ^]^ Moreover, multiple lines of evidence highlight the pro‐angiogenic role of microglia in the retinal angiogenic niche and glioma.^[^ ^11^, ^14^ ^]^ Consistent with the findings in retinopathy, microglia could promote angiogenesis and cerebrovascular re‐establishment after stroke by producing angiogenic molecules (TGF‐β, IL‐6, and VEGF‐A).^[^ ^15^, ^16^ ^]^ However, other studies reported that M1 microglia polarization caused by ischemia/reperfusion (I/R) injury inhibits the angiogenic activity of ECs through the AMPK signaling pathway.^[^ ^17^ ^]^ These divergent observations could potentially be attributed to the tremendous heterogeneity in microglial reprogramming subtypes in response to ischemic stroke. The traditional M1/M2 dichotomy is too simplistic to comprehensively delineate how microglia orchestrate the loss of BBB integrity and neovascularization after ischemic stroke. Therefore, investigating microglial multipotential reprogramming at a single‐cell resolution is imperative.

Herein, we discovered a post‐stroke perivascular microglia niche, characterized by low expression of M2 markers and elevated glycolysis, OXPHOS, and phagocytotic activity. The cells in this niche were named stroke‐activated vascular‐associated microglia (stroke‐VAM). Furthermore, we identified Fkbp5 as a key regulator influencing BBB disruption and impaired neovascularization associated with stroke‐VAM. Using transgenic mice, single‐nucleus RNA sequencing (snRNA‐seq), flow cytometry, and Seahorse metabolic assays, we demonstrated that abolition of Fkbp5‐mediated stroke‐VAM significantly promoted BBB integrity and angiogenesis following ischemic stroke. Our results provide new perspectives on the contribution of the perivascular microglia niche to the degradation and regeneration of the cerebral vasculatures, potentially paving the way for therapies for AIS.

## 2 Results

### 2.1 Identification of Stroke‐VAM in Ischemic Brain

**Figure 1:**
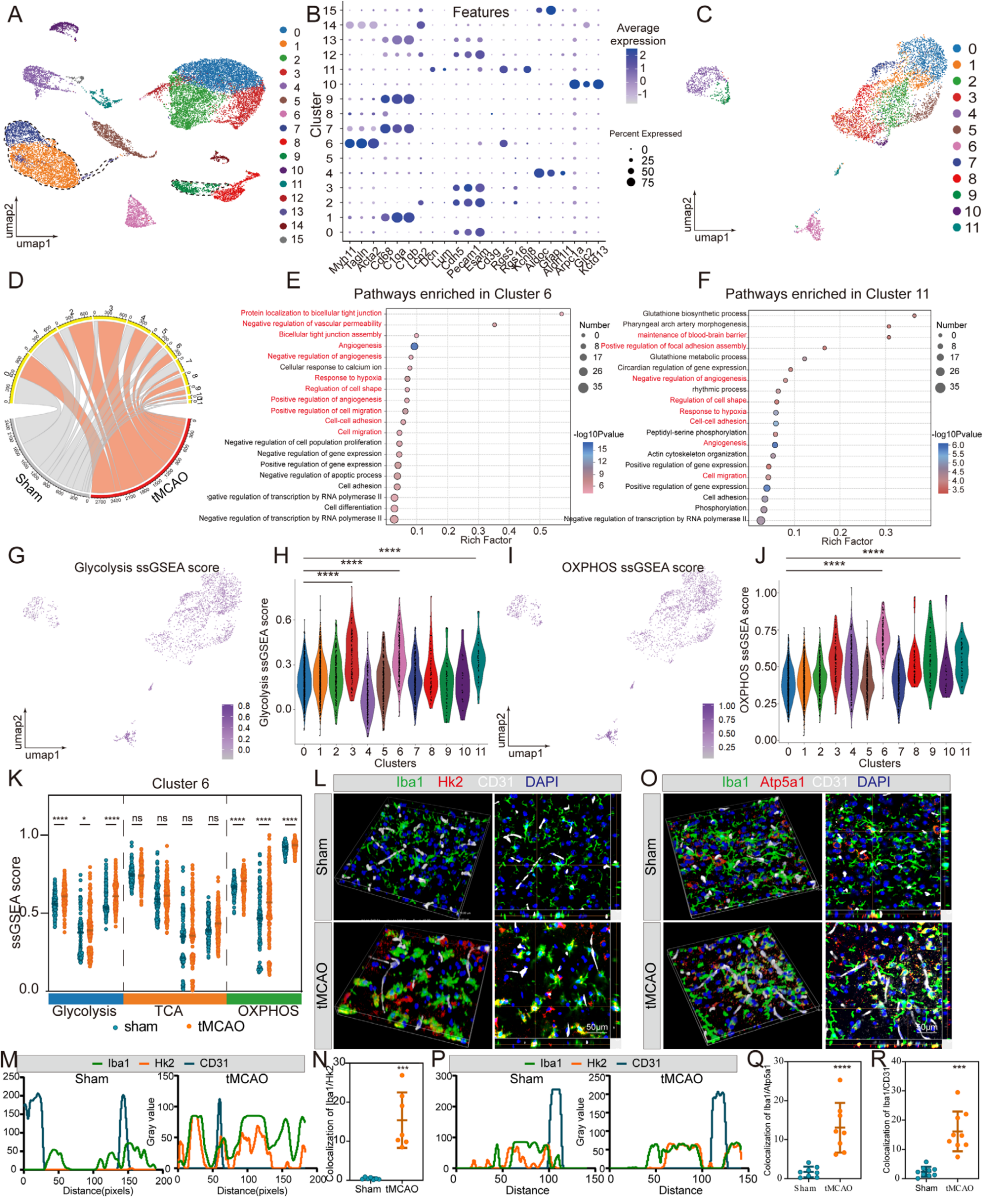
Identification of stroke‐activated vascular associated microglia (stroke‐VAM) in ischemic brain. A) UMAP plot visualizing and unsupervised clustering of all cells colored by cell types. B) Dot plot representing the expression level of cell type specific genes within each cluster. C) UMAP plot showing the unsupervised clustering of microglia colored by subclusters. D) Bar plot of the proportion of microglia subcluster in each group. E) GO‐BP terms enriched for microglial subcluster 6 under the threshold of *P*‐value < 0.05. F) GO‐BP terms enriched for microglial subcluster 11 under the threshold of *P*‐value < 0.05. G) UMAP plot visualizing the glycolysis ssGSEA score distribution in each microglia subclusters. H) Violin diagram representing the glycolysis ssGSEA score level in each microglia subclusters. I) UMAP plot visualizing the OXPHOS ssGSEA score distribution in each microglia subclusters. J) Violin diagram representing the OXPHOS ssGSEA score level in each microglia subclusters. K) Scatter plot displaying ssGSEA score level of glycolysis, TCA, OXPHOS in microglia subcluster 6 between sham group and tMCAO group. L–N) Representative micrographs (L), colocalization pattern (M) and colocalization quantitative data (N) of brain sections immunostaining Iba1 (green), Hk2 (red), CD31 (white) in peri‐infarct area of tMCAO mice (*n* = 7) and the corresponding area of sham mice (*n* = 7). Nuclei were stained with DAPI (blue). O–Q) Representative micrographs (O), colocalization pattern (P) and colocalization quantitative data (Q) of brain sections immunostaining Iba1 (green), Atp5a1 (red), CD31 (white) in peri‐infarct area of tMCAO mice (*n* = 8) and the corresponding area of sham mice (*n* = 8). R) Colocalization quantitative data of Iba1 and CD31 in peri‐infarct area of tMCAO mice (*n* = 9) and the corresponding area of sham mice (*n* = 9). Data are presented as mean ± SD; unpaired *t*‐test; one‐way ANOVA; ns, not significant; * *P* < 0.05; ** *P* < 0.01; *** *P* < 0.001; **** *P* < 0.0001. UMAP, Uniform Manifold Approximation and Projection; GO‐BP, Gene Ontology: Biological Process; ssGSEA, single‐sample Gene Set Enrichment Analysis; TCA, Tricarboxylic Acid Cycle; OXPHOS, Oxidative Phosphorylation; tMCAO, transient Middle Cerebral Artery Occlusion; DAPI, 4′,6‐diamidino‐2‐phenylindole.

To comprehensively characterize microglial heterogeneity in the ischemic brain, we analyzed publicly available single‐cell RNA sequencing (scRNA‐seq) data (GSE 174574) obtained from the ipsilateral hemisphere of transient middle cerebral artery occlusion (tMCAO) and sham mice at 1 day post‐stroke. After quality control and dimensionality reduction using UMAP, 16 cell clusters were annotated based on canonical cell‐type markers (**Figure** **1**A,B). These included six EC clusters (clusters 0, 2, 3, 5, 12, and 15), four microglial clusters (clusters 1, 7, 9, and 13), one astrocyte cluster (cluster 4), two pericyte clusters (clusters 6 and 11), one tissue‐resident macrophage cluster (cluster 8), one oligodendrocyte cluster (cluster 10), and one neutrophil cluster (cluster 14). Microglia were identified by canonical markers such as *C1qa, C1ab, C1qc, Aif1, Cx3cr1*, and *Tmem119* (Figure S1A–F, Supporting Information). Microglial cells were then extracted and re‐clustered into 12 subclusters (Figure 1C). The proportions of microglial clusters 1, 7, and 11 were markedly decreased, whereas clusters 2, 3, 8, 9, and 10 were significantly increased at 1 day post‐tMCAO compared with the sham group (Figure 1D). Subcluster‐specific genes were identified through differential expression analysis. Clusters 0, 1, and 5 expressed high levels of homeostatic genes (*P2ry12*, *Cx3cr1*, and *Tmem119*) (Figure S2A–C, Supporting Information). Given that M1, M2, and disease‐associated microglia (DAM) are well‐characterized microglial phenotypes.^[^ ^18^, ^19^ ^]^ We calculated single‐sample gene set enrichment analysis (ssGSEA) scores for M1‐, M2, and DAM‐specific expressional profiles across the 12 clusters (Figure S2D–I, Supporting Information). Clusters 3, 8, and 10 displayed high DAM ssGSEA scores (Figure S2D,E, Supporting Information) and elevated expression of DAM signature genes (*Spp1*, *Cd63*, and *Cd9*) (Figure S2J–L, Supporting Information). Clusters 2 and 10 exhibited significantly higher M1 ssGSEA scores (Figure S2F,G, Supporting Information) and increased expression of M1 markers (*Tnf* and *Cxcl10*) (Figure S2M,N, Supporting Information). In contrast, M2 ssGSEA scores and the M2‐specific gene *Mrc1* were uniquely elevated in clusters 4 and 9 (Figure S2H,I,O, Supporting Information). To further investigate the remaining undefined microglial clusters (clusters 6, 7, and 11), we performed gene ontology (GO) enrichment analysis of their cluster‐specific differential genes. Cluster 7 represented a proliferative state of microglia, as its marker genes were enriched for DNA replication processes (Figure S3A, Supporting Information). Interestingly, two microglial clusters showed strong associations with cerebrovascular functions. Cluster 6 displayed enrichment in pathways related to protein localization to bicellular tight junctions, negative regulation of vascular permeability, and bicellular tight junction assembly (Figure 1E). Cluster 11 was associated with artery morphogenesis and maintenance of the BBB (Figure 1F). The pseudotime analysis revealed that cluster 6 and cluster 11 were well‐distinguished functional subsets by distributing at the same distinctive positions of the pseudotime trajectory (Figure S3B,C, Supporting Information). Notably, both clusters 6 and 11 expressed several endothelial‐related markers, including *Flt1*, *Slc2a1*, *Tjp1*, *Cldn5*, and *Cdh5*. Based on these features, we hypothesized that these clusters represent perivascular microglia, which may engulf ECs following I/R injury, as previously reported.^[^ ^10^ ^]^

Given the high heterogeneity of microglia and their critical role in BBB disruption after ischemic stroke, we next focused on delineating the unique features of cluster 6 and cluster 11. As described above, these two populations did not display M1‐, M2‐, or DAM‐like characteristics. Notably, both clusters exhibited extremely low M2 ssGSEA scores, ranking 12th and 9th, respectively (Figure S2I, Supporting Information). Further analysis revealed that clusters 6 and 11 showed increased energetic dependence on both glycolysis and OXPHOS compared with other clusters, as confirmed by the expression of canonical metabolic pathway–associated genes (Figure 1G–J; Figure S3D,E, Supporting Information). To determine whether I/R injury altered their metabolic activity, we compared glycolytic and OXPHOS scores between the tMCAO and sham groups. In the tMCAO group, both glycolytic and OXPHOS activities were markedly elevated in cluster 6 (Figure 1K), with a similar upregulation observed in cluster 11 (Figure S3F, Supporting Information). These results indicate that VAM clusters are hyperglycolytic and hyperOXPHOS, distinct from previously reported microglial metabolic profiles.^[^ ^20^ ^]^ To validate the presence of this unique metabolic state in perivascular microglia, we performed immunofluorescence (IF) staining for key enzymes involved in glycolysis (Hk2) and OXPHOS (Atp5a1) in perivascular microglia at 1 day post‐tMCAO (Figure 1L–R). A large number of activated microglia were observed surrounding the cerebrovasculature in the tMCAO group (Figure 1R). These perivascular microglia showed significantly increased expression of Hk2 and Atp5a1, as indicated by colocalized staining of Hk2/Atp5a1 and Iba1 in proximity to CD31 (Figure 1L–Q). Because mitochondrial integrity is crucial for OXPHOS,^[^ ^21^ ^]^ we further examined mitochondrial activity. Cluster 6 exhibited significantly higher ssGSEA scores for apoptotic mitochondrial changes, mitochondrial fragmentation, positive regulation of mitochondrial depolarization, and regulation of mitochondrial depolarization compared with other clusters (Figure S3G–N, Supporting Information). Moreover, endocytosis and phagocytosis activities were elevated in cluster 6 (Figure S3P–Q, Supporting Information), suggesting that perivascular microglia may engulf ECs after I/R injury.

Collectively, these findings define the distinct characteristics of VAM in ischemic stroke: low M2 marker expression, enhanced glycolysis and OXPHOS, high phagocytic activity, and mitochondrial fragmentation. Given their unique transcriptomic and metabolic profiles, distinct from previously described microglia states, we designated this population as “stroke‐activated vascular‐associated microglia” (stroke‐VAM), which will be referred to henceforth. The stringent set of markers for stroke‐VAM was provided in Table S1 (Supporting Information). We further externally validated the existence of stroke‐VAM in another independent dataset (GSE233812) by utilizing these identified stroke‐VAM markers. After quality control and dimensionality reduction by UMAP (Figure S3R, Supporting Information), 16 cell clusters were annotated. According to microglia markers (*Aif1, C1qc, C1qb, P2ry12*), cluster 3 was identified as microglia (Figure S3S, Supporting Information). Microglia were further dimensionality reduced to 7 clusters. Among these clusters, cluster 5 displayed a significant higher stroke‐VAM ssGSEA score (Figure S3T, Supporting Information), indicating the identify of stroke‐VAM across datasets.

### 2.2 Screening Key Regulator of Stroke‐VAM: Fkbp5 is Upregulated in Microglia and Related to Stroke‐VAM after Ischemic Stroke

**Figure 2:**
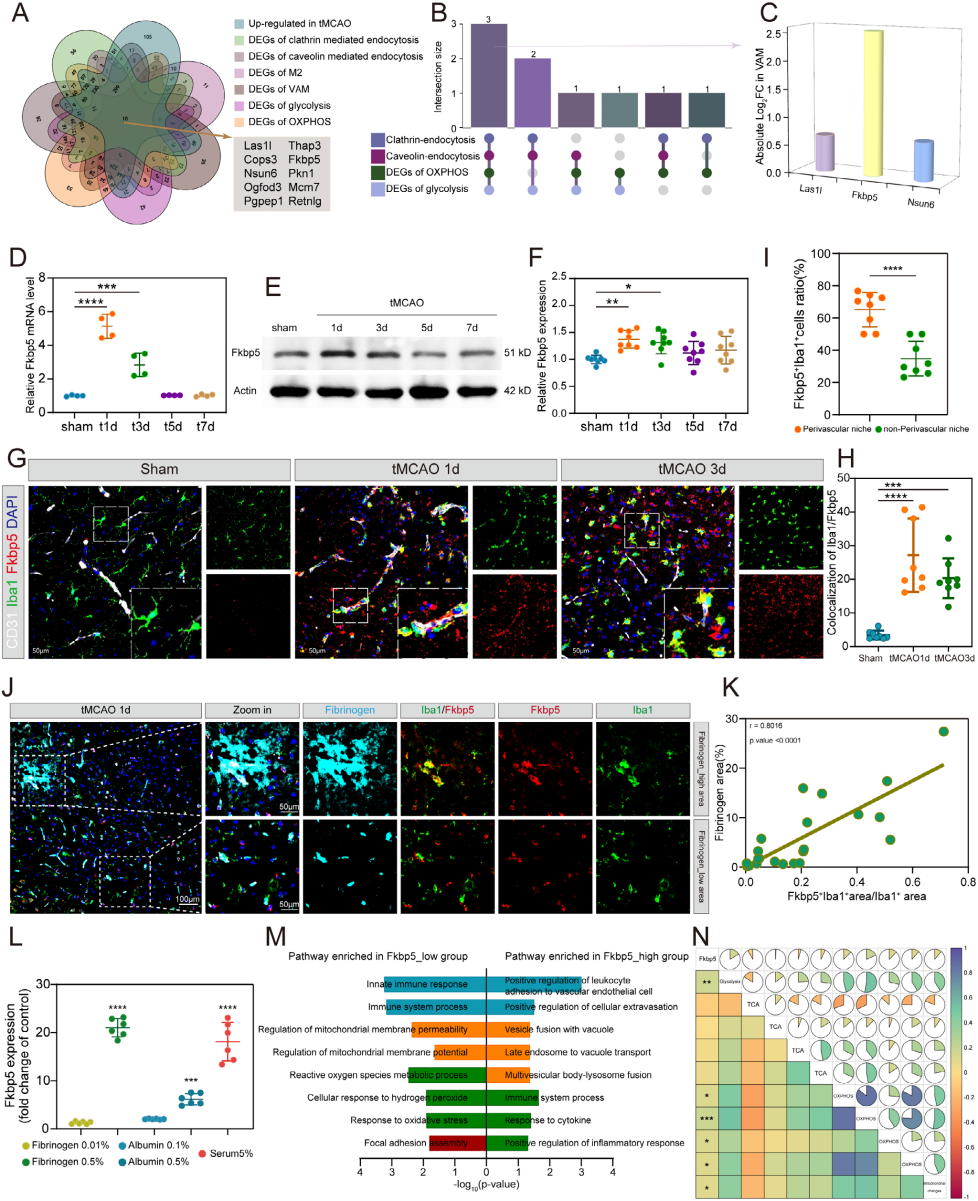
Screening key regulator of stroke‐VAM: Fkbp5 is upregulated in microglia and related to stroke‐VAM after ischemic stroke. A) Venn diagram displaying 10 overlapping genes among 7 stroke‐VAM specific characteristics DEGs. B) Histogram showing 3 overlapping genes (*Fkbp5*, *Las1l*, *Nsun6*) among top 8 DEGs from A) involved in endocytosis, OXPHOS, and glycolysis. C) Histogram representing the absolute log2FC in stroke‐VAM among the 3 overlapping genes from (B). D) qPCR exploring the mRNA level of Fkbp5 in ischemic cortex from sham, 1 day after tMCAO, 3 days after tMCAO, 5 days after tMCAO, and 7 days after tMCAO. *n* = 4 for each group. E,F) Representative immunoblots (E) and quantitative data (F) of Fkbp5 protein expression in ischemic cortex from sham, 1 day after tMCAO, 3 days after tMCAO, 5 days after tMCAO, and 7 days after tMCAO. *n* = 8 for each group. G,H) Representative pictures (I) and quantitative data (J) of immunofluorescent Fkbp5 and Iba1 in peri‐infarct region from sham, 1‐day post‐stroke, and 3 days post‐stroke. *n* = 8 for each group. I) Quantitative data of immunofluorescent Fkbp5, Iba1, and Cd31 showing the expression of microglial Fkbp5 in perivascular niche versus non‐perivascular area in peri‐infarct region at 1‐day post‐stroke. *n* = 8 for each group. J) Representative pictures of immunofluorescent Fkbp5, Fibrinogen, and Iba1 showing the expression of microglial Fkbp5 in Fibrinogen high area versus Fibrinogen low area at 1‐day post‐stroke. K) Analysis graph of the correlation between microglial Fkbp5 expression levels and fibrinogen leakage levels at 1 day post‐stroke. *n* = 23. L) QPCR analysis of Fkbp5 mRNA level in BV2 cells treated with different concentration of fibrinogen, albumin, and serum. Results were normalized to Fkbp5 expression of unstimulated microglia. *n* = 6 per group. M) GO‐BP terms enriched for Fkbp5 high expression group and Fkbp5 low expression group under the threshold of *P*‐value < 0.05. N) Correlation heat map displaying the correlation between Fkbp5 and glycolysis, TCA, or OXPHOS according to Fkbp5 expression and metabolic ssGSAE score. Data are presented as mean ± SD. unpaired *t*‐test; one‐way ANOVA; Pearson correlation coefficient; * *P* < 0.05; ** *P* < 0.01; *** *P* < 0.001; **** *P* < 0.0001. DEGs, Differentially Expressed Genes GO‐BP, Gene Ontology: Biological Process; ssGSEA, single‐sample Gene Set Enrichment Analysis; TCA, Tricarboxylic Acid Cycle; OXPHOS, Oxidative Phosphorylation; tMCAO, transient Middle Cerebral Artery Occlusion; OGD/R, Oxygen Glucose Deprivation/Reperfusion; DAPI, 4′,6‐diamidino‐2‐phenylindole.

To identify potential regulators of stroke‐VAM, we performed a multi‐criteria differential gene expression analysis incorporating i) genes upregulated in tMCAO; ii) DEGs associated with clathrin‐mediated and caveolin‐mediated endocytosis; iii) DEGs related to M2 and stroke‐VAM phenotypes; and iv) DEGs linked to glycolysis and OXPHOS, comparing high and low ssGSEA clusters (**Figure** **2**A). Ten DEGs met these criteria, of which the top eight involved in endocytosis, OXPHOS, and glycolysis were selected for further analysis. Three overlapping genes, *Las1l*, *Fkbp5*, and *Nsun6*, were identified (Figure 2B). Among them, Fkbp5 exhibited the most significant change, with an absolute Log_2_FC being 2.56 in stroke‐VAM (Figure 2C). To validate this finding, we examined Fkbp5 expression in the ipsilateral cerebral cortex at 1, 3, 5, and 7 days after tMCAO. The mRNA level of Fkbp5 peaked at day 1 post‐stroke and gradually declined to baseline by day 5 (Figure 2D). Consistent with this, protein levels were significantly elevated at days 1 and 3 (Figure 2E,F), suggesting that Fkbp5 functions primarily during the acute and subacute phases of AIS. We next assessed the cellular localization of Fkbp5. IF staining of the ipsilateral cortex showed colocalization of Fkbp5 with microglial marker (Iba1) and neuronal marker (NeuNn), but not with endothelial CD31 or astrocytic (GFAP) markers (Figure S4A,B, Supporting Information). UMAP and violin plots further confirmed high Fkbp5 expression in microglial clusters (Figure S4C,D, Supporting Information). Analysis of an independent dataset (GSE225948) corroborated the microglial‐specific expression of Fkbp5 (Figure S4E, Supporting Information). The slight discrepancy between in vivo and sequencing data likely reflects neuron underrepresentation in single‐cell datasets. Additionally, scRNA‐seq analysis demonstrated that Fkbp5 expression is enriched in stroke‐VAM and upregulated after tMCAO (Figure S4F–H, Supporting Information). In an in vivo OGD/R model, Fkbp5 protein levels were statistically increased in BV2 cells (Figure S4I,J, Supporting Information), and fluorescence intensity analysis confirmed this upregulation (Figure S4K,L, Supporting Information). IF staining of Fkbp5, Iba1, and CD31 at days 1 and 3 post‐tMCAO confirmed that perivascular microglia exhibit strong Fkbp5 expression, peaking at day 1 (Figure 2G,H), consistent with qRT‐PCR and western blotting (WB) results. To assess the uniqueness of Fkbp5 in stroke‐VAM, we quantified Fkbp5 levels in microglia physically contacting blood vessels versus those without such contact. The number of stroke‐VAM in Fkbp5‐positive microglia was nearly double that in non‐perivascular microglia (Figure 2I; Figure S4M, Supporting Information). Next, we assessed the relationship between microglial Fkbp5 expression levels and vascular leakage. Microglial Fkbp5 levels showed a significant positive correlation with fibrinogen leakage levels (Figure 2J,K). These findings suggest that Fkbp5 is specifically elevated in stroke‐VAM and positively correlates with the degree of vascular leakage. Given the perivascular location of stroke‐VAMs, we further investigated whether Fkbp5 upregulation in stroke‐VAMs could be induced by extravasated blood‐derived macromolecules. We treated BV2 cells with varying concentrations of fibrinogen, albumin, and serum for 12 h and measured Fkbp5 mRNA levels. Fkbp5 levels were strongly induced by fibrinogen, albumin, and serum in a dose‐dependent manner (Figure 2L). To explore the functional association between Fkbp5 and stroke‐VAM, we performed GO enrichment of DEGs stratified by Fkbp5 expression in scRNA‐seq data. DEGs linked to vascular permeability, phagocytosis, and mitochondrial function were significantly enriched among Fkbp5‐associated genes (Figure 2M). Pathways enriched in the Fkbp5_low group included innate immune response, regulation of mitochondrial membrane potential, ROS metabolism, and focal adhesion assembly, whereas the Fkbp5high group was enriched for leukocyte adhesion to endothelium, vesicle fusion, and immune system processes (Figure 2M). Besides, we found Fkbp5 expression is positively correlated with glycolysis score and OXPHOS score, but not with TCA score (Figure 2N), which is an important metabolic pattern of stroke‐VAM. Therefore, we screened a critical regulator (Fkbp5) of stroke‐VAM and figured out the spatial and temporal characteristics of Fkbp5 in the ischemic hemisphere. Moreover, the function of Fkbp5 mostly matches the transcriptome signature of stroke‐VAM.

To explore whether the level of Fkbp5 in blood related to the severity of stroke, we measured serum FKBP5 concentrations in patients with acute ischemic stroke. A total of 92 matched pairs (184 patients) with acute ischemic stroke were included in this study. Patients were categorized into a poor outcome group (mRS 3–6, *n* = 92) and a good outcome group (mRS 0–2, *n *= 92) based on 90‐day functional outcomes. The baseline characteristics of the participants are summarized in Table S2 (Supporting Information). The median baseline serum FKBP5 concentration was significantly higher in the poor outcome group (0.346 ng mL^−1^) compared to the good outcome group (0.228 ng mL^−1^, *P* = 0.042) (Figure S5A, Supporting Information). Furthermore, a significant positive correlation was observed between serum FKBP5 concentration and the baseline NIHSS score (*r* = 0.426, *P *< 0.001) (Figure S5B, Supporting Information), indicating an association between higher FKBP5 levels and more severe initial neurological impairment. In summary, higher serum FKBP5 concentration is associated with more severe neurological deficits at admission and poorer functional outcome at 90 days in patients with acute ischemic stroke.

### 2.3 Inhibiting Stroke‐VAM by Fkbp5 Deletion in Microglia Attenuates BBB Disruption and Inflammation Post‐Stroke

To elucidate the role of Fkbp5‐mediated stroke‐VAM in ischemic stroke, we generated conditional microglia‐specific *Fkbp5* knockout (*Fkbp5* ^ΔMG^) mice using the Cre–loxP system (Figure S6A, Supporting Information). Floxed mice were constructed by inserting loxP sites flanking exon 3 of the *Fkbp5* gene (Figure S56B, Supporting Information), and Cre recombinase was placed downstream of the *Cx3cr1* promoter to ensure microglial specificity (Figure S6B, Supporting Information). Crossing *Fkbp5* ^fl^/^fl^ mice with *Cx3cr1*‐Cre mice successfully produced the microglia‐specific *Fkbp5* knockout line (Figure S6C, Supporting Information). IF staining confirmed the selective deletion of Fkbp5 protein in microglia (Iba1^+^ cells), with no observable change in Fkbp5‐Neun colocalization (Figure S6D, Supporting Information). WB analysis further validated the marked reduction of Fkbp5 protein levels in Fkbp5^ΔMG^ mice (Figure S6E,F, Supporting Information).

**Figure 3:**
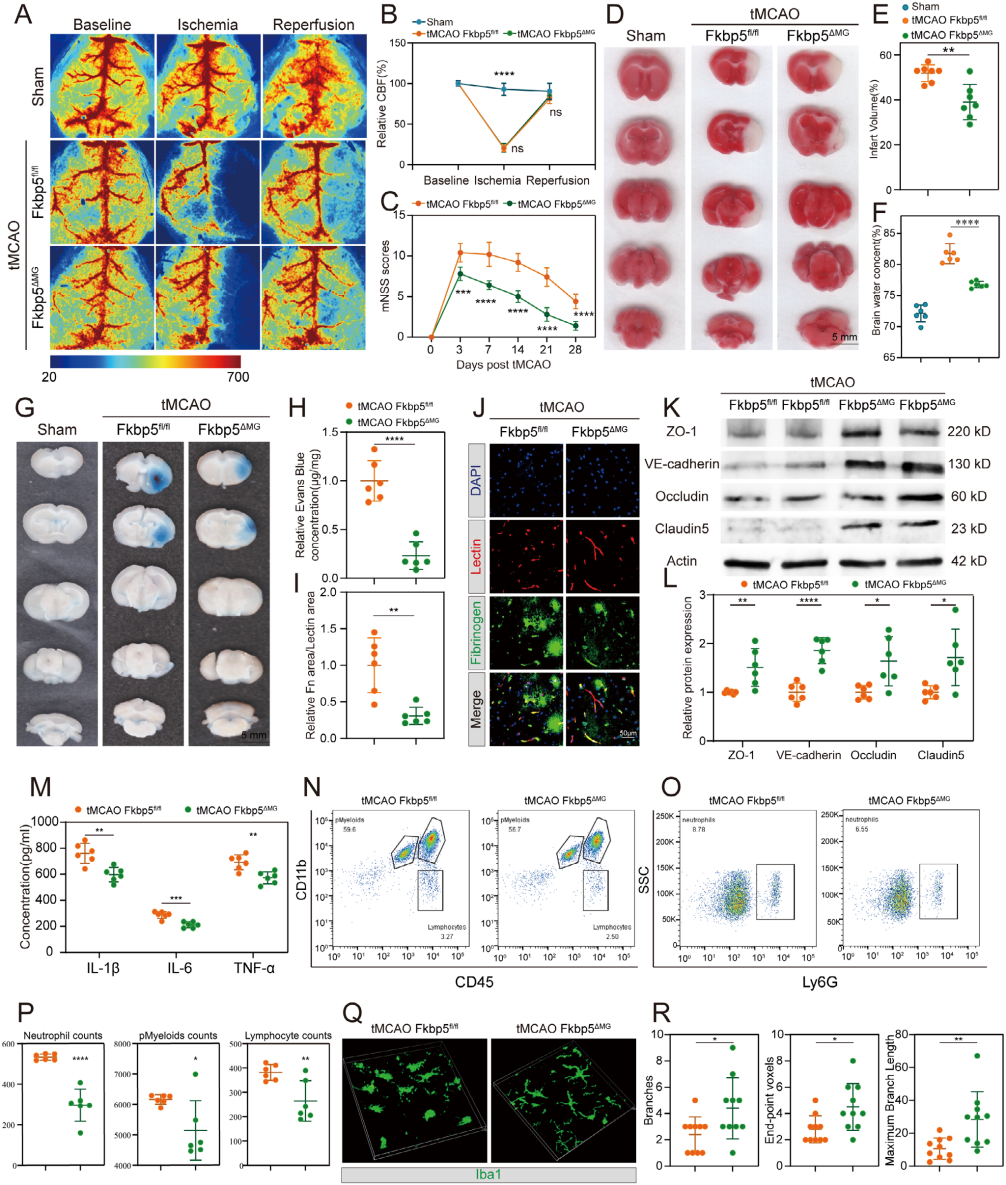
Inhibiting stroke‐VAM by Fkbp5 deletion in microglia attenuates BBB disruption and inflammation after post‐stroke. A,B) Representative images (A) and quantitative data (B) for cerebral blood flow of sham mice, tMCAO Fkbp5^fl/fl^ mice and tMCAO Fkbp5ΔMG mice using laser speckle imaging system. C) Neurological function evaluation by mNSS score system of Fkbp5^fl/fl^ mice and Fkbp5^ΔMG^ mice during 3–28 days post stroke. *n* = 5 per group. D,E) Representative pictures (D) and quantitative data (E) of infarct volume in Fkbp5^fl/fl^ mice and Fkbp5^ΔMG^ mice at 1 day after tMCAO measured by TTC staining. *n* = 7 for each group. F) Quantitative data of brain water content of sham mice, tMCAO Fkbp5^fl/fl^ mice and tMCAO Fkbp5^ΔMG^ mice. *n* = 6 for each group. G,H) Representative image (G) and quantitative data (H) of Evans blue leakage in tMCAO Fkbp5^fl/fl^ mice and tMCAO Fkbp5^ΔMG^ mice. *n *= 6 for each group. I,J) Quantitative data (I) and representative micrograph (J) of fibrinogen immunofluorescence in peri‐infarct region from tMCAO Fkbp5^fl/fl^ mice and tMCAO Fkbp5^ΔMG^ mice. *n* = 6 for each group. K,L) Representative immunoblot bands (K) and quantitative data (L) of tight junction proteins in ischemic hemisphere from control mice and microglial Fkbp5 deficient mice. *n* = 6 for each group. M) Quantitative data of pro‐inflammatory factors (IL‐1β, IL‐6, and TNF‐α) concentration by ELISA in tMCAO Fkbp5^fl/fl^ mice and tMCAO Fkbp5^ΔMG^ mice. *n* = 6 for each group. N–P) Infiltrated immune cells counts (neutrophils counts, pMyeloids counts, and lymphocytes counts) in tMCAO Fkbp5^fl/fl^ mice and tMCAO Fkbp5^ΔMG^ mice. N) Representative gating flow plots for pMyeloids and lymphocytes. O) Representative gating flow plots for neutrophils. P) Cell counts quantitative data for neutrophils, pMyeloids, and lymphocytes. *n* = 6 for each group. Q,R) Representative image (Q) and quantitative data (R) of microglia morphology in peri‐infarct region from tMCAO Fkbp5^fl/fl^ mice and tMCAO Fkbp5^ΔMG^ mice. *n* = 10 for each group. Data are presented as mean ± SD. unpaired *t*‐test; one‐way ANOVA; two‐way ANOVA; * *P* < 0.05; ** *P* < 0.01; *** *P* < 0.001; **** *P* < 0.0001. tMCAO, transient Middle Cerebral Artery Occlusion; Fkbp5^fl/fl^, *Fkbp5* ^flox/flox^ mice without Fkbp5 conditional deletion in microglia; Fkbp5^ΔMG^, *Fkbp5* ^flox/flox^ and *Cx3cr1* ^Cre^ mice with Fkbp5 conditional deletion in microglia.

Both *Fkbp5* ^fl/fl^ mice and *Fkbp5* ^ΔMG^ mice underwent successful tMCAO surgery, as monitored by laser speckle imaging. Meanwhile, laser speckle imaging revealed a similar CBF reduction in the occlusion period and a similar CBF restoration after monofilament withdrawal between the two groups (**Figure** **3**A,B). The reduction of body weight induced by tMCAO was similar in the two groups (Figure S6G, Supporting Information).

To investigate whether specific deletion of Fkbp5 in the microglia influenced the neurological recovery and infarct volume after AIS, we evaluated the neurological deficit using an 18‐point grading system during 3–28 days after tMCAO and the infarction volume by TTC staining at 1 day after tMCAO. When compared with *Fkbp5* ^fl/fl^ mice, *Fkbp5* ^ΔMG^ mice displayed a prominent and better neurological functional recovery that lasted up to 28 days post‐stroke (Figure 3C). TTC staining indicated a smaller infarct volume in *Fkbp5* ^ΔMG^ mice relative to that in *Fkbp5* ^fl/fl^ mice (Figure 3D,E). These data indicate that inhibiting stroke‐VAM by Fkbp5 deletion in the microglia could improve ischemic stroke outcome.

We next determined whether microglial‐specific Fkbp5 deletion altered post‐stroke BBB leakage and brain edema. As the Fkbp5 protein level changed most evidently at 1‐day post‐tMCAO, we observed the results at 24 h after the mice received surgery, unless stated otherwise. When compared with that in the sham *Fkbp5* ^fl/fl^ group, the brain water content in the tMCAO *Fkbp5* ^fl/fl^ group increased prominently, suggesting brain swelling and obvious breakdown of the BBB after stroke (Figure 3F). However, *Fkbp5* ^ΔMG^ mice displayed a reduction in their brain water content after tMCAO (Figure 3F). Evans blue assay and IF staining of fibrinogen (blood‐borne molecular) were conducted to assess the BBB integrity. The Evans blue leakage in the parenchyma was significantly attenuated in the *Fkbp5* ^ΔMG^ mice group after tMCAO, relative to that in the *Fkbp5* ^fl/fl^ mice group (Figure 3G,H). Fibrinogen extravasated into the cerebral parenchyma was also strongly reduced in *Fkbp5* ^ΔMG^ mice after the tMCAO surgery (Figure 3I,J). Tight junctions (ZO‐1, occludin, and claudin5) and adherens junctions (VE‐cadherin) played a critical role in guarding the BBB integrity. We noted that the deletion of microglia‐specific Fkbp5 pronouncedly reduced the protein level of ZO‐1, VE‐cadherin, occludin, and claudin5 after I/R injury (Figure 3K,L). To assess the specific role of microglial Fkbp5 in VAM, we co‐culture isolated microglia with endothelial cells to mimic their physical contact in vivo. The electrical resistance of the endothelial cell layer co‐cultured with Fkbp5‐deficient microglia was significantly higher than that of the endothelial cell layer co‐cultured with control microglia (Figure S6H, Supporting Information). FITC leakage assays also demonstrated improved barrier function in endothelial cells co‐cultured with Fkbp5‐deficient microglia (Figure S6I, Supporting Information). These results demonstrated that the deletion of microglia‐specific Fkbp5 could preserve the BBB integrity that was disrupted by I/R injury.

BBB disruption facilitates peripheral immune cell infiltration, exacerbating neuroinflammation, and further compromising BBB stability. ELISA assays demonstrated significantly reduced levels of proinflammatory cytokines (IL‐1β, IL‐6, and TNF‐α) in *Fkbp5* ^ΔMG^ mice compared with those in *Fkbp5* ^fl^/^fl^ mice after AIS (Figure 3M). Moreover, the tMCAO *Fkbp5* ^ΔMG^ group displayed markedly fewer counts of neutrophils, pMyeloid cells, and lymphocytes from the peripheral circulation when compared with the tMCAO *Fkbp5* ^fl/fl^ groups (Figure 3N–P). The gating strategy of flow cytometry is shown in Figure S7 (Supporting Information). The morphology of microglia changed from branched‐like (resting status) to amoeboid‐like (activated status) within minutes post‐stroke. Activated microglia promoted proinflammatory response and BBB degradation in ischemic stroke. We also noted that, morphologically, microglia in the peri‐infarct region of tMCAO *Fkbp5* ^ΔMG^mice retained a more ramified, branched morphology compared with the amoeboid shape typical of activated microglia in the tMCAO *Fkbp5* ^fl/fl^ group (Figure 3Q,R). These data collectively suggest that inhibition of Fkbp5‐mediated stroke‐VAM mitigates BBB breakdown and inflammatory responses following AIS.

While we noticed that myeloid cell infiltration is decreased in Fkbp5‐deficient mice generated by using *Cx3cr1‐*Cre transgenic mice after tMCAO, raising the possibility that peripheral Fkbp5 deletion contributes to the phenotype. Since both microglia and infiltrating bone marrow‐derived myeloid cells express Cx3cr1, it is not possible to distinguish between the individual contributions of microglial Fkbp5 and macrophage Fkbp5 to post‐stroke BBB disruption and inflammation. We performed bone marrow transplantation (BMT) using macrophages isolated from *Fkbp5* ^fl/fl^ or *Fkbp5* ^ΔMG^ mice (Figure S8A, Supporting Information). Recipients were *Fkbp5* ^ΔMG^ mice. This generated two chimeric cohorts: Control: *Fkbp5* ^fl/fl^→*Fkbp5* ^ΔMG^ mice (intact Fkbp5 in macrophages; Fkbp5 deficiency in microglia) Experimental: Fkbp5^ΔMG^ mice→*Fkbp5* ^ΔMG^ mice (Fkbp5 deficiency in both microglia and macrophages). Following tMCAO, flow cytometry of ischemic brains revealed no significant difference in pMyeloids cells infiltration between groups at 24 h (Figure S8B,C, Supporting Information). This suggests that Fkbp5 deletion in peripheral macrophages does not alter early immune cell distribution. Furthermore, *Fkbp5* ^ΔMG^ mice→*Fkbp5* ^ΔMG^ mice showed equivalent neurological deficits (Figure S8D, Supporting Information), comparable infarct volumes (Figure S8E,F, Supporting Information), and no exacerbated BBB disruption (Figure S8G,H, Supporting Information) versus controls. Critically, these data confirm that Fkbp5 deficiency in peripherally derived macrophages does not introduce confounding effects on acute stroke outcomes, validating the microglia‐specific mechanistic interpretations in our study.

The recovery timeline and mechanism following ischemic stroke may vary by sex. To assess whether Fkbp5‐specific knockout in microglia improves outcomes of cerebral infarction in female mice, we replicated the experiment in age‐matched female mice. The reduction of body weight induced by tMCAO was similar in age‐matched female *Fkbp5* ^fl/fl^ mice and *Fkbp5* ^ΔMG^ mice (Figure S9A, Supporting Information). Compared to *Fkbp5* ^flox/flox^ controls, female *Fkbp5* ^ΔMG^ mice exhibited lower mNSS scores (Figure S9B, Supporting Information), reduced infarct volumes (Figure S9C,D, Supporting Information), and diminished BBB leakage (Figure S9E,F, Supporting Information). Consistent with male outcomes, post‐tMCAO female *Fkbp5* ^ΔMG^ mice demonstrated enhanced proliferative status in brain endothelial cells versus female *Fkbp5* ^flox/flox^ (Figure S9G,H, Supporting Information). These results indicate that *Fkbp5* deletion promotes vascular homeostasis establishment. Collectively, our results demonstrate that microglial *Fkbp5* deletion attenuates post‐tMCAO injury severity, reduces vascular leakage, and preserves endothelial homeostasis in a sex‐independent manner.

### 2.4 Microglia‐Specific Knockout of Fkbp5 Obstructs the Energization of Stroke‐VAM in a Murine tMCAO Model

**Figure 4:**
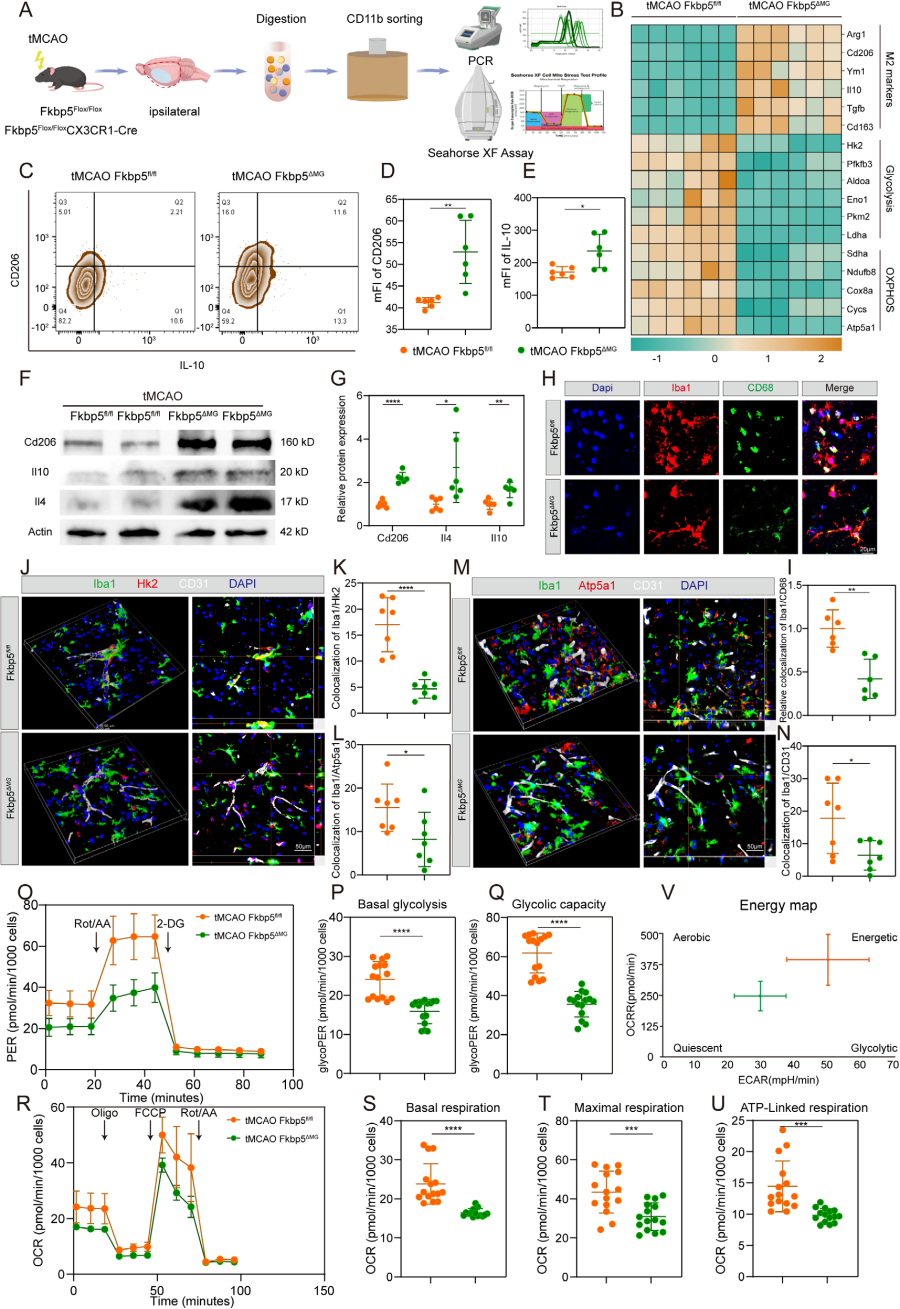
Microglia‐specific knockout of Fkbp5 obstacles the energization of stroke‐VAM in a murine tMCAO model. A) Schematic graphic of cerebral microglia isolation used for PCR and Seahorse XF assay B) qPCR data of M2 markers, glycolysis, and OXPHOS genes in ischemic cortex from Fkbp5^ΔMG^ mice relative to Fkbp5^fl/fl^ mice. *n *= 6 per group. C–E) M2 markers level (CD206 and IL‐10) in control or Fkbp5 deficient microglia using flow cytometry conducted on tMCAO Fkbp5^fl/fl^ mice and tMCAO Fkbp5^ΔMG^ mice. C) Representative flow plots of control and Fkbp5‐deficient microglia gated byCD206 and IL‐10. Quantification of the mFI of CD206 (D) and IL‐10 (E) for control and Fkbp5 CKO microglia. *n* = 6 per group. F,G) Representative immunoblots (F) and quantitative data (G) of M2 markers (CD206, IL‐10, and IL‐4) in ischemic cortex from Fkbp5^fl/fl^ mice (*n* = 6) and Fkbp5^ΔMG^ mice (*n* = 6) after ischemic stroke. H,I) Representative pictures (H) and quantitative data (I) of immunostaining of Iba1 and CD68 ischemic cortex from Fkbp5^fl/fl^ mice (*n* = 6) and Fkbp5^ΔMG^ mice (*n* = 6) after ischemic stroke. J,K) Representative immunofluorescent images (J) and quantitative analysis of Iba1 and Hk in ipsilateral peri‐infarct area of Fkbp5^fl/fl^ mice (*n* = 7) and Fkbp5^ΔMG^ mice (*n* = 7) after tMCAO. L,M) Representative immunofluorescent images (J) and quantitative analysis of Iba1 and Atp5a1 in ipsilateral peri‐infarct area of Fkbp5^fl/fl^ mice (*n* = 7) and Fkbp5^ΔMG^ mice (*n* = 7) after tMCAO. N) Colocalization immunofluorescent analysis of Iba1 and CD31. O–Q) Proton efflux rate (PER) measured by Seahorse Glycolytic Rate assay using Seahorse Extracellular Flux Analyzer in isolated CD11b^+^ microglial cells of ipsilateral hemisphere of Fkbp5^fl/fl^ mice (*n* = 5) and Fkbp5^ΔMG^ mice (*n *= 5) after tMCAO. R–U) Oxygen consumption rate (OCR) measured by mitochondrial stress test using Seahorse Extracellular Flux Analyzer in isolated CD11b^+^ microglial cells of ipsilateral hemisphere of Fkbp5^fl/fl^ mice (*n* = 5) and Fkbp5^ΔMG^ mice (*n* = 5) after tMCAO. V) Snapshot of bioenergetic profile of CD11b^+^ microglia cells of ipsilateral hemisphere of Fkbp5^fl/fl^ mice and Fkbp5^ΔMG^ mice after tMCAO. Data are presented as mean ± SD. unpaired *t*‐test; * *P* < 0.05; ** *P* < 0.01; *** *P* < 0.001; **** *P* < 0.0001. tMCAO, transient Middle Cerebral Artery Occlusion; Fkbp5^fl/fl^, *Fkbp5* ^flox/flox^ mice without Fkbp5 conditional deletion in microglia; Fkbp5^ΔMG^, *Fkbp5* ^flox/flox^ and *Cx3cr1* ^Cre^ mice with Fkbp5 conditional deletion in microglia; OXPHOS, Oxidative Phosphorylation; DAPI, 4′,6‐diamidino‐2‐phenylindole.

To explore how Fkbp5 regulates VAM after ischemic stroke, we examined stroke‐VAM‐specific metabolic characteristics in Fkbp5‐deficient microglia using the tMCAO model. Microglia were isolated from the ipsilateral cortex via magnetic‐activated cell sorting (MACS) for PCR validation and Seahorse metabolic flux analysis (**Figure** **4**A). Figure S10A (Supporting Information) shows the workflow of microglia sorting via CD11b labelling. The microglial marker (Aif1) content was enriched, and other cell markers (Rbfox3, Pecam1, and S100β) expressions were barely detectable in the labelled cells when compared to those in the unlabeled cells (Figure S10B, Supporting Information). The proportion of CD11b^+^ cells in labelled cells was dramatically greater than that in the unlabeled cells, as detected by flow cytometry (Figure S10C,D, Supporting Information), suggesting an effective collection of microglia from various cerebral cells. Importantly, Fkbp5 expression was nearly undetectable in microglia isolated from *Fkbp5* ^ΔMG^ mice compared with those from *Fkbp5* ^fl^/^fl^ mice (Figure S10E, Supporting Information), confirming effective microglia‐specific knockout of Fkbp5.

Low expression of M2 anti‐inflammatory markers and phagocytosis are well‐described signature of stroke‐VAM above. PCR analysis revealed increased anti‐inflammatory factors mRNA level and in Fkbp5‐deficient microglia from *Fkbp5* ^ΔMG^ mice as compared to that of *Fkbp5* ^fl/fl^ mice post stroke (Figure 4B), which indicates the role of Fkbp5 in inhibiting M2 subtype. After tMCAO, Fkbp5 CKO microglia showed prominently higher expression level of M2 markers (cellular membrane marker CD206 and intracellular anti‐inflammatory factor IL‐10) compared to *Fk*bp5^fl/fl^ microglia as measured by flow cytometry (Figure 4C–E). WB data showed that *Fkbp5* CKO resulted in increased protein levels of CD206, IL‐4, and IL‐10 compared to *Fkbp5* floxed in ischemic cortex (Figure 4F,G). CD68 is a typical marker representing activated phagocytosis in microglia. The phagocytotic ability of microglia was suppressed by *Fkbp5* CKO as results from IF staining of CD68 and Iba1 (Figure 4H,I). These data suggest that Fkbp5 inhibition increased anti‐inflammatory markers and reduced phagocytosis activity in stroke‐VAM post‐tMCAO.

We further explored the spatiotemporal characteristics of the elevated M2 markers in Fkbp5‐deficient stroke‐VAM through immunofluorescence staining of Arg1, a typical M2 marker. Compared to *Fkbp5* ^fl/fl^ mice, the proportion of Arg1^+^ microglia in the brain tissue of *Fkbp5* ^ΔMG^ mice was significantly higher at both 1 and 3 days after tMCAO (Figure S11A–C, Supporting Information). Spatially, the number of Arg1^+^ microglia located around blood vessels in *Fkbp5* ^ΔMG^ mice was higher than those in non‐perivascular regions (Figure S11D, Supporting Information). However, in *Fkbp5* ^fl/fl^ mice, the number of perivascular Arg1^+^ microglia was comparable to non‐perivascular Arg1^+^ microglia 1 day after cerebral infarction and lower than non‐perivascular Arg1^+^ microglia 3 days post‐infarction (Figure S11D, Supporting Information). Although Fkbp5 deficiency increased the expression of M2 markers in stroke‐VAM, we examined functional differences between Fkbp5‐deficient stroke‐VAM and the canonical M2 microglia subpopulation by comparing differentially expressed genes in single‐nucleus RNA sequencing of *Fkbp5* ^ΔMG^ mice. Genes significantly upregulated in Fkbp5‐deficient stroke‐VAM compared to the canonical M2 microglia subpopulation were enriched in positive regulation of migration, regulation of cell shape, regulation of apoptotic process, and angiogenesis (Figure S11E, Supporting Information). While genes significantly downregulated in Fkbp5‐deficient stroke‐VAM compared to the canonical M2 microglia subpopulation were enriched in endocytosis, synaptic vesicle endocytosis, chromatin remodeling, and protein transport (Figure S11F, Supporting Information). This further highlights the heterogeneity of stroke‐VAM.

Elevated capabilities of glycolysis and OXPHOS are critical metabolic characteristics of stroke‐VAM, which may provide energy supply for stroke‐VAM. The expression of glycolytic genes and *OXPHOS* genes was impaired in isolated microglia from *Fkbp5* ^ΔMG^ brain when compared to those in the microglia from *Fkbp5* ^fl/fl^ mice after stroke (Figure 4B). The expressions of Hk2 and Atp5a1 in stroke‐VAM surrounding vasculature were significantly reduced in *Fkbp5* ^ΔMG^ mice when compared with those in *Fkbp5* ^fl/fl^ mice (Figure 4J–M), indicating that Fkbp5 promoted glycolysis and OXPHOS in stroke‐VAM. As expected, the number of VAM was decreased in *Fkbp5* ^ΔMG^ penumbra when compared to that in *Fkbp5* ^fl/fl^ peri‐infarct area, as calculated from the colocalization of CD31 and Iba1 (Figure 4N). Furthermore, we evaluated the glycolytic and OXPHOS function of Fkbp5‐deficient microglia and Fkbp5‐floxed microglia isolated from the ipsilateral hemisphere by measuring the proton efflux rate (PER) through the Seahorse Glycolytic Rate assay and the oxygen consumption rate (OCR) in the Seahorse Mito Stress test separately. *Fkbp5* CKO modestly impaired PER with lower basal glycolytic rate and glycolytic capacity in microglia sorting from ischemic brain (Figure 4O–Q). *Fkbp5* CKO microglia exhibited decreased mitochondrial OXPHOS when compared to floxed microglia, which was manifested as a reduction in the basal respiration, maximal respiration, and ATP‐linked respiration (Figure 4R–U). Overall, the bioenergetics profile of floxed and CKO microglia isolated from the ischemic hemisphere demonstrated that microglia from the ipsilateral *Fkbp5* ^fl/fl^ mice brain were more energetic and with high respiration and glycolysis for their fuel demand post‐stroke (Figure 4V). In contrast, Fkbp5‐deficient microglia of tMCAO brain significantly downregulated their respiration and exhibited less glycolytic phenotype. The decline in glycolysis and oxidative phosphorylation in Fkbp5‐deficient microglia may also be attributed to reduced viability, such as the effects of apoptosis. Therefore, we performed Annexin V/PI staining on microglia isolated from *Fkbp5* ^fl/fl^ mice and *Fkbp5* ^ΔMG^ mice after ischemic stroke. Flow cytometry analysis revealed no significant difference in microglial viability between the *Fkbp5* ^ΔMG^ mice and the *Fkbp5* ^fl/fl^ mice (Figure S12A,B, Supporting Information), thus excluding increased apoptosis as a confounder.

Collectively, these results strongly suggest that microglial Fkbp5 remarkably ramp up their glycolytic and mitochondrial OXPHOS metabolism to meet the energy demands of phagocytosis in stroke‐VAM.

### 2.5 Deficiency of Microglial Fkbp5 in tMCAO Mice Improves EC Integrity and Angiogenesis

**Figure 5:**
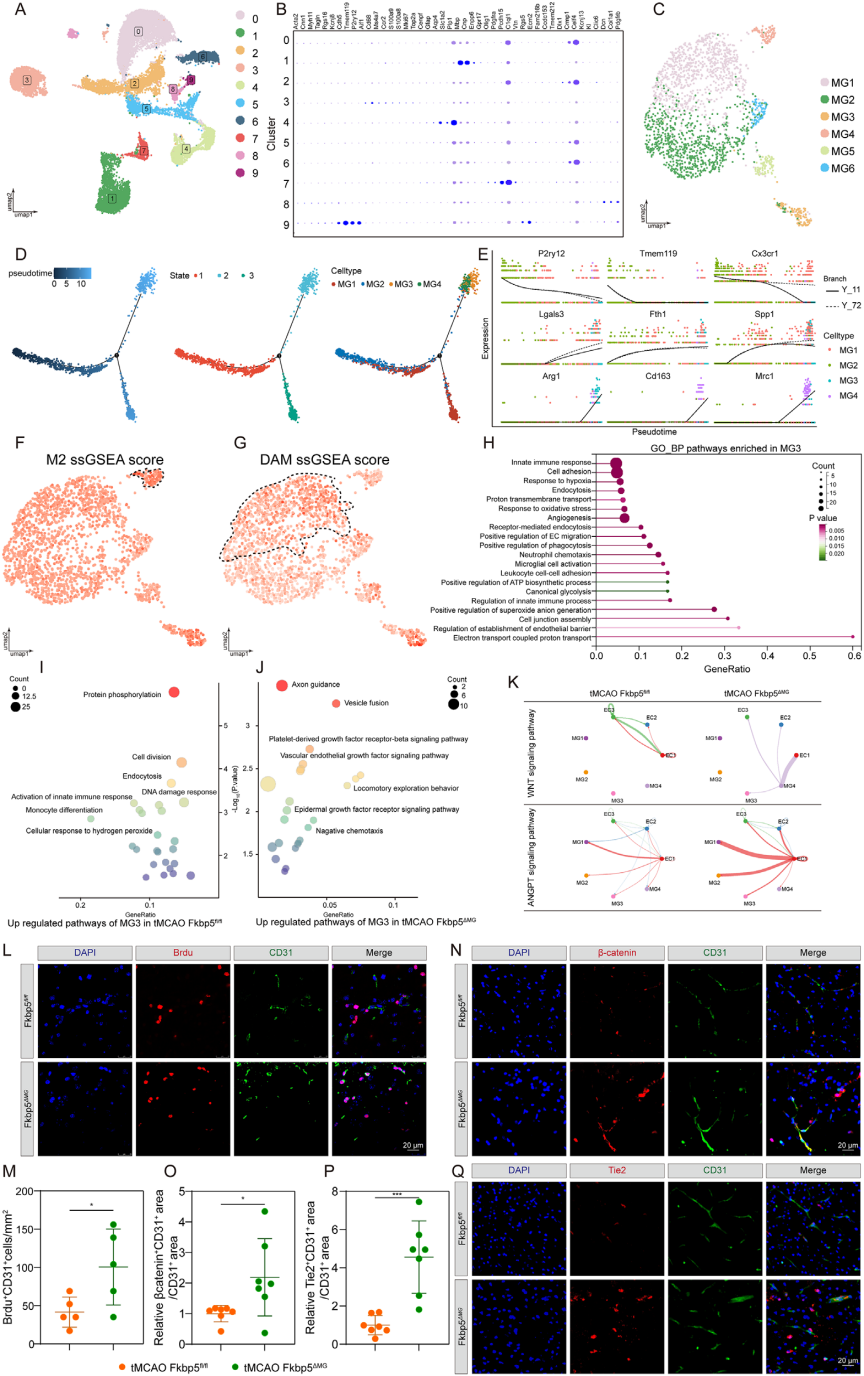
Deficiency of microglial Fkbp5 in tMCAO mice improves endothelial cell integrity and angiogenesis. A) UMAP plot visualizing and unsupervised clustering of all cells colored by cell types. B) Dot plot representing the expression level of cell type specific genes within each cluster. C) UMAP plot showing the unsupervised clustering of microglia colored by subclusters. D) Pseudotime analysis of microglia using Monocle based on tissue origin and Seurat determined clusters. E) Dot plots showing different expression of homeostatic genes (*P2ry12*, *Tmem119*, and *Cx3cr1*), DAM genes (*Lgals3*, *Fth1*, *Spp1*), and M2 genes (*Arg1*, *Cd163*, *Mrc1*) according to pseudotime order, overlapped with Seurat's original cluster colors superimposed. F) UMAP plot visualizing the M2 ssGSEA score distribution in each microglia subclusters. G) UMAP plot visualizing the DAM ssGSEA score distribution in each microglia subclusters. H) GO‐BP terms enriched for microglial subcluster MG3 under the threshold of *P*‐value < 0.05. I,J) GO‐BP terms enriched microglial subcluster MG3 for upregulation in tMCAO Fkbp5^fl/fl^ (I) in tMCAO Fkbp5^ΔMG^ (J) under the threshold of *P*‐value < 0.05. K) Signaling pathways communication between microglial cells and endothelial cells, including Wnt signaling pathway and Angpt signaling pathway. L,M) Representative immunofluorescent images (L) and quantitative analysis (M) of Brdu^+^CD31^+^ endothelial cells number in ipsilateral peri‐infarct area of Fkbp5^fl/fl^ mice (*n* = 5) and Fkbp5^ΔMG^ mice (*n *= 5) after tMCAO. N,O) Quantitative analysis (N) and representative immunofluorescent images (O) and of βcatenin and CD31 in ipsilateral peri‐infarct area of Fkbp5^fl/fl^ mice (*n* = 7) and Fkbp5^ΔMG^ mice (*n* = 7) after tMCAO. P,Q) Quantitative analysis (P) and representative immunofluorescent images (Q) and of Tie2 and CD31 in ipsilateral peri‐infarct area of Fkbp5^fl/fl^ mice (*n *= 7) and Fkbp5^ΔMG^ mice (*n* = 7) after tMCAO. Data are presented as mean ± SD. unpaired *t*‐test; * *P* < 0.05; ** *P* < 0.01; *** *P* < 0.001; **** *P* < 0.0001. UMAP, Uniform Manifold Approximation and Projection; GO‐BP, Gene Ontology: Biological Process; ssGSEA, single‐sample Gene Set Enrichment Analysis; DAM, Disease Associated Microglia; M2, M2 polarized microglia; tMCAO, transient Middle Cerebral Artery Occlusion; DAPI, 4′,6‐diamidino‐2‐phenylindole; Fkbp5^fl/fl^, *Fkbp5* ^flox/flox^ mice without Fkbp5 conditional deletion in microglia; Fkbp5^ΔMG^, *Fkbp5* ^flox/flox^ and *Cx3cr1* ^Cre^ mice with Fkbp5 conditional deletion in microglia.

Next, we examined how FFkbp5‐mediated stroke‐VAM affects vascular function after stroke. snRNA‐seq was performed on ischemic cerebral cells from *Fkbp5* ^ΔMG^ mice and *Fkbp5* ^fl/fl^ mice post‐stroke. In total, 7754 cells (1397 genes per cell in *Fkbp5* ^ΔMG^ ipsilateral hemisphere and 5812 cells with 1353 genes per cell in *Fkbp5* ^fl/fl^ ischemic brain were analyzed. Ten transcriptionally distinct clusters were identified via unsupervised clustering and visualized by UMAP (**Figure** **5**A). The expression of canonical markers was shown in a dot plot (Figure 5B). ECs and microglia were recognized by their well‐known markers, including *Cdh5*, *Pecam1*, *Vwf*, *Cldn5*, and *C1qa*, *Trem2*, *C1qc*, *Cx3cr1*, *P2ry12*, *Hexb*, *Tmem119*, respectively (Figure S13A–K, Supporting Information). Microglial cells were further re‐clustered into six subclusters (MG1–MG6) (Figure 5C). Clusters MG5 and MG6, containing fewer than 60 cells per group, were excluded from subsequent analyses. Pseudotime trajectory analysis showed that MG1, MG2, MG3, and MG4 occupied distinct positions along differentiation paths (Figure 5D). The expression patterns of homeostatic markers (*P2ry12*, *Tmem119*, *Cx3cr1*), DAM markers (*Lgals*, *Fth1*, *Spp1*), and M2 markers (*Arg1*, *Cd163*, and *Mrc1*) along branch point 1 reflected microglial fate transitions from quiescent microglia (MG2) to DAM‐like (MG1) or M2‐like (MG4) states after ischemia (Figure 5E). However, there was no prominent difference in the M1 marker expression among the four subclusters (Figure S14A,B, Supporting Information). I Consistent with the findings in pseudotime trajectories, the ssGSEA score based on specific gene signatures suggested the cellular function for each subcluster, such as “high M2 ssGSEA score” for MG4 (Figure 5F; Figure S14C, Supporting Information) and “high DAM ssGSEA score” for MG1 and MG3 (Figure 5G; Figure S14D, Supporting Information). In addition, the canonical DAM markers (*Csf1, Lilr4b*, and *Spp1*) were mainly expressed in MG1 (Figure S14E–G, Supporting Information). UMAP plot displayed homeostatic markers (*Cx3cr1*, *Tmem119*, and *P2ry12*) expression on MG2 (Figure S14H–J, Supporting Information) and M2 markers (*Il4ra*, *Cd163*, *Mrc1*) on MG4 (Figure S14K–M, Supporting Information). However, MG3 was still not defined through typical microglia subtypes. The enriched GO terms in MG3 confirmed the biological process and function of stroke‐VAM, namely “Endocytosis,” “Cell adhesion,” “proton transmembrane transport,” “angiogenesis,” “positive regulation of ATP biosynthetic process,” “canonical glycolysis,” “positive regulation of phagocytosis,” “cell junction assembly,” and “regulation of establishment of endothelial barrier” (Figure 5H) Therefore, MG3 may represent stroke‐VAM.

Next, we analyzed the role of Fkbp5 CKO on microglia function. On comparing the Fkbp5 expression on microglia between the tMCAO *Fkbp5* ^ΔMG^ group and tMCAO *Fkbp5* ^fl/fl^ group, we confirmed the effective knockout of microglial Fkbp5 (Figure S15A,B, Supporting Information). The GO term enrichment of the downregulated genes in stroke‐VAM from the tMCAO *Fkbp5* ^ΔMG^ group showed up as protein phosphorylation, cell division, endocytosis, DNA damage response, and the activation of innate immune response (Figure 5I). The upregulated genes of stroke‐VAM in the tMCAO *Fkbp5* ^ΔMG^ group compared with those in the tMCAO *Fkbp5* ^fl/fl^ group were enriched in axon guidance, angiogenesis pathway, and negative chemotaxis (Figure 5J). In addition, we performed GO‐term enrichment analysis of DEGs in the tMCAO *Fkbp5* ^ΔMG^ group versus the tMCAO *Fkbp5* ^fl/fl^ group. MG1 isolated from *Fkbp5* ^fl/fl^ ischemic brain was associated with aerobic respiration and oxidative stress (Figure S16A, Supporting Information). MG1 in *Fkbp5* ^ΔMG^ mice after tMCAO was mostly involved in the branching process involved in blood vessels morphogenesis, angiogenesis, in utero embryonic development, neurogenesis, and negative regulation of inflammatory response (Figure S16B, Supporting Information). MG2 possibly affects the cerebral microenvironment post‐stroke via inflammatory response and proliferation (Figure S16C, Supporting Information). MG2 from tMCAO *Fkbp5* ^ΔMG^ mice was associated with axon guidance and neuron development (Figure S16D, Supporting Information). MG4 in *Fkbp5* ^fl/fl^ ischemic hemisphere was involved in cell morphogenesis and calcium ion transmembrane transport (Figure S16E, Supporting Information). Fkbp5‐deficient MG4 after tMCAO potentially played a role in axonogenesis and vasculogenesis (Figure S16F, Supporting Information).

To comprehend the influence of Fkbp5‐mediated stroke‐VAM on cerebrovasculature, the cell‐chat interaction between microglia and ECs was investigated through comparison between the *Fkbp5* ^ΔMG^ group and the *Fkbp5* ^fl/fl^ group after ischemic stroke (Figure S17A, Supporting Information). Pathways maintaining the BBB integrity were enhanced in Fkbp5‐deficient ipsilateral tissues, including the Wnt‐signaling pathway and NRG‐signaling pathway (Figure 5K; Figure S17B, Supporting Information). Interestingly, some angiogenesis signaling pathways, such as Angpt, Angptl, and PDGF, were increased in *Fkbp5* ^ΔMG^ mice at day 1 after tMCAO (Figure 5K; Figure S17B, Supporting Information). These data suggested the deleterious role of microglial Fkbp5 on the BBB and angiogenesis post stroke. To explore the pro‐angiogenic capability in *Fkbp5* ^ΔMG^ mice, we recorded that the Brdu^+^CD31^+^ proliferating ECs density was significantly increased in the peri‐infarct area in *Fkbp5* ^ΔMG^ mice when compared with that in the *Fkbp5* ^fl/fl^ mic (Figure 5L,M) To validate the effect of *Fkbp5* CKO on ECs, we conducted IF of several signaling pathways in the regulation of EC functions (βcatenin for the Wnt‐signaling pathway) and angiogenesis (Tie2 for the Angpt‐signaling pathway). The expression of β‐catenin and Tie2 was significantly upregulated in the microglial Fkbp5‐deficient mice's brain when compared with that in the control mice's brain after AIS (Figure 5N–Q). Then we validate two ligand–receptor interactions (Wnt5a‐Fzd4 and Angpt1‐Tie2) between microglia and endothelial cells by proximity ligation assay (PLA) in vivo and in vitro. Compared to *Fkbp5* ^fl/fl^ mice, *Fkbp5* ^ΔMG^ mice exhibited increased Angpt1‐Tie2 (Figure S18A,B, Supporting Information) and Wnt5a‐Fzd (Figure S18C,D, Supporting Information) binding signals in the ischemic cortex. In vitro co‐culture of microglia with endothelial cells also revealed higher Angpt1‐Tie2 (Figure S18E,F, Supporting Information) and Wnt5a‐Fzd (Figure S18G,H, Supporting Information) binding PLA signal detection in endothelial cells co‐cultured with Fkbp5‐deficient microglia. Overall, these data revealed that I/R injury‐induced upregulation of Fkbp5 altered the interaction between stroke‐VAM and ECs, thereby impairing the BBB integrity and angiogenesis.

### 2.6 Fkbp5 Knockdown Inhibits the Function and Metabolic Status of Stroke‐VAM after OGD/R In Vitro

**Figure 6:**
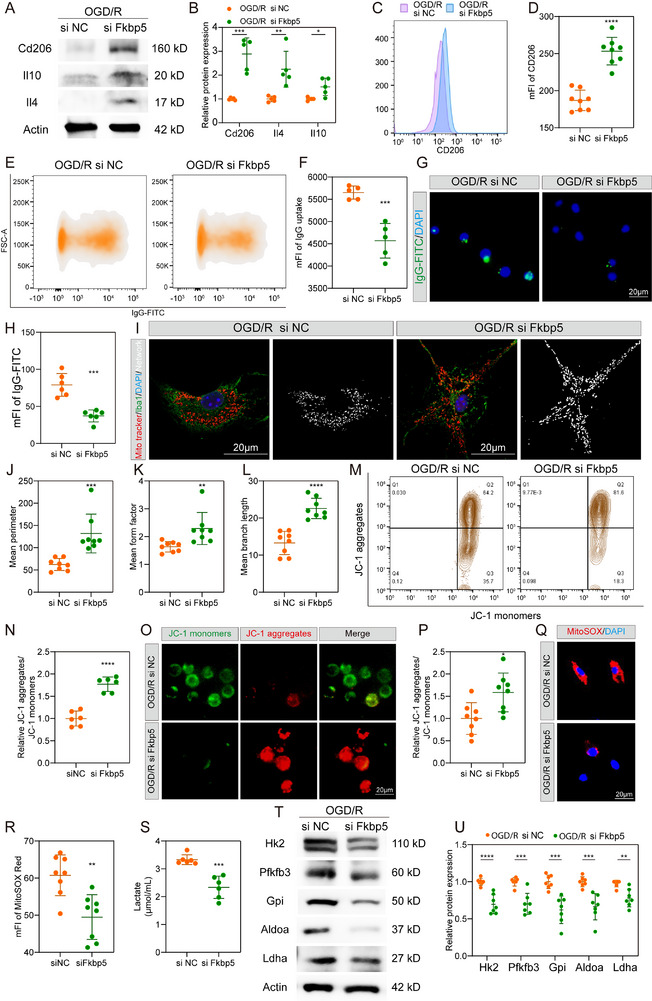
Fkbp5 knockdown inhibits the function and metabolic status of stroke‐VAM after OGD/R in vitro. A,B) Representative immunoblots images (A) and quantitative data (B) of M2 markers (CD206, IL‐10, and IL‐4) of BV2 cells receiving NT siRNA (*n* = 6) or si‐Fkbp5 siRNA (*n* = 6) under OGD/R condition. C,D) Flow plot (C) and mean fluorescence intensity (mFI) (D) of CD206 in BV2 cells receiving NT siRNA (*n* = 7) or si‐Fkbp5 siRNA (*n* = 7) before OGD/R treatment. E,F) Flow plot (E) and mean fluorescence intensity (mFI) (F) of IgG‐latex in BV2 cells after administration of NT siRNA (*n* = 5) or si‐Fkbp5 siRNA (*n* = 5) after OGD/R condition. G,H) Representative pictures (G) and quantitative data of uptake of IgG‐latex in BV2 cells after administration of NT siRNA (*n* = 6) or si‐Fkbp5 siRNA (*n* = 6) after OGD/R condition. I–L) Representative images (I) and morphological quantitative analysis (including mean perimeter (J), mean form factor (K), and mean branch length (L)) of primary microglial cells after NT siRNA or Fkbp5 knockdown treatment followed by OGD/R. *n* = 8 for each group. M,N) Representative JC‐1 flow plot (M) and relative ratio (N) of JC‐1 aggregates to JC‐1 monomers of control BV2 cells (*n *= 6) and Fkbp5‐downregulated BV2 cells (*n* = 6) after OGD/R injury. O,P) Representative micrograph (O) and quantitative data (P) of JC‐1 monomers and JC‐1 aggregates of BV2 cells receiving NT siRNA (*n* = 8) or si‐Fkbp5 siRNA (*n* = 8) under OGD/R condition. Q,R) Representative micrograph (Q) and quantitative data (R) of mitoSOX of BV2 cells receiving NT siRNA (*n* = 8) or si‐Fkbp5 siRNA (*n* = 8) under OGD/R condition. S) Lactate concentration in extracellular medium released from BV2 cells after administration of NT siRNA (*n* = 6) or si‐Fkbp5 siRNA (*n* = 6) under OGD/R condition. T,U) Representative immunoblots (T) and quantitative data (U) of glycolysis enzymes expression in BV2 cells after administration of NT siRNA (*n* = 7) or si‐Fkbp5 siRNA (*n* = 7) under OGD/R condition. Data are presented as mean ± SD. unpaired *t*‐test; * *P* < 0.05; ** *P* < 0.01; *** *P* < 0.001; **** *P* < 0.0001.OGD/R, Oxygen Glucose Deprivation/Reperfusion; DAPI, 4′,6‐diamidino‐2‐phenylindole.

To determine whether the stroke‐VAM phenotype can be modulated by Fkbp5 in vitro, we examined the effects of Fkbp5 knockdown using siRNA in an oxygen‐glucose deprivation/reoxygenation (OGD/R) model. We tested three siRNAs targeting distinct Fkbp5 sequences by incubating BV2 cells for 24 h. WB analysis showed that siFkbp5‐3 effectively reduced Fkbp5 protein expression (Figure S19A,B, Supporting Information), and this siRNA was used for subsequent experiments. Following OGD/R, Fkbp5 knockdown in BV2 cells led to a robust increase in the protein levels of anti‐inflammatory markers CD206, IL‐10, and IL‐4 (**Figure** **6**A,B). The result of CD206 flow cytometry was found to be consistent with the result of WB (Figure 6C,D), indicating the suppressed role of Fkbp5 in mediating the anti‐inflammatory response. Moreover, the phagocytosis function of BV2 cells was significantly inhibited via siFkbp5 interference by assessing the uptake of latex beads‐rabbit IgG‐FITC (Figure 6E–H). These results demonstrated the inhibitory role of Fkbp5 knockdown in stroke‐VAM function.

Next, we explored the effect of Fkbp5 on microglial metabolism. Because OXPHOS depends on mitochondrial morphology and membrane potential, we assessed mitochondrial integrity after Fkbp5 knockdown. Primary microglia with Fkbp5 knockdown displayed longer, more branched mitochondria compared to the fragmented morphology observed in control cells (Figure 6I–L). Flow cytometry and microscopy of JC‐1 staining revealed that siFkbp5 treatment restored mitochondrial membrane potential relative to negative control (NC) RNA treatment (Figure 6M–P). Additionally, mitochondrial oxidative stress was markedly lower in Fkbp5‐deficient microglia (Figure 6Q,R), indicating that Fkbp5 promotes mitochondrial dysfunction. We then measured extracellular lactate levels in BV2 cells after siRNA treatment. Fkbp5 knockdown led to reduced lactate release compared with NC‐treated cells (Figure 6S), suggesting a decline in glycolytic activity. Correspondingly, protein levels of several key glycolytic enzymes were significantly decreased in Fkbp5‐deficient BV2 cells after OGD/R (Figure 6T,U). Collectively, these findings indicate that Fkbp5 accelerates glycolytic metabolism in microglia following OGD/R.

### 2.7 Fkbp5 Stabilizes Yap1 to Induce the Energization and Formation of Stroke‐VAM through Binding Lats1

**Figure 7:**
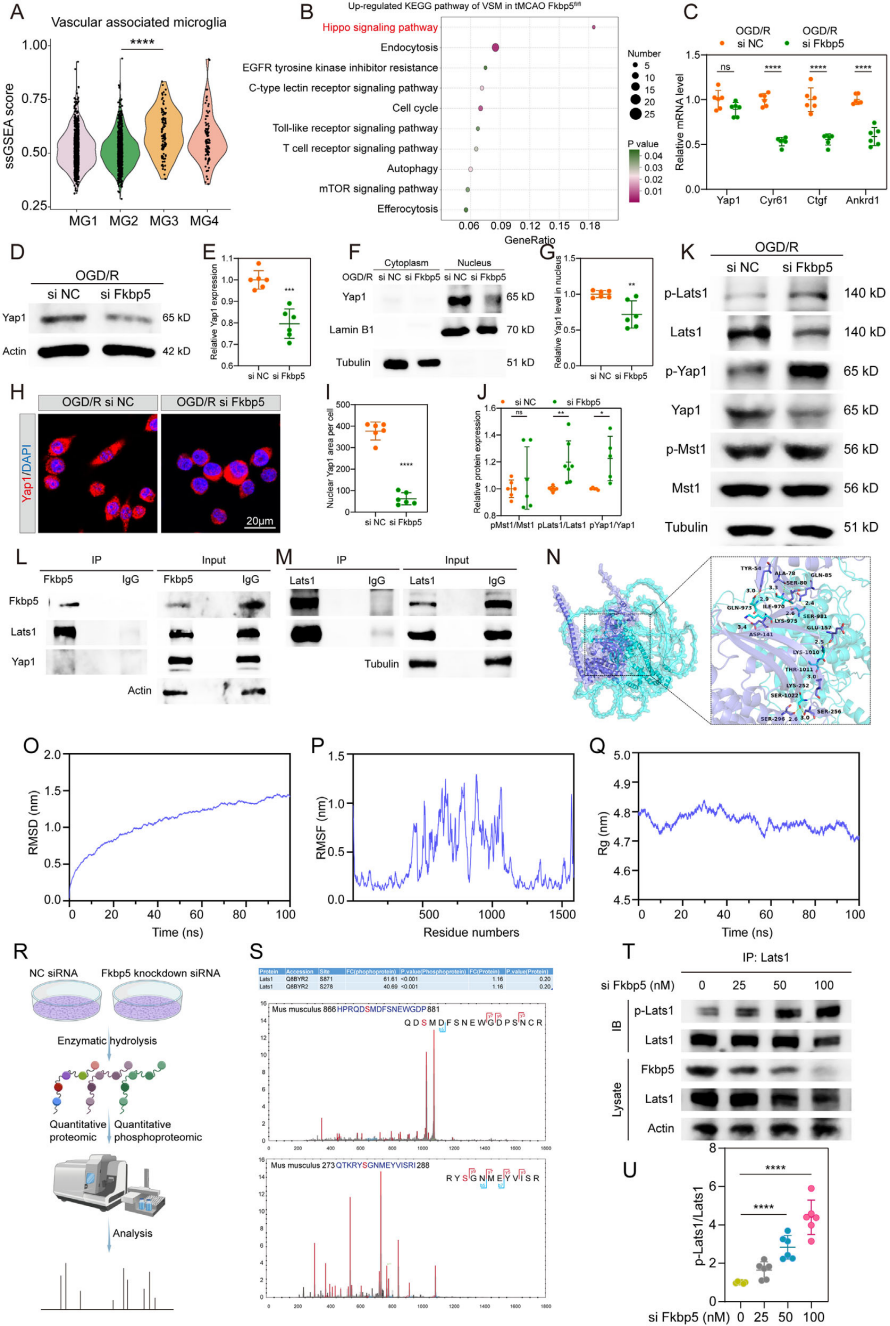
Fkbp5 stabilizes Yap1 to induce the energization and formation of stroke‐VAM through binding Lats1. A) Violin graph showing vascular associated microglia ssGSEA score in seurat based microglial subclusters in snRNA seq. B) KEGG enrichment of up regulated genes in MG3 of Fkbp5^fl/fl^ mice in relative to MG3 of Fkbp5^ΔMG^ mice. C) mRNA level of hippo signaling pathway genes (Yap1 and its downstream genes) in Fkbp5‐deficient BV2 cells in relative to control BV2 cells after I/R injury. *n* = 6 for each group. D,E) Representative immunoblots (D) and quantitative data (E) of Yap1 protein expression in Fkbp5‐deficient BV2 cells in relative to control BV2 cells after I/R injury. *n* = 6 for each group. F,G) Subcellular location of Yap1 detected by western blots in BV2 cells with or without Fkbp5 knockdown treatment after OGD/R. *n *= 6 for each group. H,I) Immunofluorescence detected the nuclear or cytoplasm location of Yap1 in BV2 cells with or without Fkbp5 knockdown treatment after OGD/R. *n* = 6 for each group. J,K) Immunoblots measured the phosphorylation ratio of Yap1 and its upstream kinase (Lats1 and Mst1) in BV2 cells with or without Fkbp5 knockdown treatment after OGD/R. *n* = 5–7 for each group. L) Immunoblot of Lats1 and Yap1 in protein complex after pulling down with anti‐Fkbp5 antibody. M) Immunoblot of Fkbp5 in protein complex after pulling down with anti‐Lats1 antibody. N) The docking prediction of Fkbp5 with Lats1. Fkbp5 was represented as a dark blue cartoon model, while Lats1 was displayed as a cyan cartoon model, with their binding sites shown as stick structures in corresponding colors. O) The root mean square deviation (RMSD) reflects the motion process of the Fkbp5‐Lats1 complex. P) The root mean square fluctuation (RMSF) reflects the flexibility of the Fkbp5‐Lats1 complex during molecular dynamics simulations. Q) The radius of gyration (Rg) reflects the compactness and degree of constraint in the Fkbp5‐Lats1 complex binding. R) Flowchart of BV2 cells treated with Fkbp5 knockdown siRNA or negative control siRNA for quantitative proteomics and quantitative phosphoproteomics analysis. S) The table showing the fold change and *P*‐value of phosphorylation site of lats1 (above). Mass spectrometry (MS) of Lats1 phosphorylated by Fkbp5 knockdown at Ser871 (middle). Mass spectrometry (MS) of Lats1 phosphorylated by Fkbp5 knockdown at Ser278 (below). T,U) Representative immunoblots and quantitative data of p‐Lats1 after gradient concentrations of Fkbp5 knockdown siRNA treatment. *n* = 6 for each group. Data are presented as mean ± SD. unpaired *t*‐test; one way ANOVA; * *P* < 0.05; ** *P* < 0.01; *** *P* < 0.001; **** *P* < 0.0001. ssGSEA, single‐sample gene set enrichment analysis; tMCAO, transient middle cerebral artery occlusion; OGD/R, oxygen glucose deprivation/reperfusion; DAPI, 4′,6‐diamidino‐2‐phenylindole.

To elucidate the mechanism underlying the regulatory role of Fkbp5 in stroke‐VAM, we compared the changes in stroke‐VAM gene expression between the *Fkbp5* ^ΔMG^ mice and *Fkbp5* ^fl/fl^ mice after AIS. The stroke‐VAM‐characteristic genes expression assessed by ssGSEA revealed the highest score in MG3 (**Figure** **7**A), indicating a representative role of MG3 in consistency with the aforementioned results. Downregulated genes of *Fkbp5* ^ΔMG^ MG3 versus *Fkbp5* ^fl/fl^ MG3 are closely related to the hippo‐signaling pathway as measured by KEGG enrichment (Figure 7B). Yap1 is an important and core transcriptional co‐activator in the hippo signaling pathway. To validate our snRNA‐seq findings, we performed PCR analysis on Yap1 and Yap1‐targeting genes from Fkbp5‐knockdown siRNA‐treated BV2 cells and NC siRNA‐treated BV2 cells after OGD/R. We found that the Yap1 mRNA level was not significantly changed after siFkbp5 interference (Figure 7C). Although the expression of downstream genes activated by Yap1, including *Cyr61*, *Ctgf*, and *Ankrd1*, was substantially reduced post‐Fkbp5 knockdown in BV2 cells (Figure 7C). However, we discovered that the downregulation of Yap1 protein was induced by Fkbp5‐knockdown siRNA administration in BV2 cells (Figure 7D,E). Yap1 translocation from the cytoplasm to the nucleus is essential for activating the downstream genes in Hippo signaling. Then, we detected the subcellular location in response to Fkbp5 knockdown. The Yap1 protein content in the cytoplasm was not different between the Fkbp5‐interference group and the NC group. However, the nucleus Yap1 protein was dramatically diminished after Fkbp5‐targeting siRNA administration as detected by WB and IF (Figure 7F–I). These data indicate that Fkbp5 promotes the Yap1 protein level and translocation without stimulating Yap1 transcription.

We wondered how Fkbp5 influences the Yap1 protein level and function. The Mst1/Lats1/Yap1 axis is responsible for Yap1 stability through post‐translational phosphorylation. Yap1 protein was degraded after being phosphorylated at Ser127 by Lats1. Lats1 was phosphorylated by upstream kinase Mst1. WB data revealed that Fkbp5‐knockdown treatment significantly increased the phosphorylation of Yap1 and Lats1 rather than that of Mst1 in BV2 cells under OGD/R conditions (Figure 7J,K). As Fkbp5 is a scaffold protein containing a tetratricopeptide repeat (TPR) domain to mediate the activity of other proteins. We accordingly set up Co‐IP experiments to investigate whether Fkbp5 binds Yap1 or Lats1 to maintain Yap1 dephosphorylation and stability. We recorded an interaction between Fkbp5 and Lats1 (but not Mst1) using Fkbp5 antibody pull‐down in BV2 cells after OGD/R (Figure 7L). By using the Lats1 antibody to pull down, the binding of Fkbp5 with Lats1 was also confirmed (Figure 7M). Moreover, the inhibitory function of Fkbp5 in Yap1 phosphorylation was identified in the tMCAO Fkbp5^ΔMG^ group when compared with that in the tMCAO Fkbp5^fl/fl^ group, as assessed by IF staining (Figure S20A,B, Supporting Information). Thus, we revealed that Fkbp5 increases Yap1 protein and translocation by binding Lats1 and inhibiting phosphorylation of Lats1 and Yap1.

To elucidate the structural domains involved in the Fkbp5‐Lats1 binding, we performed molecular docking and molecular dynamics simulations. After manually optimizing the protein structures by removing water molecules and adding hydrogen atoms using AutoDockTools‐1.5.7, a protein–protein docking was conducted using the GRAMM server. In PyMol, Fkbp5 was represented as a dark blue cartoon model, while Lats1 was displayed as a cyan cartoon model, with their binding sites shown as stick structures in corresponding colors (Figure 7N, Supporting Information). The results revealed that the FK1 domain of Fkbp5 binds to the protein interaction region of Lats1. Multiple residue pairs, such as Lys252 of Fkbp5 and Thr1011 of Lats1, formed hydrogen bonds between Fkbp5 and Lats1. The docking score for Fkbp5‐Lats1 binding was ‐725. Subsequently, we used Gromacs2020 software to perform molecular dynamics simulations on the docked complex. Within the 0‐100ns timeframe, the root mean square deviation (RMSD) values of the complex fluctuated stably within 1.5nm, indicating that the ligand could bind to the protein and maintain a relatively stable state (Figure 7O). The overall root mean square fluctuation (RMSF) values of the complex were low, suggesting high structural stability (Figure 7P). Additionally, the protein radius of gyration (Rg) curve fluctuated between 4.7‐4.85nm during 0‐100ns, demonstrating the formation of a tightly bound and stable complex (Figure 7Q).

To explore how Fkbp5 alters the phosphorylation state of Lats1, we conducted quantitative proteomics and quantitative phosphoproteomics on BV2 cell protein lysates with and without Fkbp5 knockdown (Figure 7R). A total of 3,752 associated proteins were co‐detected (Figure S20C, Supporting Information). Bioinformatics enrichment analysis was performed on the differentially phosphorylated proteins after Fkbp5 knockdown using the Gene Ontology (GO) database (Figure S20D,E, Supporting Information). The results revealed that the differentially phosphorylated proteins were significantly enriched in Protein serine/threonine kinase activity and Protein serine kinase activity (Figure S20F, Supporting Information). Further analysis revealed that Fkbp5 knockdown significantly increased phosphorylation at two sites of Lats1: S278 and S871 (Figure 7S). Among these, the phosphorylation state of Lats1 S871 showed the most significant change, and the S871 site is located within the serine/threonine kinase domain (catalytic core region) of Lats1, likely serving as the primary site through which Fkbp5 reduces downstream Yap1 phosphorylation. Transfecting a series of gradient concentrations of Fkbp5‐targeting siRNAs into BV2 cells demonstrated that Fkbp5 knockdown promotes Lats1 phosphorylation in a dose‐dependent manner (Figure 7T,U). In summary, these results suggest that Fkbp5 binds to the protein interaction region of Lats1 via its FK1 domain, thereby inhibiting phosphorylation at the S278 and S871 sites of Lats1 in a dose‐dependent manner.

To also explore whether Fkbp5 induces stroke‐VAM energization and formation by reserving Yap1 translocation. For this purpose, we used a plasmid to overexpress Yap1 before administration of Fkbp5 interference. We first identified the efficacy of Yap1 overexpression by plasmid (Figure S21A,B, Supporting Information). The mRNA level of M2 molecules (CD206, IL‐4, and IL‐10) was prominently increased in the Fkbp5‐knockdown group, but not in the Fkbp5‐knockdown group with Yap1‐overexpression group (Figure S21C, Supporting Information). Compared with NC siRNA‐treated BV2 cells, microglia in the Fkbp5 KO siRNA group showed decreased uptake of latex IgG FITC, which was upregulated in the Fkbp5 KO siRNA and Yap1 OE plasmid group (Figure S21D,E, Supporting Information). These data indicate that the inhibited stroke‐VAM function by Fkbp5 knockdown was restored via pre‐administration of Yap1 overexpression. Moreover, the branched and reticulated mitochondria were recovered by Fkbp5 deficiency in BV2 cells. Pre‐treatment with plasmid performed to upregulate Yap1 expression impaired the microglial mitochondrial morphology (Figure S21F–I, Supporting Information). The mitochondrial ROS was elevated by Yap1 overexpression, which was reduced by Fkbp5 knockdown (Figure S21J,K, Supporting Information). Meanwhile, glycolytic enzyme expression was increased by Yap1 overexpression and Fkbp5‐knockdown treatment when compared with Fkbp5 knockdown treatment alone (Figure S21L,M, Supporting Information). Overall, these data demonstrate that Fkbp5 inhibition suppressed the formation and energization of stroke‐VAM by downregulating the Yap1 protein level.

However, it remains unclear whether the increased glycolysis and oxidative phosphorylation in stroke‐VAM are caused by mechanistic coupling or co‐activated by Yap1. We detected the expression of oxidative phosphorylation enzymes in BV2 cells overexpressing Yap1 after adding the glycolysis inhibitor 2‐DG. The expression levels of Ndufb8, Atp5a1, and Cox8a showed no difference in the 2‐DG‐treated group compared to the vehicle group (Figure S22A, Supporting Information). Similarly, compared to the control plasmid‐treated group, the glycolytic enzyme mRNA levels were significantly elevated in the Yap1‐overexpressing plasmid‐treated group (Figure S22B, Supporting Information). However, treatment with the oxidative phosphorylation inhibitor RotAA failed to reverse the Yap1‐mediated increase in glycolytic enzymes (Figure S22B, Supporting Information). These results suggest that the simultaneous elevation of glycolysis and oxidative phosphorylation in microglia is co‐activated by Yap1, rather than representing a mechanistic coupling of the two metabolic pathways. To further validate the Yap1‐mediated co‐activation phenomenon in the stroke‐VAM, we stereotaxically injected Adeno‐Associated Virus (AAV) viruses specifically overexpressing YAP1 into microglia in the mouse cortex. Three weeks later, immunofluorescence revealed that EGFP was localized exclusively to microglia (Figure S22C, Supporting Information). QRT‐PCR analysis revealed greatly elevated Yap1 mRNA levels in the ipsilateral mouse cortex following AAV‐mediated Yap1 overexpression compared to control AAV treatment (Figure S22D, Supporting Information). Compared to VAMs from mice injected with control AAV virus, stroke‐VAMs overexpressing Yap1 showed significantly increased expression of the glycolytic key enzyme Hk2 (Figure S22E,F, Supporting Information) and the oxidative phosphorylation key enzyme Atp5a1 (Figure S22G,H, Supporting Information), as revealed by immunofluorescence staining.

### 2.8 Pharmacological Inhibition of Fkbp5 by SAFit2 Improved the Stroke Outcome by Inhibiting Stroke‐VAM

**Figure 8:**
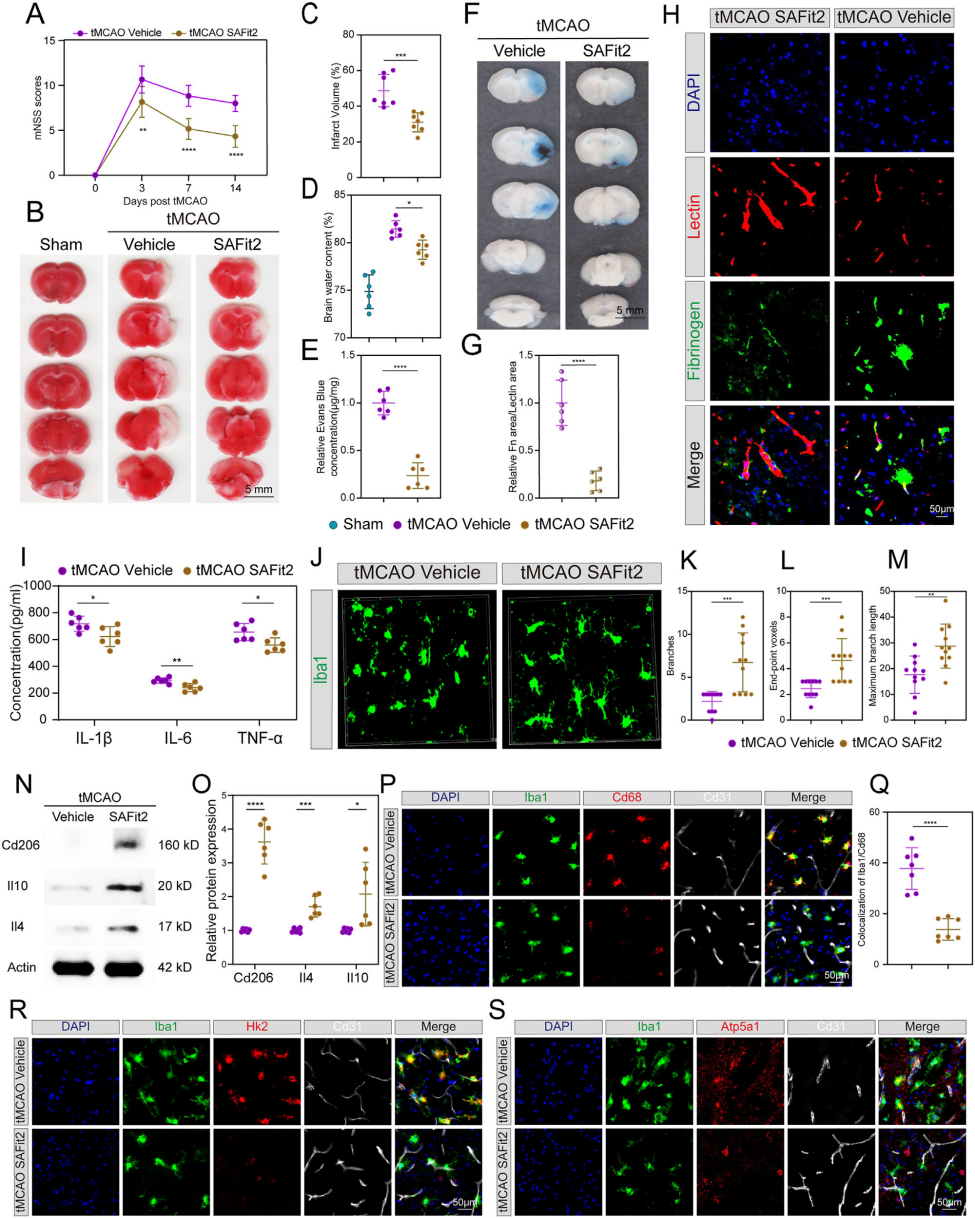
Pharmacological inhibition of Fkbp5 by SAFit2 improved the stroke outcome by inhibiting stroke‐VAM. A) Neurological function evaluation by mNSS score system in tMCAO mice receiving vehicle or SAFit2 during 3–28 days post stroke. *n* = 5 per group. B,C) Representative pictures (B) and quantitative data (C) of infarct volume in tMCAO mice receiving vehicle or SAFit2 at 1 day after tMCAO measured by TTC staining. *n* = 7 for each group. D) Quantitative data of brain water content of sham mice, tMCAO vehicle mice and tMCAO SAFit2 mice. *n* = 6 for each group. E,F) Quantitative data (E) and representative image (F) of Evans blue leakage in tMCAO mice receiving vehicle or SAFit2. *n* = 6 for each group. G,H) Quantitative data (G) and representative micrograph (H) of fibrinogen immunofluorescence in peri‐infarct region from mice with vehicle or SAFit2 treatment after ischemic stroke. *n* = 6 for each group. I) Quantitative data of pro‐inflammatory factors (IL‐1β, IL‐6, and TNF‐α) concentration by ELISA in tMCAO vehicle mice and tMCAO SAFit2 mice. *n* = 6 for each group. J–M) Representative 3D reconstructed image (J) and quantitative data (K—M) of microglia morphology in peri‐infarct region from mice with vehicle or SAFit2 treatment after tMCAO. *n* = 11 for each group. N,O) Representative immunoblots (N) and quantitative data (O) of M2 markers (CD206, IL‐10, and IL‐4) in ischemic cortex from mice injected with vehicle (*n *= 6) and mice injected with SAFit2 (*n* = 6) after ischemic stroke. P,Q) Representative pictures (P) and quantitative data (Q) of immunostaining of Iba1, CD68, and Cd31 ischemic cortex from mice administrated with vehicle (*n* = 7) or SAFit2 (*n* = 7) after ischemic stroke. R) Representative pictures of immunostaining of Iba1, Hk2, and Cd31 ischemic cortex from mice administrated with vehicle or SAFit2 after ischemic stroke. S) Representative pictures of immunostaining of Iba1, Atp5a1, and Cd31 ischemic cortex from mice administrated with vehicle or SAFit2 after ischemic stroke. Data are presented as mean ± SD. unpaired *t*‐test; two‐way ANOVA; * *P* < 0.05; ** *P* < 0.01; *** *P* < 0.001; **** *P* < 0.0001. tMCAO, transient Middle Cerebral Artery Occlusion; DAPI, 4′,6‐diamidino‐2‐phenylindole.

Based on the deleterious role of microglial Fkbp5 in ischemic stroke from the aforementioned analysis, we sought a potential therapy targeting Fkbp5 in attenuating I/R injury. SAFit2 is a novel and specific Fkbp5 inhibitor that can ameliorate neuroinflammation in neuropathic pain.^[^ ^22^ ^]^ To evaluate whether SAFit2 is a promising candidate for AIS treatment, SAFit2 (10 mg kg^−1^) was intraperitoneally administered in mice at 4 h after tMCAO, and the stroke outcome was evaluated at 1 day after the stroke onset. SAFit2 administration could significantly improve the neurological function in tMCAO mice when compared to that in the vehicle treatment group (**Figure** **8**A). The mice that received SAFit2 therapy displayed a smaller infarct volume compared with the mice who received the vehicle (Figure 8B,C). The brain water content in the tMCAO SAFit2 group was remarkably lower than that in the tMCAO‐vehicle group (Figure 8D), indicating that SAFit2 attenuates I/R injury‐induced brain swelling. Consistently, the extravasation of Evans blue and fibrinogen was robustly decreased in the mice injected with SAFit2 when compared with that in the mice injected with the vehicle (Figure 8E–H). The concentration of pro‐inflammatory factors (IL‐1β, IL‐6, and TNF‐α) in the ipsilateral cortex was remarkedly decreased by SAFit2 administration (Figure 8I). Microglial morphology in the peri‐infarct area turned into more branched and slender and less round after SAFit2 injection (Figure 8J–M). Therefore, SAFit2 reduced BBB permeability, inflammation, and infarct volume, as well as promoted neurological recovery in a murine model of tMCAO. Then we further set experiments to evaluate the influence of SAFit2 on stroke‐VAM after I/R injury. The protein expression of M2 markers (i.e., CD206, IL‐4, and IL‐10) was prominently elevated in the mice with SAFit2 when compared with that in the vehicle mice (Figure 8N,O). Meanwhile, the phagocytotic activity of stroke‐VAM was inhibited by SAFit treatment as measured by IF colocalization of Iba1 and CD68 (Figure 8P,Q). Furthermore, we examined the effect of SAFit2 on the metabolic state of stroke‐VAM. Compared to the vehicle group, mice treated with SAFit2 showed significantly reduced expression levels of glycolytic enzyme (Hk2) and oxidative phosphorylation enzyme (Atp5a1) in stroke‐VAM (Figure 8R,S; Figure S23A,B, Supporting Information). This suggests that SAFit2 can suppress the high‐energy metabolic pattern characterized by elevated glycolysis and oxidative phosphorylation in stroke‐VAM. Additionally, the number of stroke‐VAMs was lower in the SAFit2‐treated group than in the vehicle group (Figure S23C, Supporting Information). Overall, these results indicated the protective and therapeutic potential of SAFit2 in stroke outcome by attenuating stroke‐VAM function.

Given the transformative potential of targeting Fkbp5, we explored whether delayed SAFit2 administration could still provide protective effects on stroke outcomes. We administered a single intraperitoneal injection of SAFit2 at either 24h or 72h after tMCAO surgery and evaluated stroke outcomes on day 5, including neurological deficits and infarct volume. Our findings showed that SAFit2 administration, whether delayed to 24h (Figure S24A–C, Supporting Information) or 72h (Figure S24D–F, Supporting Information), reduced neurological scores and infarct volume after acute ischemic stroke. Thus, the therapeutic time window for SAFit2 can be extended up to 72 hours in the murine tMCAO model, providing a foundation for clinical translation research of SAFit2.

## 3 Discussion

This study explored the contribution of microglia to BBB leakage and neovascularization following AIS, aiming to identify potential therapeutic targets. We identified a novel stroke‐VAM population, characterized by low expression of M2 markers and elevated phagocytosis, glycolysis, and OXPHOS. These cells forming a perivascular niche. Fkbp5 is a key element regulating the process that leads to disrupted BBB and attenuated neovascularization, increased glycolysis and OXPHOS in stroke‐VAM. Specific Fkbp5 deletion in microglia significantly attenuated BBB disruption and promoted neovascularization after AIS. Furthermore, the effect of Fkbp5 on stroke‐VAM activity was mediated by its binding to Lats1, inhibiting it from phosphorylating and binding to Yap1, thereby enabling Yap1 translocation from the cytoplasm to the nucleus. Our results indicate that targeting the Fkbp5‐mediated stroke‐VAM niche is a promising interventive strategy to improve post‐ischemic vascular integrity and development.

Our finding that stroke‐VAM phagocytose ECs and downregulate TJ proteins in response to stroke is largely consistent with previous reports. Using intravital two‐photon microscopy of CX3CR+/GFP mice with tMCAO, Jolivel et al. revealed that microglia migrate to injured vessels and engulf the ECs.^[^ ^10^ ^]^ The accumulation of microglia in the perivascular niche could be attributed to the chemotaxis of parenchymal microglia by blood‐borne molecules, such as fibrinogen and albumin.^[^ ^10^ ^]^ This perivascular microglia population may also arise from increased proliferation GO enrichment analysis of DEGs in stroke‐VAM between tMCAO and a sham treatment found upregulation of genes related to the positive regulation of cell migration and cell cycle. Therefore, investigating which route dominantly accounts for perivascular microglia niche formation could be important for manipulating microglia behavior after ischemic stroke. M1‐polarized microglia induced by I/R injury in the penumbra were reported to downregulate TJ proteins by secreting pro‐inflammatory factors such as IL‐1β and TNF‐α.^[^ ^23^, ^24^ ^]^ However, scRNA sequencing detected no sign of M1‐polarization (ranked ninth among 12 clusters) or M2‐polarization (ranked twelfth among 12 clusters) in stroke‐VAM, which contradicts previous studies. This discrepancy might be due to the precise re‐clustering of microglia subtypes by scRNA sequencing.

Using Fkbp5 deletion and snRNA‐seq analysis of ischemic hemispheres in *Cx3cr1* ^cre^ *Fkbp5* ^flox/flox^ and *Fkbp5* ^flox/flox^ mice, we revealed the pro‐endothelial survival profile of Fkbp5‐deficient stroke‐VAM. Unlike previous studies, we found that Fkbp5‐deficient stroke‐VAM tightly interacted with ECs through the Wnt, Neuregulin (Nrg), Angiopoietin‐like (Angptl), and Platelet‐derived growth factor (Pdgf) signaling pathways. These signaling pathways are fundamental for EC proliferation and BBB integrity during embryonic development and post‐ischemic neovascularization.^[^ ^25^, ^26^, ^27^, ^28^, ^29^, ^30^, ^31^ ^]^ After colonizing the brain at mouse embryonic day 9.5 (E9.5), microglia along blood vessels interact with ECs to assist vascular sprouting and anastomosis.^[^ ^32^, ^33^ ^]^ Activation of the Wnt or Nrg signaling pathways by the exogenous supplementation of Wnt, Nrg, or PDGF‐BB ligand or agonists reduced BBB disruption and enhanced angiogenesis and recovery after I/R injury.^[^ ^25^, ^31^, ^34^, ^35^, ^36^ ^]^ However, open questions remain about the spatiotemporal dynamics of the interaction between microglia and EC and how this interaction orchestrates BBB disruption and revascularization throughout the ischemic stroke course. Our data suggest that many changes in the interaction between stroke‐VAM and ECs during vascular damage and regeneration after a stroke represent untapped therapeutic targets that should not be neglected in future investigations.

Diverse immune effector responses across all immune cells rely on their specific energy metabolism patterns. Numerous studies have demonstrated that homeostatic and M2‐polarized microglia prefer to produce ATP via OXPHOS, whereas M1‐polarized microglia in ischemic stroke mainly rely on glycolysis.^[^ ^37^ ^]^ The abundant energy supply required for amyloid‐beta‐phagocytic activity in the microglia of patients with Alzheimer's disease is ensured by shifting energy metabolism from glycolysis to OXPHOS.^[^ ^20^, ^38^ ^]^ Surprisingly, using an unbiased scRNA‐seq dataset and experimental validation, we found that glycolysis and OXPHOS in stroke‐VAM were higher than in sham‐VAM or other stroke‐activated microglia clusters. The overall energetic status in the stroke‐VAM differs from the microglia metabolism reported previously. The enhanced glycolysis may result from hypoxic stimulation after the ischemic insult.^[^ ^39^ ^]^ The elevated OXPHOS may be due to the high energy demand of phagocytosis and the stroke‐VAM perivascular position. Oxygen and glucose become available again after reperfusion.^[^ ^40^ ^]^ Therefore, the increased OXPHOS activity and high oxygen consumption could account for the elevated mitochondrial oxidative stress and ROS production in stroke‐VAM observed in our study. Moreover, Song et al., based on Seahorse extracellular flux analysis, reported that OXPHOS and glycolysis in pooled microglia from the ischemic stroke hemisphere were higher than in microglia from the non‐lesion hemisphere,^[^ ^41^ ^]^ which aligns with our findings. Therefore, our results comprehensively described the metabolic spectrum of stroke‐VAM, indicating that microglial metabolic reprogramming is largely dependent on unique disease contexts and specific effector functions.

Our findings indicate that Fkbp5 is a key modulator of two important features of stroke‐VAM: Fkbp5‐mediated stroke‐VAM accelerates BBB disruption in the subacute phase and impairs neovascularization in the chronic phase, and Fkbp5 promotes glycolysis and OXPHOS in stroke‐VAM. FK506 binding protein 5 (FKBP5), a molecular chaperone, is gaining increasing attention in metabolic and neurodegenerative diseases due to its pleiotropy effects and mechanisms.^[^ ^42^, ^43^ ^]^ Microglial Fkbp5 accelerates dopaminergic neuron death in Parkinson's disease and promotes activating of the NF‐κB signaling pathway in neuropathic pain, thus emerging as a promising therapeutic target for AIS.^[^ ^22^, ^43^ ^]^ However, the role of microglial Fkbp5 in ischemic stroke remains elusive. We found that conditional Fkbp5 knockout in microglia significantly improved stroke outcomes, including better neurological behavior, smaller infarct volume, less BBB leakage, and enhanced neovascularization. Given that the Fkbp5 knockout mediated by CX3CR1‐cre tool mice can occur in both microglia and peripherally infiltrated macrophages, we examined the impact of Fkbp5 knockout in peripheral myeloid cells on stroke outcomes through bone marrow transplantation, thereby excluding interference from peripheral Fkbp5 in the experiments. Additionally, previous studies using green fluorescent protein transgenic bone marrow chimeric mice demonstrated that microglia are significantly activated within one day after stroke and even become the predominant component of macrophages in the ischemic hemisphere by days 4‐7.^[^ ^44^, ^45^ ^]^ However, blood‐derived macrophages are scarcely observed two days post‐stroke and peak by day 7. These findings confirm the unique role of microglial Fkbp5 in stroke outcomes during the acute phase of cerebral infarction. Yu et al. demonstrated that under OGD/R, neuronal Fkbp5 exacerbates autophagy‐induced cell death through the Akt/Foxo3 pathway.^[^ ^46^ ^]^ Furthermore, we identified the therapeutic potential of SAFit2, a unique Fkbp5 inhibitor, as it can alleviate neurological deficits, reduce infarction size, and counteract BBB leakage and inflammation after ischemic stroke. Based on these findings, it is suggested that the protective role of SAFit2 is achieved by inhibiting neuronal death and mitigating the detrimental effects of stroke‐VAM. Our results provide essential preclinical evidence on the effects of SAFit2 in patients with AIS; however, further study is warranted.

The data presented here suggest that the promotive effect of Fkbp5 on stroke‐VAM depends on suppressing Yap1 phosphorylation and increasing Yap1 nuclear translocation. Multiple studies demonstrated that Fkbp5, a scaffolding protein, can regulate several kinase cascades by binding to diverse target proteins through its tetratricopeptide repeat structure.^[^ ^42^ ^]^ Kusumanchi et al. found that upregulated Fkbp5 interacts with Mst1, an upstream kinase of Yap1, to inhibit its phosphatase activity and promote Yap1 dephosphorylation and nuclear translocation in patients with chronic alcohol consumption.^[^ ^47^ ^]^ Similarly, we found that Fkbp5 binds to Lats1, the other upstream kinase of Yap1 (but not Mst1), to affect Yap1 function. Yap1, a co‐activator, enhances glycolysis by binding to HIF‐1α and TEAD1 to increase the transcription of glycolytic enzymes (e.g., Hk2, Gpi, Pfkfb3, Aldoa, pgk1, Eno1, and Ldha) in various cells and diseases.^[^ ^48^, ^49^, ^50^ ^]^ Furthermore, Yap1 promotes mitochondrial OXPHOS in tumors by upregulating the expression of mitochondrial proteins (e.g., Lars2) and restraining the expression of mitochondrial fission molecules (e.g., Drp1).^[^ ^51^, ^52^ ^]^ Recent studies have demonstrated that Yap1 accelerates M1 polarization and inhibits M2 polarization to amplify inflammation in myeloid cells through the activation of NLRP3 or the upregulation of IL‐6,^[^ ^53^, ^54^, ^55^, ^56^ ^]^ which partially corroborates our result. The present study elucidated a comprehensive mechanism underlying Fkbp5‐mediated stroke‐VAM activity.

## 4 Experimental Section

### scRNA‐Seq Data Download and Re‐Analysis

scRNA‐seq data (GSE 174574) were downloaded from the Gene Expression Omnibus database (https://www.ncbi.nlm.nih.gov/). The GSE 174574 dataset contained single cells isolated from the tMCAO ipsilateral brain and sham ipsilateral brain at one day after MCAO or sham surgery. Then the dataset, including principal component analysis, UMAP reduction, clustering, differential expression genes (DEGs) analysis, cell type annotation, and gene enrichment was reanalyzed. The visualization of data was conducted by R software (version 3.4.0) and Seurat. Cell type identification was performed according to classic markers reported previously and the CellMarker database (http://xteam.xbio.top/ACT/). The threshold for gene enrichment (GO Biological Processes and KEGG Pathway) of DEGs was *P* < 0.05 and the absolute log2FC > 0.5. Single‐sample gene set enrichment analysis (ssGSEA) was conducted to compare the expression level of microglia classic subtype (M1, M2, and DAM) characteristic genes and metabolic (glycolysis, tricarboxylic acid cycle, and oxidative phosphorylation) characteristic genes between different microglia clusters. Gene lists used to conduct ssGSEA were summarized by previous studies or downloaded by the Gene Set Enrichment Analysis database (https://www.gsea‐msigdb.org/gsea/index.jsp), details in Table S1 (Supporting Information). Analysis scripts and intermediate data are provided in Supporting Information, details in Files S1 and S2 (Supporting Information) respectively. Reproducibility can be achieved following the reproducibility checklist (Table S3, Supporting Information).

### Animals

The adult C57BL/6J male mice (8‐12 weeks old, 20‐25g) and 3‐days‐old C57BL/6 mice were purchased from Beijing Vital River. 3‐days‐old C57BL/6 mice were used to obtain primary mural microglia according to procedures described below. *Fkbp5* ^flox/flox^ mice and *Cx3cr1* ^Cre^ mice were obtained from Cyagen Biosciences Inc. (Santa Clara, CA) and The Jackson Laboratory separately. Microglia‐specific inactivation of *Fkbp5* was achieved by cross‐bleeding *Fkbp5* ^flox/flox^ mice with *Cx3cr1* ^Cre^ mice. All transgenic mice were on a C57BL/6J background. Mice were raised in specific‐pathogen‐free conditions with free food and water, at a 12h light/dark cycle, and at standard temperature. All mouse experiments were in accordance with and approved by the Institutional Committee of Animal Care and Use (4476), and the Medical Ethics Committee of Huazhong University of Science and Technology, Wuhan, China.

### Transient Middle Cerebral Artery Occlusion (tMCAO)

Mice were randomly and blindly subjected to tMCAO or sham operation as reported previously.^[^ ^57^ ^]^ Briefly, mice were anesthetized induced by 3% isoflurane and maintained by 1.5% isoflurane. Then, the mice were fixed to exposure their ventral sides. An upper abdominal incision was made in the middle of the mouse's neck, and the skin and underlying muscle fascia layers were separated to expose the right common carotid artery and external carotid artery (ECA). A 6‐0 silicon rubber‐coated monofilament (YuShun biotechnology, China) was carefully inserted into the right ECA and advanced to the right MCA through turning at the bifurcation of ICA and ECA. The MCA was blocked for 1 h to induce transient cerebral ischemia. Subsequently, the monofilament was withdrawn to realize blood flow reperfusion. The laser speckle instrument was used to identify the success occlusion and reperfusion of MCA. Mice were included in subsequent experiments only if the CBF was reduced to 25% of baseline at the occlusion period and recovered to more than 50% of baseline upon reperfusion. Mice in the sham group received the same treatment except for monofilament insertion and remove. The rectal temperature of mice was maintained at 37±0.5 °C throughout the surgical operation. This study utilized a total of 802 mice, with a tMCAO model success rate of 88.9%. The post‐operative mortality rate for mice after tMCAO surgery was 9.3%.

### Modified Neurological Severity Score (mNSS) Assessment

mNSS was used to evaluate the neurological deficit severity and sensorimotor function recovery of mice suffered from ischemia/reperfusion injury.^[^ ^23^ ^]^ mNSS assessed the multiple aspects, including sensory, motor, reflexes, and balance. The higher the scores, the greater the injury.

### Laser Speckle Imaging

Laser speckle imaging was used to assess the success of tMCAO operation and the MCA occlusion degree of different groups by measuring mural cortical cerebral blood flow (CBF) at 3 time points (before surgery, 10 mins after occlusion, and 10 mins after reperfusion). Briefly, mice were anesthetized and placed under the laser speckle camera. CBF was measured and captured by a laser speckle imaging instrument (Reward, China). An individual blinded to the experimental design analyzed the CBF using RFLSI software. The relative CBF change (%) was calculated as: ipsilateral CBF at occlusion or at reperfusion/ipsilateral CBF pre‐ischemia × 100%.

### TTC Staining and Infarct Volume Measurement

After transcranial perfusion with PBS, the brain was gently obtained and washed in PBS, and then placed at −20 °C for 20 min to reduce the fragility of brain tissue. The brain was further cut into 2 mm thick coronal slices and incubated in 2% 2,3,5‐triphenyl tetrazolium chloride (TTC) (G3005, Solarbio, China) at the dark, 37 °C for 20 min. TTC could react with mitochondrial succinate dehydrogenase in living cells rather than dead cells, to produce red formazan. Therefore, the infarct region area, which appeared white, could be calculated according to a previous study:^[^ ^23^ ^]^ [(contralateral hemispheric area— uninjured ipsilateral hemispheric area)/(contralateral hemisphere area)] × 100%.

### Brain Water Content

After transcranial perfusion with PBS, the brain was removed to weight for wet weight. After placed in an environment at 85 °C for 2 days, the brain tissue was weighted for dry weight. The formula previously reported was used to calculate the brain water content (%): (wet weight‐ dry weight)/wet weight × 100%.^[^ ^31^ ^]^

### Evans Blue Leakage

Evans Blue dye (2%, 4mL kg^−1^) (Sigma Aldrich, USA) was injected via the tail vein to mice at 1‐day post‐stroke. After 4‐hour circulation of evans blue dye, mice were anesthetized, euthanized, and perfused with PBS. The brain was obtained, weighted, and then fixed in 4% PFA for 1 day at 4 °C. The brain was cut into 5 slices (2mm per slice) and photographed. Then the slices were homogenized and incubated in 1mL formamide (F103362, Aladdin, China) at 65 °C for 48 hours at the dark. After centrifuging at 4000 rpm for 5 min, the supernatant was aspirated and measured the absorbance at 610nm.

### Immunofluorescence

Sample preparation from brain tissue and cells. The brain tissue was fixed in 4% PFA at 4 °C overnight, and then soaked in 10%, 20%, and 30% sucrose solution for 24 hours separately. The infarct area was cut into 2mm coronal slices and embedded in OCT to obtain frozen sections (15 µm). For cellular immunofluorescence, cells were seeded in the confocal dish at propriated density. After treated with corresponding conditions, cells were harvested and washed with PBS three times. Then cells were fixed in 4% PFA at room temperature for 30 min.

Immunofluorescence procedure for brain sections and cells. To immunofluorescence stain, brain tissue slices were incubated with sodium citrate buffer (C1010, Solarbio, China) at 100 °C for 30 min to expose the antigen epitope. The sections and cells were incubated with 0.5% triton‐100 (IT9100, Solarbio, China) and 10% normal donkey serum (SL050, Solarbio, China) for 30 min to permeabilize the membrane and block nonspecific antigen. Then brain sections and cells were incubated with primary antibodies at 4 °C for one night. Details of primary antibodies for immunofluorescence are summarized in Table S4 (Supporting Information). The sections and cells were incubated with secondary fluorescent‐conjugated antibodies at room temperature for 2 h in the dark. The secondary antibodies used in the study are listed in Table S4 (Supporting Information). Finally, the sections and cells were incubated with DAPI (C1006, Beyotime, China) for 10 min before mounted with anti‐fluorescence quenching mounting solution (P0126, Beyotime, China).

### Western Blot (WB)

After euthanized and perfused with PBS, the ipsilateral cerebral cortex was harvested and homogenized in 500 µL RIPA lysis buffer, mixed with 50 × cocktail (Beyotime, China). Cells were also collected and lysed in RIPA buffer. After centrifuged, the supernatants were collected and the protein concentration was measured by the BCA protein assay kit (Beyotime, China). For nuclear and cytoplasmic protein separation, the manufactory's instruction was followed by a Nuclear and Cytoplasmic Protein Extraction Kit (Beyotime, China). An equal amount of protein was prepared from each sample to conduct a western blot. Then proteins were loaded and separated on SDS‐PAGE, and transferred to a PVDF membrane (Millipore, Germany). Followed by blocking in 5% skim milk at room temperature for 1 h, membranes were cut into an appropriate size at corresponding molecular weights and then incubated with the indicated primary antibody at 4 °C overnight. All primary antibodies used in this study are summarized in Table S4 (Supporting Information). The membranes were washed with TBST 3 times, probed with secondary antibodies, and visualized by ECL (NCMcell and Molecular Biotech, China) the next day.

### Co‐Immunoprecipitation (Co‐IP)

Co‐IP was conducted following the manuals of Pierce Co‐Immunoprecipitation (Co‐IP) Kit (26149, Thermo Scientific, USA). Briefly, an equal amount of protein (2 mg) was extracted for the Co‐IP assay, and an equal small amount of protein (30 µg) was prepared for WB to examine the input protein. The Co‐IP cell lysate was mixed with Fkbp5 antibody, Lats1 antibody, or control rabbit IgG immobilized beads overnight at 4 °C keep rotating. The input protein and immune complex were used to conduct WB the next day.

### qRT‐PCR

RNA isolation from the cerebral cortex in the ipsilateral hemisphere and cells that underwent OGD/R was achieved through homogenizing in the total RNA extraction reagent (Vazyme). cDNA was synthesized by using ABScript III RT Master Mix for qPCR (RK204429, Abclonal, China). Real‐time PCR was conducted through 2× Universal SYBR Green Fast qPCR Mix (RK21203, Abclonal, China). Table S5 (Supporting Information) shows primers used in this study.

### Enzyme‐Linked Immunosorbent Assay (ELISA)

The ischemic cerebral cortex was extracted and homogenized in cold PBS. Then the mixture was centrifuged for 5 min, and the supernatants were placed in a new tube for further detection. The concentration of IL‐6, IL‐1β, and TNF‐α was measured according to the manuals (RK00008, RK00006, RK00027; Abclonal, China). The protein concentration of each sample was also detected as mentioned above. The final results were normalized to the protein concentration.

### Magnetic‐Activated Microglia Sorting

Mice were euthanized by overdose of pentobarbital and transcardially perfused with cold PBS. The ipsilateral hemisphere was harvested with the removal of meninges and cerebellum. The brain tissue was cut into pieces as small as possible with sharp scissors on ice. The small brain pieces were immersed in a digestive enzyme mixture (TrypLE Express Enzyme, 12605010, Gibco, USA; Papain, P8150‐5, Solarbio, China) at 37 °C for 30 min with gentle and slow mixing every 3 min. Then brain tissue mixed suspension was filtered through a 70 µm cell strainer (Biosharp, China). The filtered suspension was further centrifuged at 2000 rpm for 10 min at 4 °C. The supernatant was removed and the cell pellet was gently resuspended in 9 mL 30% isotonic density gradient solution (Percoll and 10× PBS, Biosharp, China). The suspension was immediately centrifuged at 400×g with acceleration at level 3 and brake at level 1, for 20 min. The upper myelin layer and the middle supernatant layer were gently and carefully discarded. The cell pellet was resuspended with 10 mL cold HBSS, washed, and centrifuged at 400×g for 5 min. Resuspend the cell pellet for further microglia sorting, flow cytometry, and microglia culture. Microglia were isolated by MACS using a MojoSort mouse CD11b selection kit (480109, Biolegend, USA) and MojoSort Magnet (480019, Biolegend, USA). Briefly, the cell suspension was resuspended in MojoSort buffer and mixed with 10 µL biotin‐CD11b antibody cocktail and incubated for 15 min on ice. After washed one time, the cell suspension was incubated with 10 µL streptavidin nanobeads on ice for 15 min. Cells were centrifuged at 400 × g for 5 min and discarded supernatant. Resuspended the cell pellet with 2.5 mL buffer and placed the tube in the magnet for 5 min. The unlabeled fraction was poured out and collected to detect the yield and purity of sorting. Resuspended the remained cells with 2.5 mL buffer and then kept the labelled CD11b^+^ cells for further investigation.

### Flow Cytometry

Single cell suspensions were obtained from the ipsilateral hemisphere at 1‐day post‐stroke as mentioned above. To analyze the infiltrated immune cells and microglia subtype, cells were incubated with fluorescein‐conjugated antibodies at 4 °C for 30 min protecting from light. Detailed information of antibodies is displayed in Table S2 (Supporting Information). At least 20 000 events were recorded from each sample. FlowJo software was used to analyze the data.

### Primary Microglia Isolation and Cell Culture

Single cell pellets from C57BL/6J mice burn within 3 days were prepared as described above. Resuspended and seeded these cells in DMEM/F12 containing 12% fetal bovine serum and 1% penicillin and streptomycin. Cultured these cells in a humidified chamber with 5%CO_2_ at 37 °C for 14 days. The primary cortical mixed glial cells were shaken at 200 × g for 3 h to collect loosely attached microglia. Isolated microglia were cultured in poly‐*D*‐lysine coated 12‐well plates and confocal dishes for further studies. The purity of microglia was over 95% as identified by Iba1 immunofluorescent staining. BV2 cells were cultured in DMEM containing 12% fetal bovine serum and 1% penicillin and streptomycin.

### Mitochondrial Respiration

Microglia from the ipsilateral hemisphere were obtained following the procedure mentioned above. Seahorse XF Cell Mito Stress Test Kit (1030015‐100, Agilent, USA) was used to measure mitochondrial respiration function following the user's guidelines. Microglia were seed on the cell culture plate. The following reagents were added to microglia wells in order: oligomycin (1.5 µm), FCCP (1 µm), rotenone/antimycin A (0.5 µm). Protein concentration of each well was detected as described above. Mitochondrial function was normalized to protein content.

### Glycolytic Rate Assay

Microglia from the ipsilateral hemisphere were obtained following the procedure mentioned above. Seahorse XF Glycolytic Rate Assay Kit (103344‐100, Agilent, USA) was used to measure glycolytic rate following the user's guidelines. Microglia were seed on the cell culture plate. The following reagents were added to microglia wells in order: rotenone/antimycin A (0.5 µm), 2‐DG (50 mm). Protein concentration of each well was detected as described above. Glycolytic rate was normalized to protein content.

### snRNA‐Seq and Analysis

The ipsilateral cortex from 3 mice in the same group was mixed in a tube to avoid individual differences. Brain tissue was immediately transferred to liquid nitrogen after isolation. The sample was transported in frozen CO_2_. The nuclei isolation, single‐nucleus RNA library construction, and sequencing were performed by Hangzhou Kaitai Biotechnology Co., Ltd. (China). Briefly, the tissue samples were minced into pieces and immediately immersed into 2 mL HB buffer (260 mm sucrose, 30 mm KCl, 10 mm MgCl2, 20 mm Tricine‐KOH(pH7.8), 0.5 mm Spermidine, 0.15 mm Spermine, 1 mm DTT, 0.3% NP‐40, 0.2 U µL^−1^ RNase inhibitor, 1× cOmplete Protease Inhibitor Cocktail). After incubation for 10 min and homogenized by Dounce tissue grinder, the lysates were filtered by 40 µm cell strainers and centrifuged at 500 g for 5 min at 4 °C. The resuspended extracts were purified with a gradient iodixanol solution. The nuclei band, at 30–40% Iodixanol interface, was aspirated no more than 200 µL. After wash and purifying, the single‐nucleus suspensions were converted to barcoded snRNA‐seq libraries through steps including droplet encapsulation, emulsion breakage, mRNA‐captured beads collection, reverse transcription, cDNA amplification, and purification. Agilent Bioanalyzer 2100 and Qubit ssDNA Assay Kit (Thermo Fisher Scientific) were applied to qualify. Constructed libraries were sequenced by the DNBSEQ‐T7 sequencing platform with pair‐end sequencing. The sequencing data were analyzed through an open‐source pipeline (https://github.com/MGI‐tech‐bioinformatics/DNBelab_C_Series_scRNA‐analysis‐software). PISA was used to calculate the cellular gene expression and create a matrix for each library.

### Oxygen Glucose Deprivation/Reperfusion (OGD/R)

OGD/R was used to simulate ischemia/reperfusion injury in vitro. Primary microglia and BV2 cells were placed in a hypoxia chamber with 94% N_2_, 1%O_2_, 5%CO_2_ when changing the culture medium to glucose‐free, serum‐free DMEM. OGD for 6 h, medium was replaced with the previous condition containing serum and glucose. Cells were in an environment containing 20% O_2_ and 5% CO_2_ at the same time. After 6 h of OGD and 12 h of reperfusion, cells were collected for further experiments.

### RNA Interference and Plasmid Transfection

siRNA to knockdown Fkbp5 and a negative control were purchased from RiboBio (China). Plasmid to overexpression Yap1 and a negative control were purchased from Obio (China). The sequence identified efficacy to knockdown Fkbp5 expression was: GCTCCGAGAGTACAACAAA. The plasmid sequence to overexpression Yap1 was: pCDNA3.1‐CMV‐Yap1‐3 × Flag‐hGHpolya‐EF1a‐EGFP. For siRNA interference, siRNA was added at a concentration of 50 nm with transfection reagent (Ribo, China) for 24 h. While plasmid was transfected with lipofectamine 3000 (L3000075, Invitrogen, USA) for 24 h. The efficacy of Fkbp5 knockdown and Yap1 overexpression was confirmed by Western blot.

### Phagocytosis Assay In Vitro

The phagocytotic capability of microglia was measured in vitro by using Phagocytosis Assay Kit (IgG FITC) (500290, Cayman, USA). Briefly, BV2 cells were incubated with latex beads‐rabbit IgG‐FITC complex at 37 °C for 1 h in the dark after treatment. The cells were incubated with trypan blue quenching solution for 2 min and washed with PBS to quench beads simply binding at the cellular surface. Finally, cells were removed from wells to FACS tubes for further flow cytometry.

### Mitochondrial Morphology Staining

MitoTracker Red CMXRos (M7512, Invitrogen, USA) was used to label the mitochondria morphology of live cells in vitro. After OGD/R, MitoTracker probes were added to the microglia culture medium at a working concentration of 200 nm. The incubation lasts for 40 min followed by a wash with PBS. After staining, pictures were immediately taken by a confocal microscope.

### Mitochondrial Membrane Potential Assay

Enhanced mitochondrial membrane potential assay kit with JC‐1 (C2003S, Beyotime, China) was used to measure mitochondrial membrane potential according to the user's guidelines. JC‐1 formation changed from aggregates (red) to monomers (green) with the decrease of mitochondrial membrane potential.

### Mitochondrial Oxidative Stress

Mitochondrial oxidative stress level was detected through MitoSOX Red Mitochondrial Superoxide Indicators (M36008, Invitrogen, USA). Briefly, cells were incubated with MitoSOX red reagent for 30 min at 37 °C in the dark, followed by wash gently 3 times. Mitochondrial superoxide level was recorded by a confocal microscope.

### Lactate Content Assay

Cellular culture media were collected to detect the lactate content released by BV2 cells in different conditions. The lactate concentration of the extracellular medium was measured by using the Lactic Acid (LA) Content Assay Kit (BC2235, Solarbio, China). The protocol was described in detail in the manufactory's manual. Also, the protein concentration of BV2 cells in each well was measured to normalize the lactate concentration for each sample.

### Patients

This retrospective study utilized data and plasma samples from the Multicenter Clinical Trial of Revascularization Treatment for Acute Ischemic Stroke (TRAIS). The TRAIS study was a multicenter, retrospective cohort that enrolled 1278 acute ischemic stroke (AIS) patients who received intravenous thrombolysis (IVT) at five comprehensive stroke centers in China between January 2022 and July 2025. The study was conducted in accordance with the Declaration of Helsinki and was approved by the Ethics Committee of Union Hospital, Tongji Medical College, Huazhong University of Science and Technology (Registration number: ChiCTR2000033456). Written informed consent was obtained from all participants. For the present investigation, a nested case‐control study was designed within the TRAIS cohort. From the original population, 130 patients were first excluded according to the predefined criteria (e.g., chronic hepatitis, abnormal liver/renal function, loss to follow‐up), leaving 588 participants for analysis. From this pool, 92 patients were identified with a poor functional outcome at 90 days, defined as a modified Rankin Scale (mRS) score of 3 to 6. Subsequently, a 1:1 propensity score matching (PSM) was performed to select 92 patients with a good functional outcome (mRS score 0–2) who were most comparable to the poor‐outcome group. The matching variables included age, sex, and key vascular risk factors (hypertension, diabetes mellitus, hyperlipidemia, and coronary artery disease). This process yielded a final analytical sample of 184 participants (92 matched pairs).

### Transendothelial Electrical Resistance (TEER)

First, bEnd.3 cells were seeded on the upper layer of Transwell inserts (24‐well plate, pore size 0.4 µm; Corning, USA) and cultured for 2 weeks until TEER readings stabilized. Then, microglia isolated from the infarcted brain tissue of tMCAO Fkbp5^fl/fl^ mice and tMCAO Fkbp5^ΔMG^ mice were seeded on the underside of the inserts, allowing co‐culture of microglia and endothelial cells for 24 h before measuring changes in TEER readings. The transendothelial electrical resistance (TEER) values were measured using an epithelial volt‐ohmmeter (ERS‐2, Millipore, Billerica, USA).

### Fluorescein Isothiocyanate (FITC)

The preceding steps were the same as for TEER. After co‐culturing endothelial cells with microglia, FITC‐labeled 70kDa dextran (FITC‐dextran, 1:50; Sigma Aldrich) was added to the upper chamber of the insert. One hour after FITC administration, 50 µL samples were collected from the non‐luminal side of the insert. The fluorescence intensity of FITC permeating into the lower chamber was measured at appropriate excitation/emission wavelengths (488/510) using a Victor Wallac 1420 multilabel plate reader (PerkinElmer, Waltham, USA).

### Stereotactic Injection

Mural cerebral with Yap1 overexpressing in microglia were generated by stereotactic injection of adeno‐associated virus (AAV). Yap1‐overexpression AAVs (rAAV‐IBA1(1678)‐yap1‐2a‐EGFP‐WPREs‐4 × MIR‐9T, BrainVTA, China) or control AAVs (rAAV‐IBA1(1678)‐EGFP‐WPREs‐4 × MIR‐9T, BrainVTA, China) were injected into the right cortex. The procedure and injection site were conducted as previously described. The microelectrode was maintained in situ for 10 min after injection to avoid virus efflux. The final amount of viral injected into the cerebral cortex was 2.4E + 9 vg mice^−1^

### Molecular Dynamics Simulation

The Charmm36 force field was selected for proteins, Gaff2 for ligands, and the TIP3P water model was used to solvate the protein–ligand system, with sodium and chloride ions added to neutralize the system's charge. The steepest descent algorithm was employed for energy minimization, followed by 2000 ps of constrained NVT and NPT equilibration to achieve system stability. NPT equilibration was performed after NVT to stabilize the system pressure. Subsequently, a 100 ns MD simulation was conducted at 300 K, with trajectories saved every 10 ps. For further analysis, trajectory‐based RMSD, RMSF, and protein radius of gyration (Rg) were calculated.

### Molecular Docking

The predicted structures of FKBP5 and LATS1 were generated by AlphaFold. To ensure the accuracy of docking results, AutoDockTools‐1.5.7 was subsequently used to manually optimize both protein structures by removing water molecules and adding hydrogen atoms. The protein–protein docking was then performed using the GRAMM docking server.^[^ ^2^, ^3^ ^]^ The resulting protein–protein complex was similarly optimized using AutoDockTools‐1.5.7 for water removal and hydrogen addition. Finally, PyMOL was employed to predict protein interactions and generate protein–protein interaction diagrams. In PyMOL, FKBP5 was represented as a cartoon model in dark blue, LATS1 was displayed as a cyan cartoon model, and their binding sites were shown as stick structures in corresponding colors. When focusing on the binding region, the binding sites were highlighted in the color of their respective proteins.

### Quantitative Proteomics and Quantitative Phosphoproteomics

Quantitative proteomics and quantitative phosphoproteomics were performed by Shanghai OE Biotech Co., Ltd. (Shanghai, China). Briefly, BV2 cells were treated with Fkbp5 knockdown siRNA or control siRNA for 24 h, collected, washed once with PBS, centrifuged, and the supernatant was discarded. The pellet was quickly frozen in liquid nitrogen. Cell protein samples were transported on dry ice. After protein extraction, 10 µg of each sample was subjected to SDS‐polyacrylamide gel electrophoresis and Coomassie Brilliant Blue staining to confirm sample quality. Phosphoproteomics required IMAC enrichment of phosphorylated peptides. All data were acquired using a Thermo Fisher Scientific Vanquish Neo ultra‐high‐performance liquid chromatography system coupled with an Orbitrap Astral mass spectrometer equipped with a Thermo Scientific EasySpray source. Separation was achieved using a trapping column (300 µm × 0.5 cm, 5 µm, Thermo Fisher Scientific) combined with a C18 analytical column ES906 (PepMap Neo UHPLC 150 µm × 15 cm, 2 µm, Thermo Fisher Scientific). LC‐MS/MS raw data were imported into DIA‐NN (version 1.8.1) for analysis, with peptide identification false discovery rate controlled below 1%.

### Proximity Ligation Assay (PLA)

In situ analysis of ligand–receptor binding between microglia and endothelial cells was performed using the Proximity Ligation Assay (PLA) kit (Sigma‐Aldrich, USA), following the manufacturer's protocol. Specifically, fresh frozen mouse tissue sections or cell samples were fixed and permeabilized. After primary antibody incubation, species‐specific secondary antibodies conjugated with oligonucleotides (PLA probe anti‐rabbit Plus, Cat. No. DUO92002‐30RXN; anti‐mouse Minus, Cat. No. DUO92004‐30RXN) were applied to the samples at 37 °C for 1.5 h. Following the ligation step (1 h, 37 °C), antibody‐bound oligonucleotides formed DNA circles. The resulting DNA circles underwent rolling circle amplification and were hybridized with fluorophore‐labeled nucleotides at 37 °C for 2 h. Sections were mounted using Fluoroshield mounting medium containing DAPI (Cat. No. DUO82040‐5ML).

### Statistical Analysis

All experiments applied an unbiased design, randomized allocation, and blinded conduction and analysis. Data were first checked for normality using the Shapiro–Wilk test and for homogeneity of variance using Levene's test. Sample size estimation was predicated based on an initial experiment using software (v3.1, G*power) to achieve an 80% power level and a 5% significance level (two‐sided).^[^ ^58^ ^]^ Data were analyzed by GraphPad Prism (La Jolla, USA) and presented as mean±standard deviation (mean ± SD) unless specified otherwise. The sample size (*n*) was indicated in the figure legends. For normally distributed data with equal variances, the unpaired Student's *t*‐test was used to determine statistical significance when comparing two groups, while one‐way ANOVA followed by Bonferroni *post hoc* correction was applied for more than two groups comparison. Paired *t‐*tests were used to assess weight changes in the same mouse before and after tMCAO surgery. The two‐way ANOVA with Bonferroni *post hoc* correction was applied for the data with a time point. Non‐parametric data analysis was performed with Mann–Whitney *U*‐test. Statistical analyses accounted for the matched‐pairs study design. Data were presented as mean ± SD, median (IQR), or frequencies (%) as appropriate. Given the skewed distribution, serum FKBP5 concentrations were natural‐log‐transformed for parametric analyses. The association between log‐transformed FKBP5 and baseline NIHSS score was assessed using a linear mixed‐effects model, with matched‐pair ID as a random intercept. The association with 90‐day functional outcome (good [mRS 0–2] versus poor [mRS 3–6]) was evaluated using conditional logistic regression, stratified by matched‐pair ID. Results were reported as odds ratios (OR) with 95% confidence intervals (CIs). Pearson correlation coefficient was used to detect the Correlation effect. The threshold for statistical significance was *P*‐value < 0.05 in all cases. This study strictly adhered to the ARRIVE 2.0 guidelines (for animal experiments) and the MIQE guidelines (for qPCR experiments). The complete checklist is provided in Tables S6 and S7.

## Conflict of Interest

The authors declare no conflict of interest.

## Supporting information



Supporting Information

Supplemental Table 1

Supplemental data

## Data Availability

The data that support the findings of this study are available from the corresponding author upon reasonable request.
