# Supplementary material for: Microglial Fkbp5 Impairs Post‐Stroke Vascular Integrity and Regeneration by Promoting Yap1‐Mediated Glycolysis and Oxidative Phosphorylation
Source: Adv Sci (Weinh). 2025 Dec 8;13(16):e12499. doi: 10.1002/advs.202512499 (PMC13042415; doi:10.1002/advs.202512499)
Supplement: Supplementary file 1 — Supporting Information [file ADVS-13-e12499-s001.pdf]

Supplemental Figures and Tables

Microglial Fkbp5 Impairs Post-Stroke Vascular Integrity and Regeneration by Promoting Yap1 mediated Glycolysis and Oxidative Phosphorylation.

Yanan Li[1#], Yanmei Qiu[1#], Yunlei Yang[2,3,4,5], Yanhao Wei[1], Haokun Peng[1], Longhai Zeng[1], Pengcheng Li[6], Rentang Bi[1*], Bo Hu[1*].

[1]Department of Neurology, Union Hospital, Tongji Medical College, Huazhong University of Science and Technology, Wuhan, 430022, China.
[2]Department of Medicine, Division of Endocrinology, Albert Einstein College of Medicine, Bronx, United States.
[3]Department of Neuroscience, Albert Einstein College of Medicine, Bronx, New York, United States.
[4]Einstein-Mount Sinai Diabetes Research Center, Albert Einstein College of Medicine, Bronx, New York, United States.
[5]The Fleischer Institute for Diabetes and Metabolism, Albert Einstein College of Medicine, Bronx, New York, United States.
[6]Department of Ophthalmology, Union Hospital, Tongji Medical College, Huazhong University of Science and Technology, Wuhan, 430022, China.

[#]These authors contributed equally to this work.

[*]**Corresponding author:**
Bo Hu,
Department of Neurology, Union Hospital, Tongji Medical College, Huazhong University of Science and Technology, Wuhan 430022, China.
Tel: +86-13707114863
E-mail: hubo@hust.edu.cn

Rentang Bi,
Department of Neurology, Union Hospital, Tongji Medical College, Huazhong University of Science and Technology, Wuhan 430022, China.
E-mail: birt@hust.edu.cn

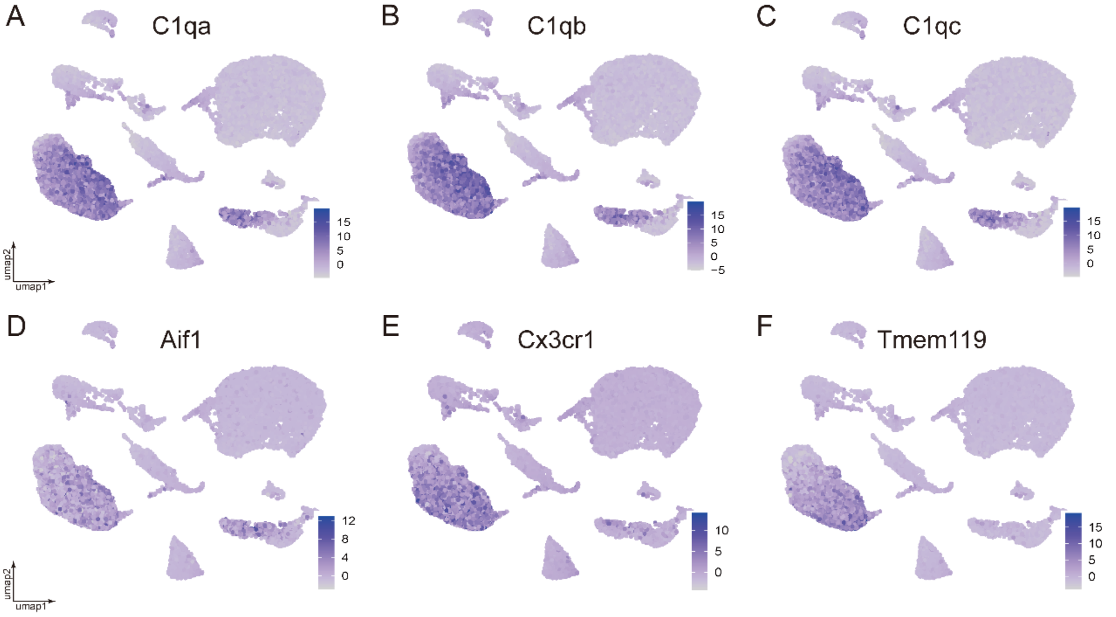

Figure S1. UMAP plots showing the expression distribution of a series microglial marker genes, including (A) *C1qa*, (B) *C1qb*, (C) *C1qc*, (D) *Aif1*, (E) *Cx3cr1*, (F) *Tmem119*. UMAP, Uniform Manifold Approximation and Projection;

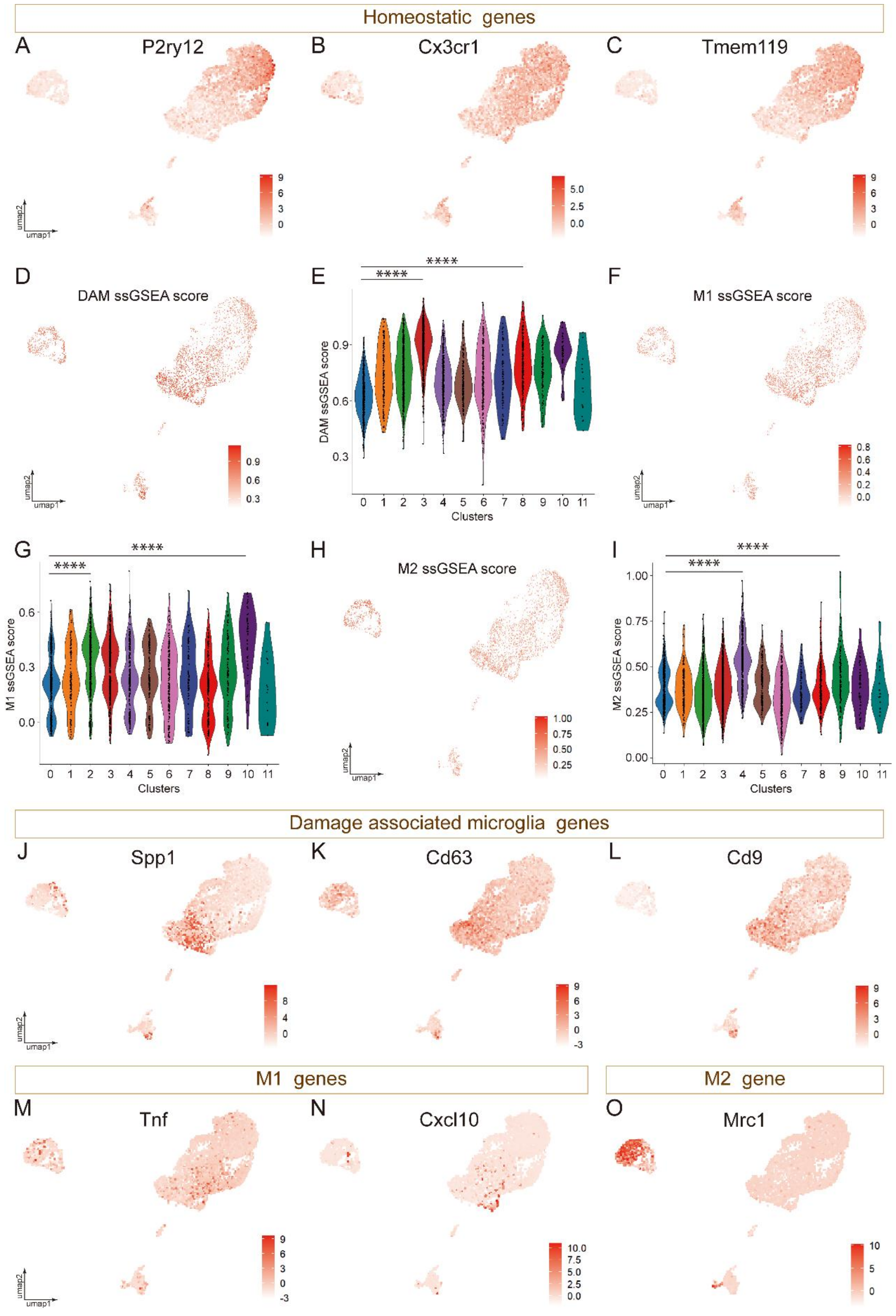

Figure S2. Identification of DAM, M1, M2 in microglia subclusters.

(A-C) UMAP plots showing the expression distribution of homeostatic genes in microglia subclusters, including (A) *P2ry12*, (B) *Cx3cr1*, (C) *Tmem119*.

(D) UMAP plot visualizing the DAM ssGSEA score distribution in each microglia subclusters.

(E) Violin diagram representing the DAM ssGSEA score level in each microglia subclusters.
(F) UMAP plot visualizing the M1 ssGSEA score distribution in each microglia subclusters.
(G) Violin diagram representing the M1 ssGSEA score level in each microglia subclusters.
(H) UMAP plot visualizing the M2 ssGSEA score distribution in each microglia subclusters.
(I) Violin diagram representing the M2 ssGSEA score level in each microglia subclusters.
(J-L) UMAP plots showing the expression distribution of DAM genes in microglia subclusters, including (J) *Spp1*, (K) *Cd63*, (L) *Cd9*.
(M-N) UMAP plots showing the expression distribution of M1 genes in microglia subclusters, including (M) *Tnf*, (N) *Cxcl10*.
(O) UMAP plots showing the expression distribution of M2 genes *Mrc1* in microglia subclusters,
Data are presented as mean ± SD. one-way ANOVA; **** $P < 0.0001$. UMAP, Uniform Manifold Approximation and Projection; ssGSEA, single-sample Gene Set Enrichment Analysis; DAM, Disease Associated Microglia; M1, M1 polarized microglia; M2, M2 polarized microglia;

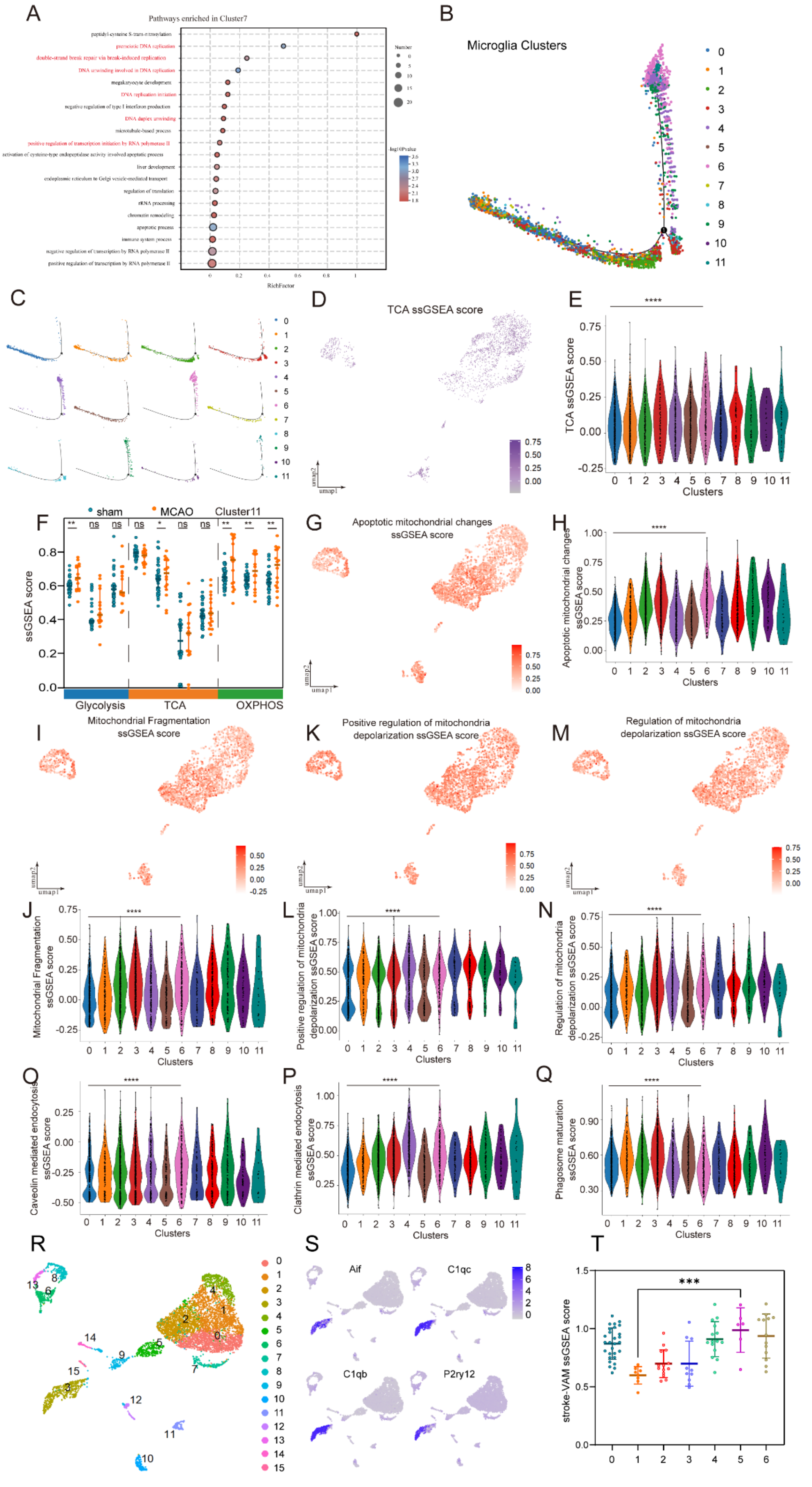
A
Pathways enriched in Cluster7
peptidyl-cysteine S-trans-nitrosylation
premeiotic DNA replication
double-strand break repair via break-induced replication
DNA unwinding involved in DNA replication
megakaryocyte development
DNA replication initiation
negative regulation of type I interferon production
DNA duplex unwinding
microtubule-based process
positive regulation of transcription initiation by RNA polymerase II
activation of cysteine-type endopeptidase activity involved apoptotic process
liver development
endoplasmic reticulum to Golgi vesicle-mediated transport
regulation of translation
rRNA processing
chromatin remodeling
apoptotic process
immune system process
negative regulation of transcription by RNA polymerase II
positive regulation of transcription by RNA polymerase II
Number
-lng10Pvalue
RichFactor
B
Microglia Clusters
C
D
TCA ssGSEA score
umap1
umap2
E
TCA ssGSEA score
Clusters
****
F
sham
MCAO
Cluster11
ssGSEA score
Glycolysis
TCA
OXPHOS
G
Apoptotic mitochondrial changes ssGSEA score
H
Apoptotic mitochondrial changes ssGSEA score
I
Mitochondrial Fragmentation ssGSEA score
K
Positive regulation of mitochondria depolarization ssGSEA score
M
Regulation of mitochondria depolarization ssGSEA score
J
Mitochondrial Fragmentation ssGSEA score
L
Positive regulation of mitochondria depolarization ssGSEA score
N
Regulation of mitochondria depolarization ssGSEA score
O
Caveolin mediated endocytosis ssGSEA score
P
Clathrin mediated endocytosis ssGSEA score
Q
Phagosome maturation ssGSEA score
R
S
Aif
C1qc
C1qb
P2ry12
T
stroke-VAM ssGSEA score
***

Figure S3. Explorating characteristics of VAM

(A) GO-BP terms enriched for microglial subcluster 7 under the threshold of P value < 0.05.

(B) Pseudotime analysis of microglia using Monocle based on tissue origin and Seurat determined clusters.

(C) Pseudotime analysis of different microglia sub-clusters using Monocle

(D) UMAP plot visualizing the TCA ssGSEA score distribution in each microglia subclusters.

(E) Violin diagram representing the TCA ssGSEA score level in each microglia subclusters.

(F) Scatter plot displaying ssGSEA score level of glycolysis, TCA, OXPHOS in microglia subcluster 11 between sham group and tMCAO group.

(G) UMAP plot visualizing the ssGSEA score distribution of apoptotic mitochondrial changes in each microglia subclusters.

(H) Violin diagram representing the ssGSEA score level of apoptotic mitochondrial changes in each microglia subclusters.

(I) UMAP plot visualizing the ssGSEA score distribution of mitochondrial fragmentation in each microglia subclusters.

(J) Violin diagram representing the ssGSEA score level of mitochondrial fragmentation in each microglia subclusters.

(K) UMAP plot visualizing the ssGSEA score distribution of positive regulation of mitochondria depolarization in each microglia subclusters.

(L) Violin diagram representing the ssGSEA score level of positive regulation of mitochondria depolarization in each microglia subclusters.

(M) UMAP plot visualizing the ssGSEA score distribution of regulation of mitochondria depolarization in each microglia subclusters.

(N) Violin diagram representing the ssGSEA score level of regulation of mitochondria depolarization in each microglia subclusters.

(O-Q) Violin diagram representing the ssGSEA score level of (O caveolin mediated endocytosis, (P) clathrin mediated endocytosis, (Q) phagosome maturation in each microglia subclusters.

(R) UMAP plot visualizing and unsupervised clustering of all cells colored by cell types in GSE233812

(S) UMAP plots showing the expression distribution of a series microglial marker genes (*Aif1, C1qc, C1qb, P2ry12*).

(T) Scatter plot displaying ssGSEA score level of stroke-VAM in microglia clusters.

Data are presented as mean ± SD. unpaired *t*-test; one-way ANOVA; ns, not significant; * $P < 0.05$; ** $P < 0.01$; *** $P < 0.001$; **** $P < 0.0001$. UMAP, Uniform Manifold Approximation and Projection; GO-BP, Gene Ontology: Biological Process; ssGSEA, single-sample Gene Set Enrichment Analysis; TCA, Tricarboxylic Acid Cycle; OXPHOS, Oxidative Phosphorylation; MCAO, Middle Cerebral Artery Occlusion.

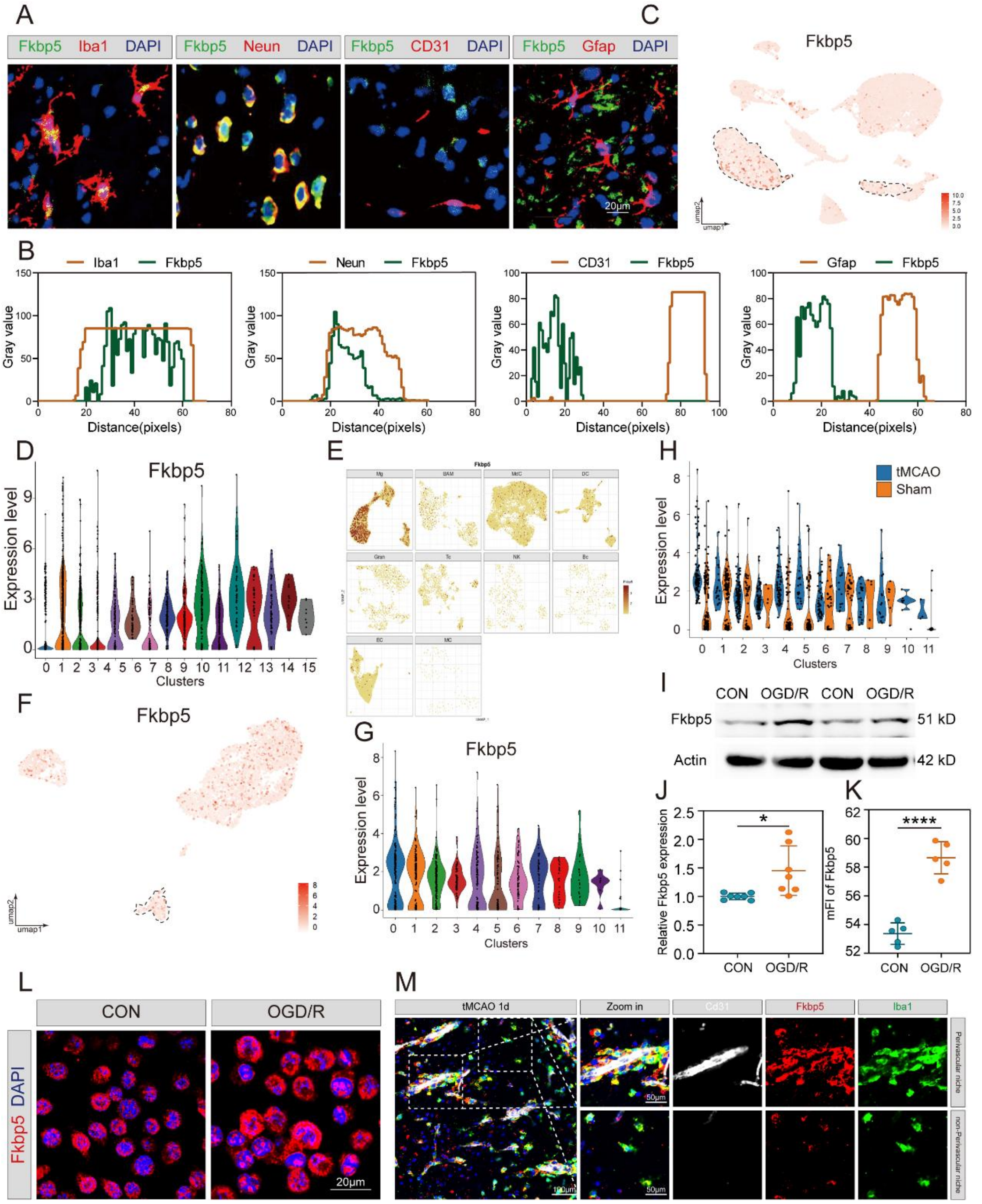

Figure S4. Identifying Fkbp5 in scRNA-seq data

(A-B) Representative photographs (A) and colocalization pattern analysis of immunofluorescent Fkbp5 with Iba1, Neun, CD31, or Gfap from brain sections subjected tMCAO.

(C) UMAP plot visualizing the distribution of Fkbp5 expression in all cell types analyzed from GSE 174574.

(D) Violin diagram representing the Fkbp5 expression in each cell types analyzed from GSE 174574.

(E) UMAP plot visualizing the distribution of Fkbp5 expression in all cell types analyzed from GSE 225948.

(F) UMAP plot visualizing the distribution of Fkbp5 expression in microglial subclusters.

(G) Violin diagram representing the Fkbp5 expression in microglial subclusters.

(H) Violin diagram showing the Fkbp5 expression of microglial subclusters in sham group and

tMCAO group.

(I-J) Representative immunoblots (I) and quantitative data (J) of Fkbp5 protein content in BV2 cells under normal condition (n = 7) and OGD/R condition (n = 7).

(K-L) Representative micrograph (L) and quantitative data of Fkbp5 immunofluorescence in BV2 cells under normal condition (n = 5) and OGD/R condition (n = 5).

(M) Representative pictures of immunofluorescent Fkbp5, Iba1, and Cd31 showing the expression of microglial Fkbp5 in perivascular niche versus non-perivascular area in peri-infarct region at 1-day post-stroke. n = 8 for each group.

Data are presented as mean ± SD. one-way ANOVA; * $P < 0.05$; ** $P < 0.01$; *** $P < 0.001$; **** $P < 0.0001$. UMAP, Uniform Manifold Approximation and Projection; BAM, Border-associated macrophages; Bc, B cells; DC, dendritic cells; EC, endothelial cells; Gran, granulocytes; MC, mural cells; MdC, monocyte-derived cells; Mg, microglia; NK, NK cells; Tc, T cells; tMCAO, transient Middle Cerebral Artery Occlusion. OGD/R, Oxygen Glucose Deprivation/Reperfusion; DAPI, 4′,6-diamidino-2-phenylindole.

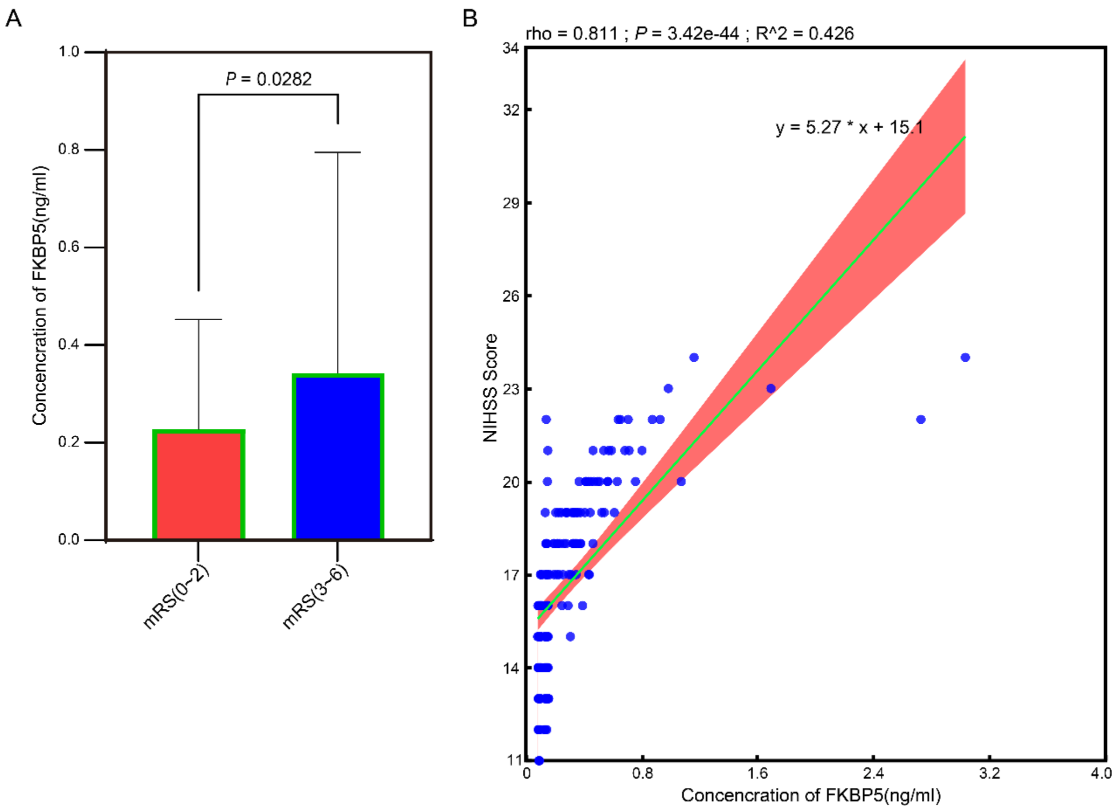

Fig S5. Serum FKBP5 concentrations positively associated with poor prognosis in patients with acute ischemic stroke

(A) Histogram showing serum FKBP5 concentrations in AIS patients with mRS(0-2) and mRS(3-6). n = 92 for each group.

(B) Analysis graph of the correlation between serum FKBP5 concentration and NIHSS score in AIS patients.

Data are presented as mean ± SD, median (IQR), or frequencies (%) as appropriate. Given the skewed distribution, serum FKBP5 concentrations were natural-log-transformed for parametric

analyses. The association between log-transformed FKBP5 and baseline NIHSS score was assessed using a linear mixed-effects model, with matched-pair ID as a random intercept. The association with 90-day functional outcome (good [mRS 0-2] vs. poor [mRS 3-6]) was evaluated using conditional logistic regression, stratified by matched-pair ID. Results are reported as odds ratios (OR) with 95% confidence intervals (CIs). mRS, Modified Rankin Scale; NIHSS, National Institutes of Health Stroke Scale; AIS, acute ischemic stroke.

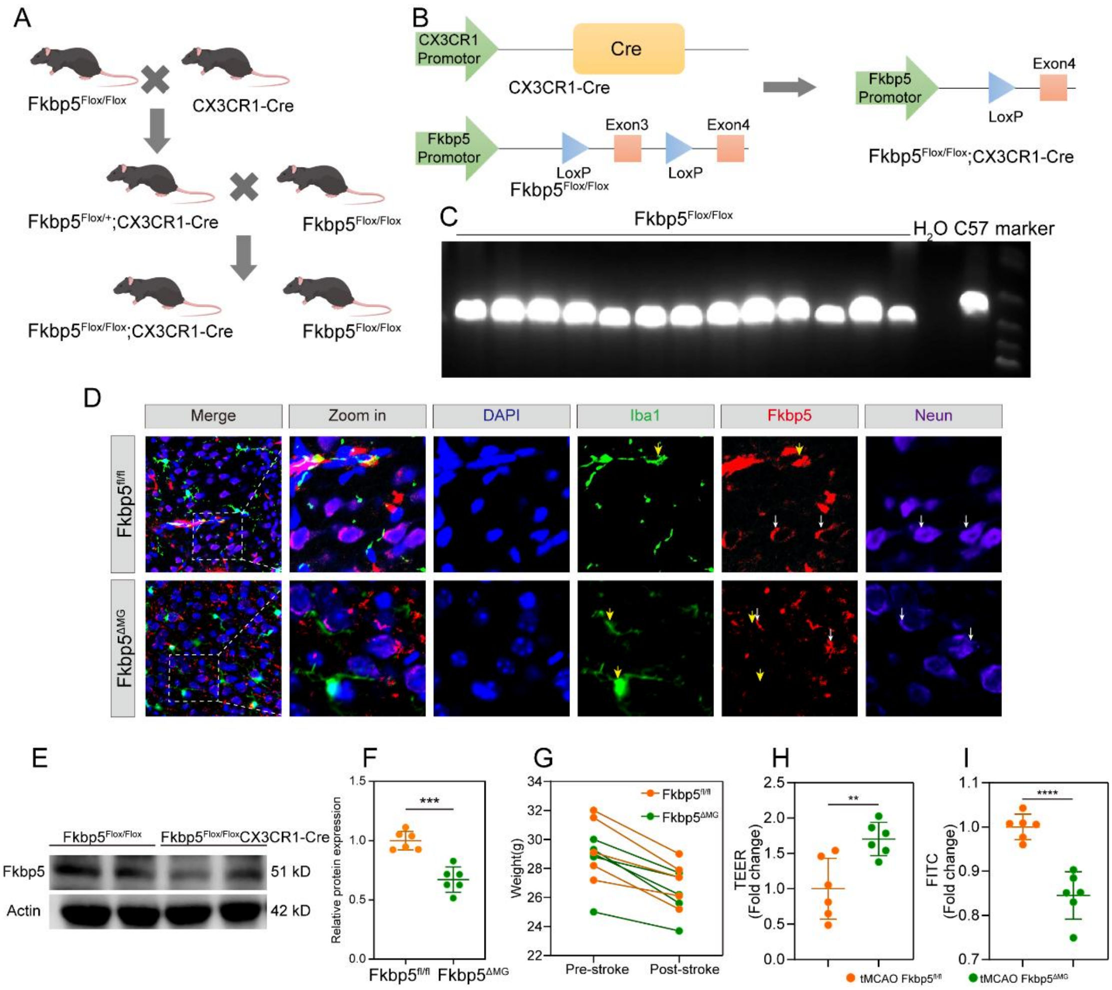

Figure S6. Establishing and identifying microglial conditional knockout of Fkbp5 in mice (Fkbp5$^{\Delta MG}$ mice)

(A) Strategy of construction of Fkbp5$^{\Delta MG}$ mice.

(B) Schematic view of establishing Fkbp5$^{\Delta MG}$ mice: insertion of Loxp site on both ends of exon3.

(C) Genotyping of Fkbp5$^{\Delta MG}$ mice by polymerase chain reaction (PCR) analysis and nucleotide gel electrophoresis

(D) Representative micrographs of Fkbp5, Iba1, and Neun in cerebral cortex from Fkbp5$^{fl/fl}$ mice and Fkbp5$^{\Delta MG}$ mice. Yellow arrows indicate the regions where the Fkbp5 channel corresponds to the Iba1 channel. White arrows indicate the regions where the Fkbp5 channel corresponds to the Neun channel.

(E-F) Representative image (F) and quantitative data (G) of Fkbp5 in cerebral cortex from Fkbp5$^{fl/fl}$ mice (n = 6) and Fkbp5$^{\Delta MG}$ (n = 6) mice.

(G) Line graph exhibiting the weight change between pre-stroke and post-stroke from Fkbp5$^{fl/fl}$ mice and Fkbp5$^{\Delta MG}$ mice. n = 5 per group.

(H) TEER value displaying the endothelial cells monolayer permeability co-cultured with Fkbp5-deficient microglia or control microglia. n = 6 per group.

(I) Leakage of FITC-conjugated dextran in the endothelial cells monolayer showed endothelial cells integrity, co-cultured with Fkbp5-deficient microglia or control microglia. n = 6 per group.

Data are presented as mean ± SD. unpaired *t*-test; paired *t*-test; ns, not significant; *** $P < 0.001$. Fkbp5$^{fl/fl}$, *Fkbp5*$^{flox/flox}$ mice without Fkbp5 conditional deletion in microglia; Fkbp5$^{\Delta MG}$, *Fkbp5*$^{flox/flox}$ and *Cx3cr1*^{Cre} mice with Fkbp5 conditional deletion in microglia. TEER, Transendothelial electrical resistance; FITC, fluorescein isothiocyanate. DAPI, 4′,6-diamidino-2-phenylindole.

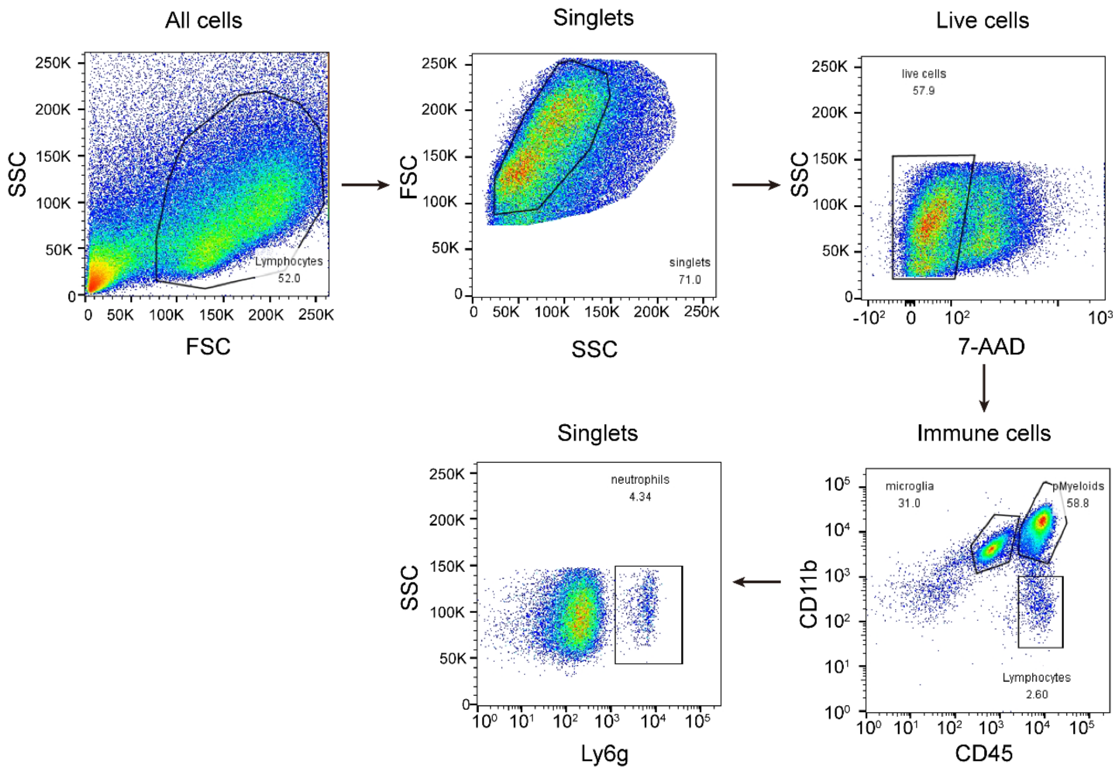

Figure S7. Representative gating strategies of pMyeloids, microglia, lymphocytes, and neutrophils. FSC, Forward Scatter; SSC, Side Scatter; 7-AAD, 7-Aminoactinomycin D.

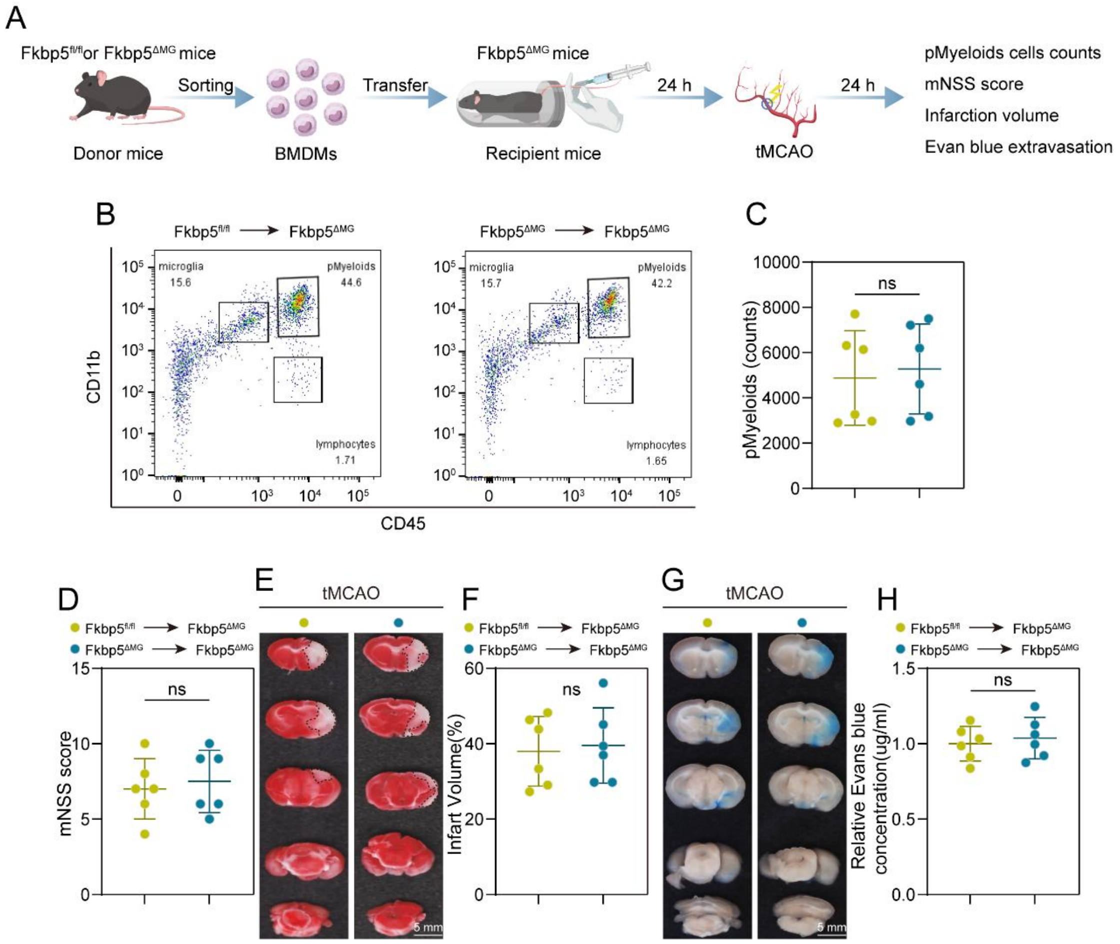

Fig S8. Fkbp5 deficiency in peripherally derived macrophages does not influence stroke outcome.

(A) The flowchart illustrates the experimental design for bone marrow transplantation and tMCAO.

(**B-C**) Flow cytometry demonstrates representative images and statistical results of CD11b$^+$CD45$^+$ (myeloid-derived immune cells) proportions in brain tissue 1 day after tMCAO. *n* = 6 for each group.

(**D**) Neurological function evaluation by mNSS score system of *Fkbp5*$^{flox/flox}$→*Fkbp5*$^{\Delta MG}$ mice and *Fkbp5*$^{\Delta MG}$→*Fkbp5*$^{\Delta MG}$ mice at 1 day after tMCAO. *n* = 6 per group.

(E-F) Representative pictures (**E**) and quantitative data (**F**) of infarct volume in *Fkbp5*$^{flox/flox}$→*Fkbp5*$^{\Delta MG}$ mice and *Fkbp5*$^{\Delta MG}$→*Fkbp5*$^{\Delta MG}$ mice at 1 day after tMCAO measured by TTC staining. *n* = 6 for each group.

(**G-H**) Representative image (**G**) and quantitative data (**H**) of Evans blue leakage in tMCAO *Fkbp5*$^{flox/flox}$→*Fkbp5*$^{\Delta MG}$ mice and tMCAO *Fkbp5*$^{\Delta MG}$→*Fkbp5*$^{\Delta MG}$ mice. *n* = 6 for each group. Data are presented as means ± SD. One-way ANOVA with Bonferroni post-tests. ns indicates no significant difference. Fkbp5$^{fl/fl}$, *Fkbp5*$^{flox/flox}$ mice without Fkbp5 conditional deletion in microglia; Fkbp5$^{\Delta MG}$, *Fkbp5*$^{flox/flox}$ and *Cx3cr1*^{Cre} mice with Fkbp5 conditional deletion in microglia; BMDMs, bone marrow-derived myeloid cells; tMCAO, transient Middle Cerebral Artery Occlusion.

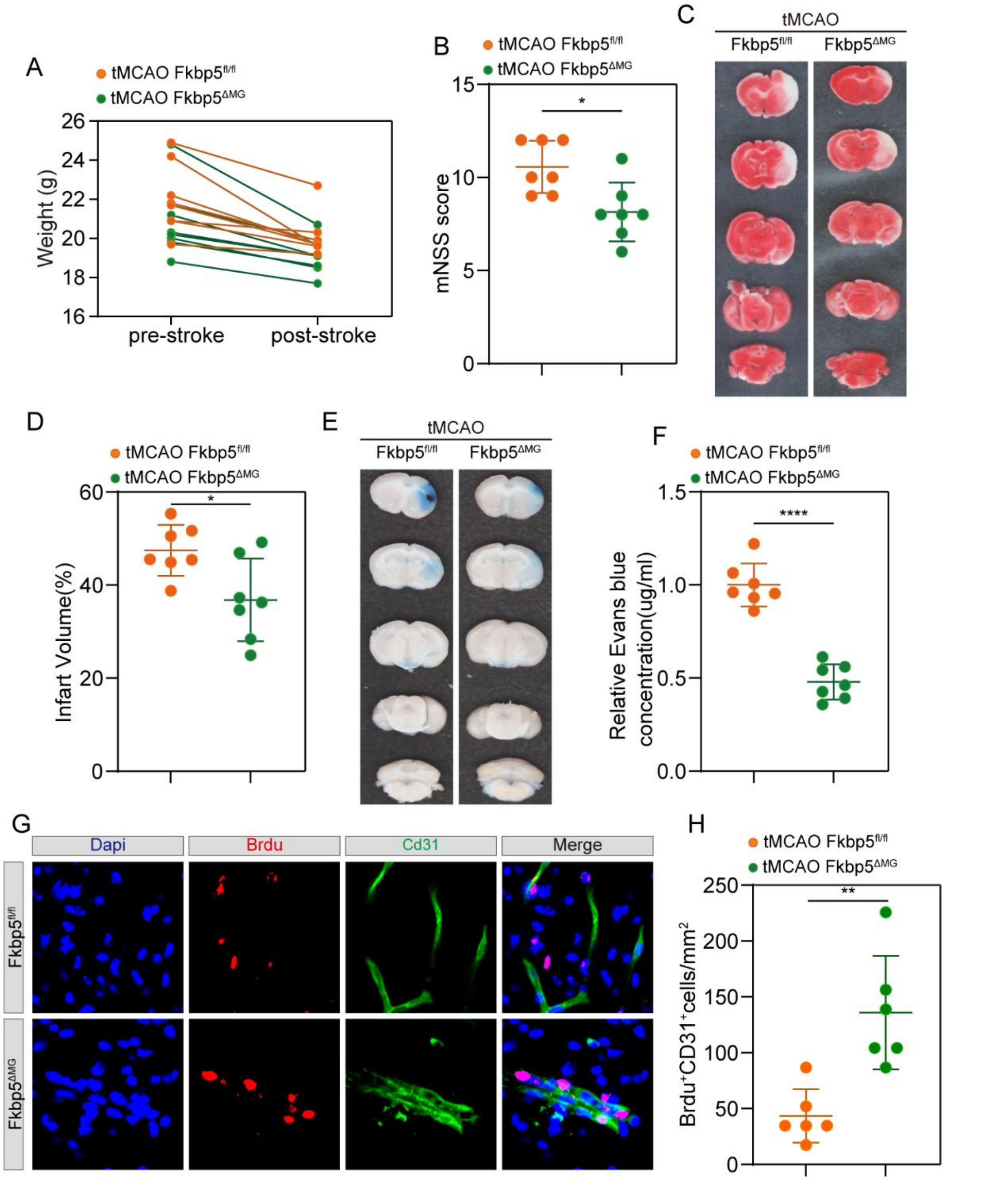

Figure S9. Inhibiting stroke-VAM by Fkbp5 deletion in microglia attenuates BBB disruption and inflammation after post-stroke in female mice.

(A) Body weight change was measured after tMCAO.

(B) Neurobehavior functions were evaluated after tMCAO by mNSS score.

(C-D) Representative pictures (C) of 2,3,5-triphenyl tetrazolium chloride (TTC)-stained brain sections per 2-mm slices and quantitative data (D) of infarct volume after tMCAO.

(E-F) Representative images (E) of mice brain stained with Evans Blue 24 hours after surgery and analysis (F) of Evans Blue concentration in cerebral parenchyma.

(G-H) Representative photographs and quantitative data of BrdU/CD31 immunofluorescence in the penumbra.

Data are presented as means ± SD. One-way ANOVA with Bonferroni post-tests. *$P<0.05$, **$P<0.01$, ***$P<0.001$, **** $P<<0.0001$. n=6 or 7mice/group. tMCAO, transient middle cerebral

artery occlusion; Fkbp5$^{fl/fl}$, *Fkbp5*$^{flox/flox}$ mice without Fkbp5 conditional deletion in microglia; Fkbp5$^{\Delta MG}$, *Fkbp5*$^{flox/flox}$ and *Cx3cr1*^{Cre} mice with Fkbp5 conditional deletion in microglia; DAPI, 4′,6-diamidino-2-phenylindole.

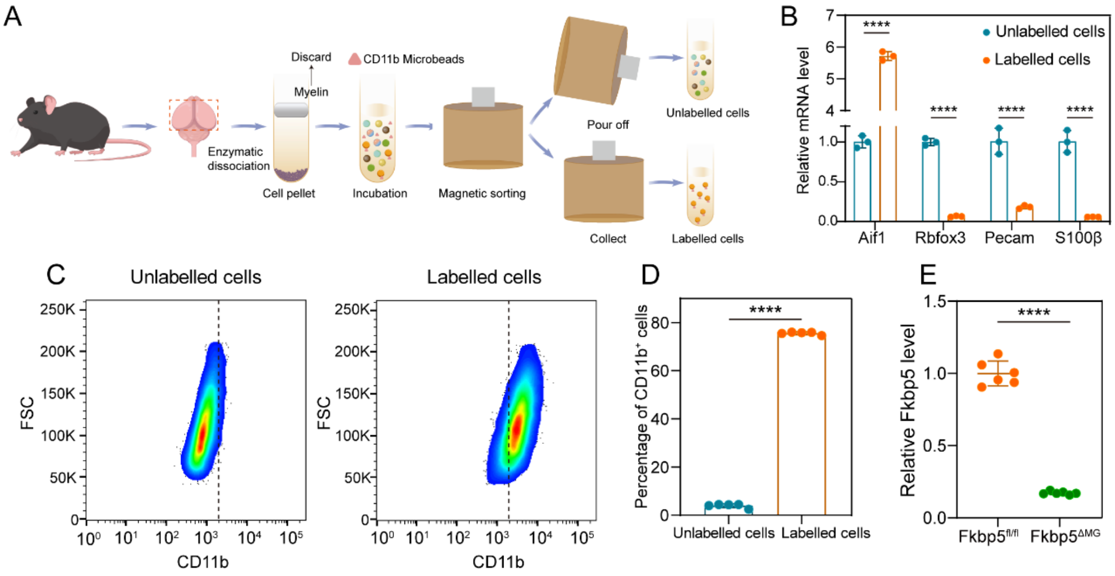

Fig S10, isolation of CD11b^{+} cells from brain.

(A) Workflow of isolation of CD11b^{+} cells from cerebral in mice.

(B) qPCR identifying the gene expression of microglial marker (Iba1), Neuronal marker (Rbfox3), endothelial cells marker (Pecam1), and astrocytes marker (S100β) in CD11b^{+} beads labelled cells and CD11b^{+} beads unlabeled cells from (H).(n = 3)

(C-D) Flow cytometry validating the CD11b expression in CD11b^{+} beads labelled cells and CD11b^{+} beads unlabeled cells from (A). (n=5)

(E) qPCR validation the Fkbp5 mRNA level of CD11b^{+} beads from Fkbp5$^{fl/fl}$ mice and Fkbp5$^{\Delta MG}$ mice. n = 6 per group.

Data are presented as mean ± SD. unpaired *t*-test; **** $P < 0.0001$. Fkbp5$^{fl/fl}$, *Fkbp5*$^{flox/flox}$ mice without Fkbp5 conditional deletion in microglia; Fkbp5$^{\Delta MG}$, *Fkbp5*$^{flox/flox}$ and *Cx3cr1*^{Cre} mice with Fkbp5 conditional deletion in microglia; FSC, Forward Scatter.

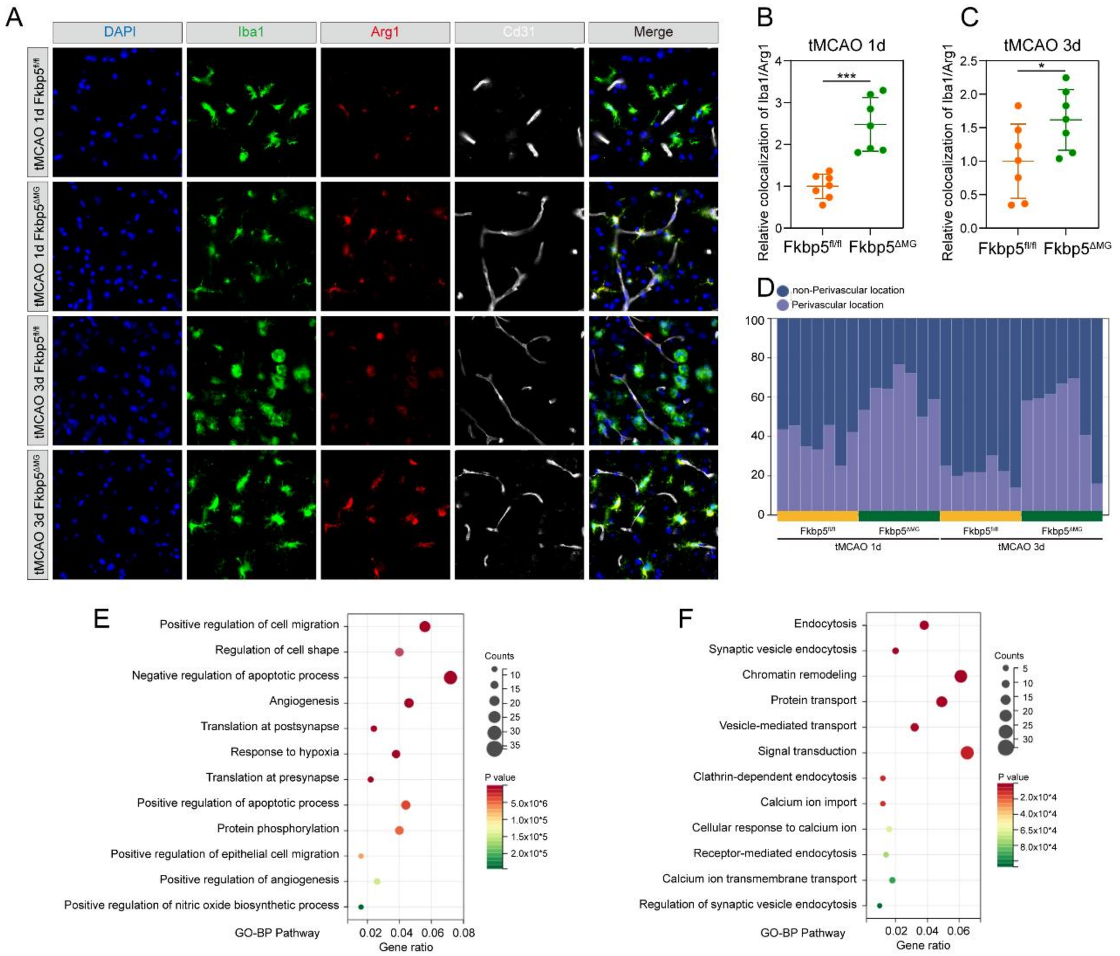

Fig S11. Spatiotemporal and functional characteristics of the elevated M2 markers in Fkbp5-deficient stroke-VAM.

(A) Representative micrographs of brain sections immunostaining Iba1 (green), Arg1 (red), CD31 (white) in peri-infarct area of Fkbp5$^{fl/fl}$ mice and Fkbp5$^{\Delta MG}$ mice at 1 day and 3 days post-stroke. Nuclei were stained with DAPI (blue).

(B) Colocalization quantitative data (N) of brain sections immunostaining Iba1 (green) and Arg1 (red) in peri-infarct area of Fkbp5$^{fl/fl}$ mice (n = 7) and Fkbp5$^{\Delta MG}$ mice (n = 7) at 1 day post-stroke.

(C) Colocalization quantitative data (N) of brain sections immunostaining Iba1 (green) and Arg1 (red) in peri-infarct area of Fkbp5$^{fl/fl}$ mice (n = 7) and Fkbp5$^{\Delta MG}$ mice (n = 7) at 3 days post-stroke.

(D) Stacked histogram showing the localization relationship of Arg1^{+} microglia with blood vessels in Fkbp5$^{fl/fl}$ mice and Fkbp5$^{\Delta MG}$ mice at 1 day and 3 days post-stroke. n = 7 for each group.

(E) GO-BP terms enriched for up regulated genes of Fkbp5-deficient stroke-VAM compared to canonical M2 under the threshold of *P* value < 0.05.

(F) GO-BP terms enriched for down regulated genes of Fkbp5-deficient stroke-VAM compared to canonical M2 under the threshold of *P* value < 0.05.

Data are presented as mean ± SD; unpaired *t*-test; one-way ANOVA; ns, not significant; * $P < 0.05$; ** $P < 0.01$; *** $P < 0.001$; **** $P < 0.0001$. Fkbp5$^{fl/fl}$, *Fkbp5*$^{flox/flox}$ mice without Fkbp5 conditional deletion in microglia; Fkbp5$^{\Delta MG}$, *Fkbp5*$^{flox/flox}$ and *Cx3cr1*^{Cre} mice with Fkbp5 conditional deletion in microglia; GO-BP, Gene Ontology: Biological Process; tMCAO, transient Middle Cerebral Artery Occlusion; DAPI, 4′,6-diamidino-2-phenylindole.

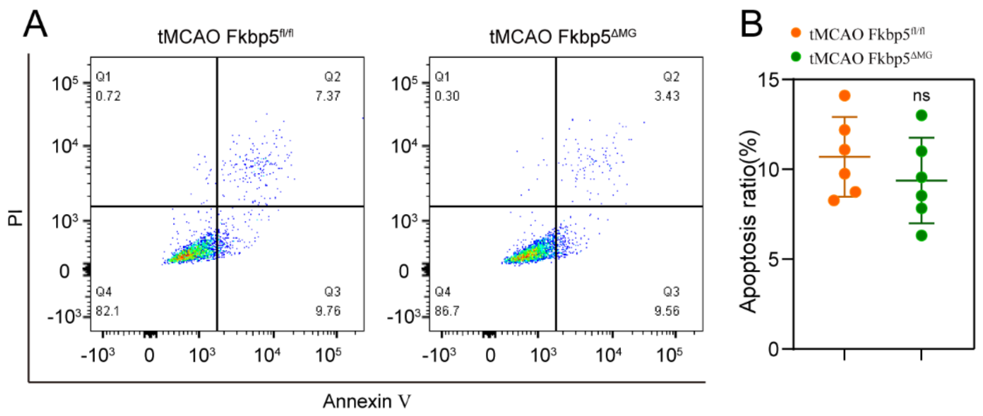

Figure S12. Fkbp5 deficiency does not affect apoptosis in microglia.

(A-B) Representative images (A) of Annexin V/PI staining on both *Fkbp5*$^{flox/flox}$ and Fkbp5-deficient microglial cells after surgery and quantitative data (B) of apoptosis ratio in microglial cells. n = 6 per group.

Data are presented as means ± SD. unpaired *t*-test; ns indicates no significant difference. Fkbp5$^{fl/fl}$, *Fkbp5*$^{flox/flox}$ mice without Fkbp5 conditional deletion in microglia; Fkbp5$^{\Delta MG}$, *Fkbp5*$^{flox/flox}$ and *Cx3cr1*^{Cre} mice with Fkbp5 conditional deletion in microglia; tMCAO, transient middle cerebral artery occlusion.

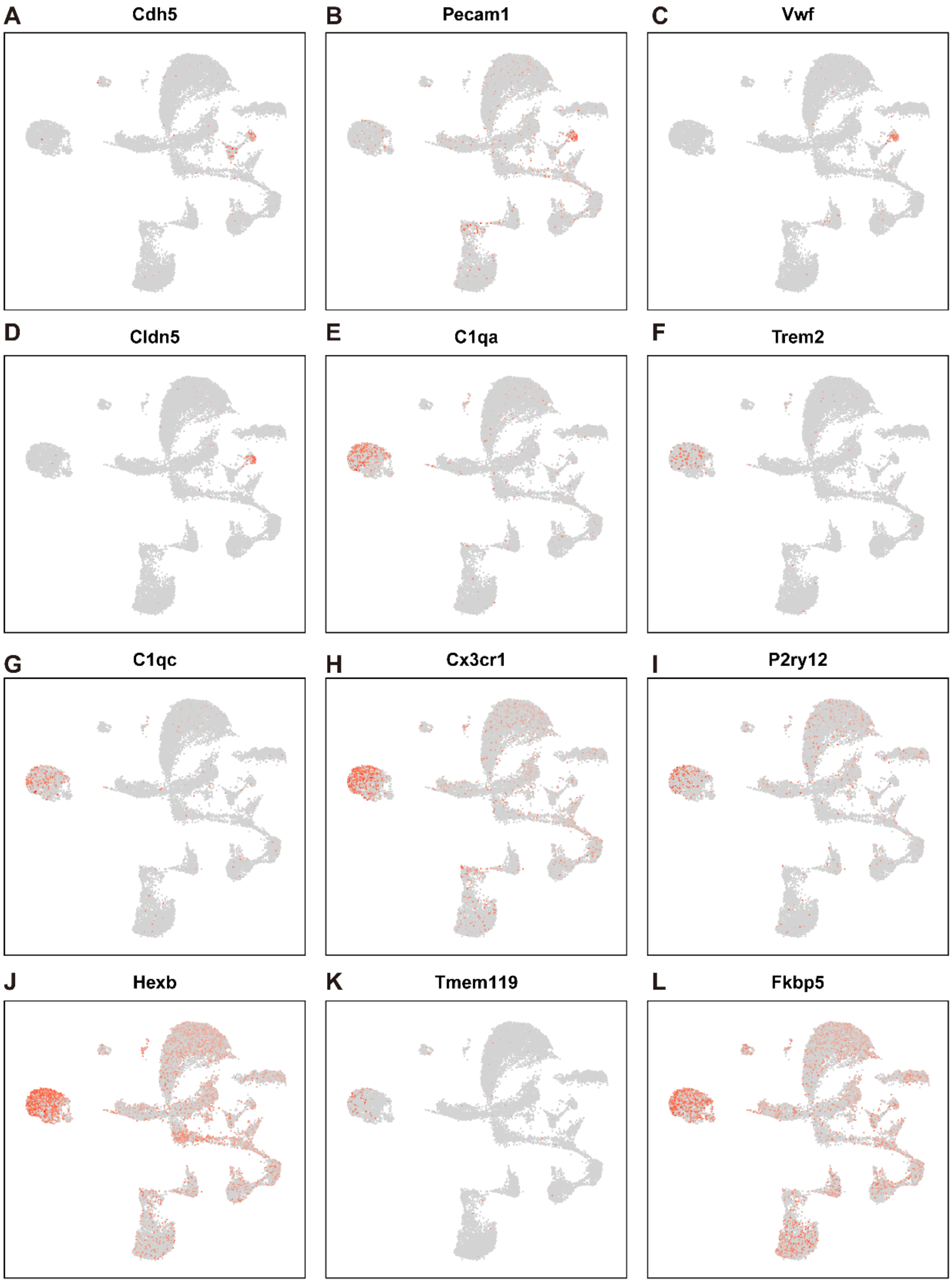

Figure S13. Identifying endothelial cells and microglia in sn-RNA seq.

(A-D) UMAP plots showing the expression distribution of a series endothelial cells marker genes, including (A) *Cdh5*, (B) *Pecam1*, (C) *Vwf*, (D) *Cldn5*.

(E-K) UMAP plots showing the expression distribution of a series microglial marker genes, including (E) *C1qa*, (F) *Trem2*, (G) *C1qc*, (H) *Cx3cr1*, (I) *P2ry12*, (J) *Hexb*, (K) *Tmem119*.

(L) UMAP plots showing the expression distribution of *Fkbp5*.

UMAP, Uniform Manifold Approximation and Projection

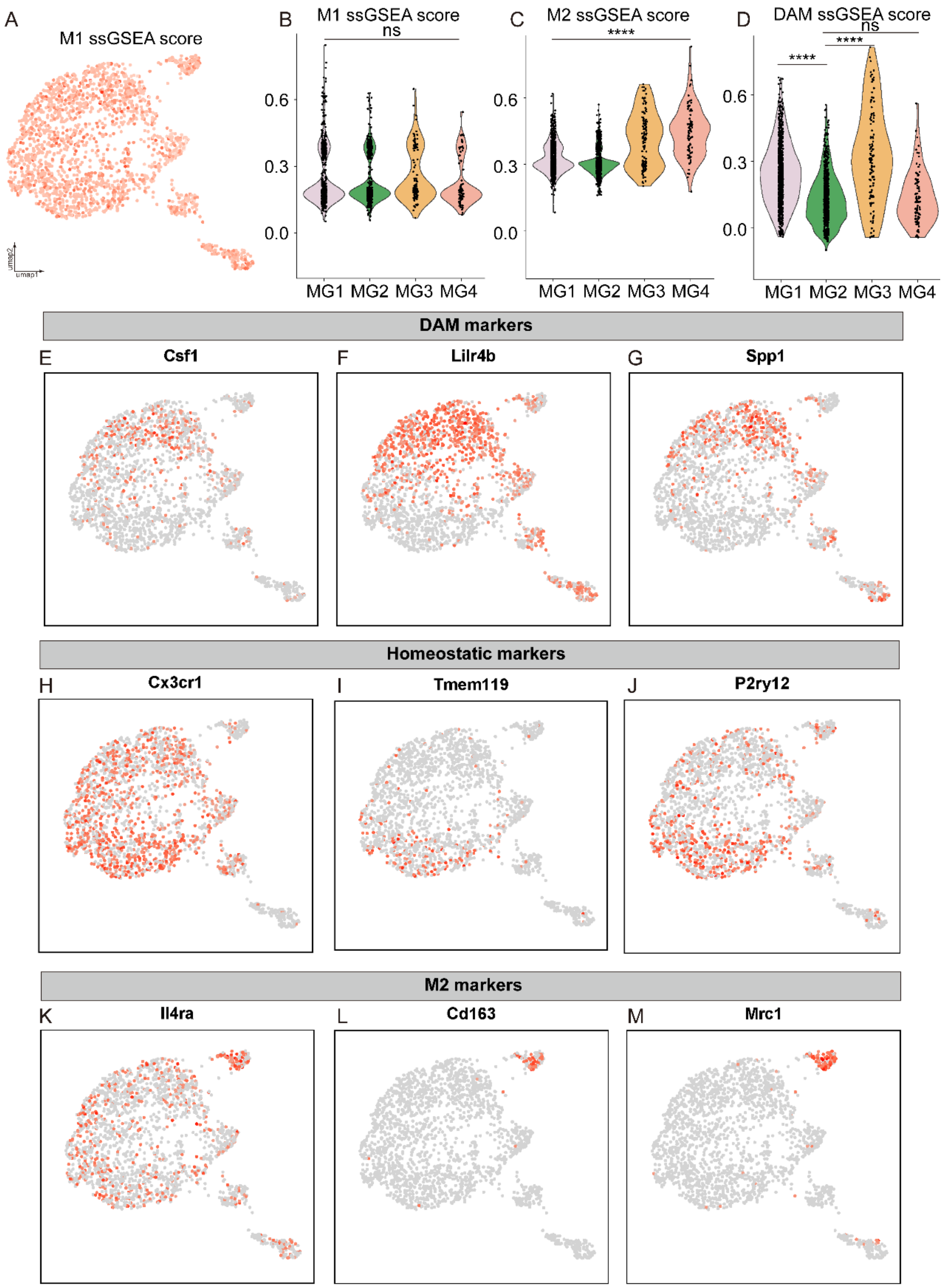

Figure S14. Identifying DAM, M1, and M2 clusters in microglia from snRNA seq.

(A) UMAP plot showing the expression distribution of M1 ssGSEA score in each microglial subclusters from sn-RNA seq.

(B-D) Violin graphs representing the ssGSEA score level of (B) M1 ssGSEA score, (C) M2 ssGSEA score, (D) DAM ssGSEA score in each microglia subclusters.

(E-G) UMAP plots showing the expression distribution of DAM genes in microglia subclusters, including (D) *Csf1*, (E) *Lilr4b*, (F) *Spp1*.

(H-J) UMAP plots showing the expression distribution of homeostatic genes in microglia subclusters, including (H) *Cx3cr1*, (I) *Tmem119*, (J) *P2ry12*.

(K-M) UMAP plots showing the expression distribution of M2 genes in microglia subclusters, including (K) *Il4ra*, (L) *Cd163*, (M) *Mrc1*.

Data are presented as mean ± SD. one-way ANOVA; ns, not significant; **** $P < 0.0001$. UMAP, Uniform Manifold Approximation and Projection; ssGSEA, single-sample Gene Set Enrichment Analysis; DAM, Disease Associated Microglia; M1, M1 polarized microglia; M2, M2 polarized microglia.

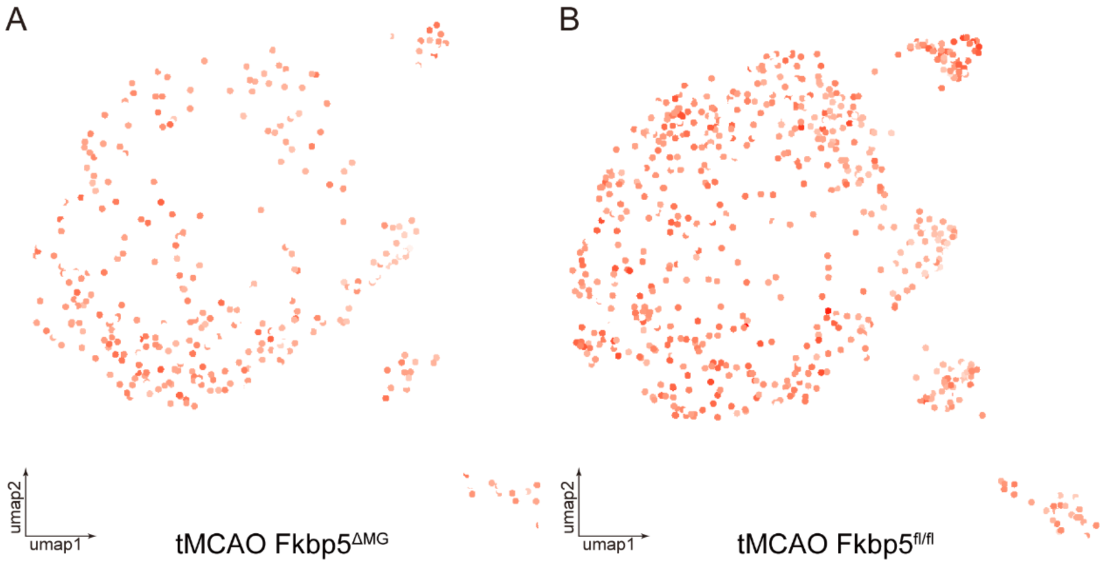

Fig S15, Deletion of Fkbp5 in microglia identified by scRNA-seq data.

(A-B) UMAP plot showing the differently expressed Fkbp5 in tMCAO Fkbp5$^{\Delta MG}$ mice (A) and tMCAO Fkbp5$^{fl/fl}$ mice (B). UMAP, Uniform Manifold Approximation and Projection; tMCAO, transient Middle Cerebral Artery Occlusion; Fkbp5$^{fl/fl}$, *Fkbp5*$^{flox/flox}$ mice without Fkbp5 conditional deletion in microglia; Fkbp5$^{\Delta MG}$, *Fkbp5*$^{flox/flox}$ and *Cx3cr1*^{Cre} mice with Fkbp5 conditional deletion in microglia;

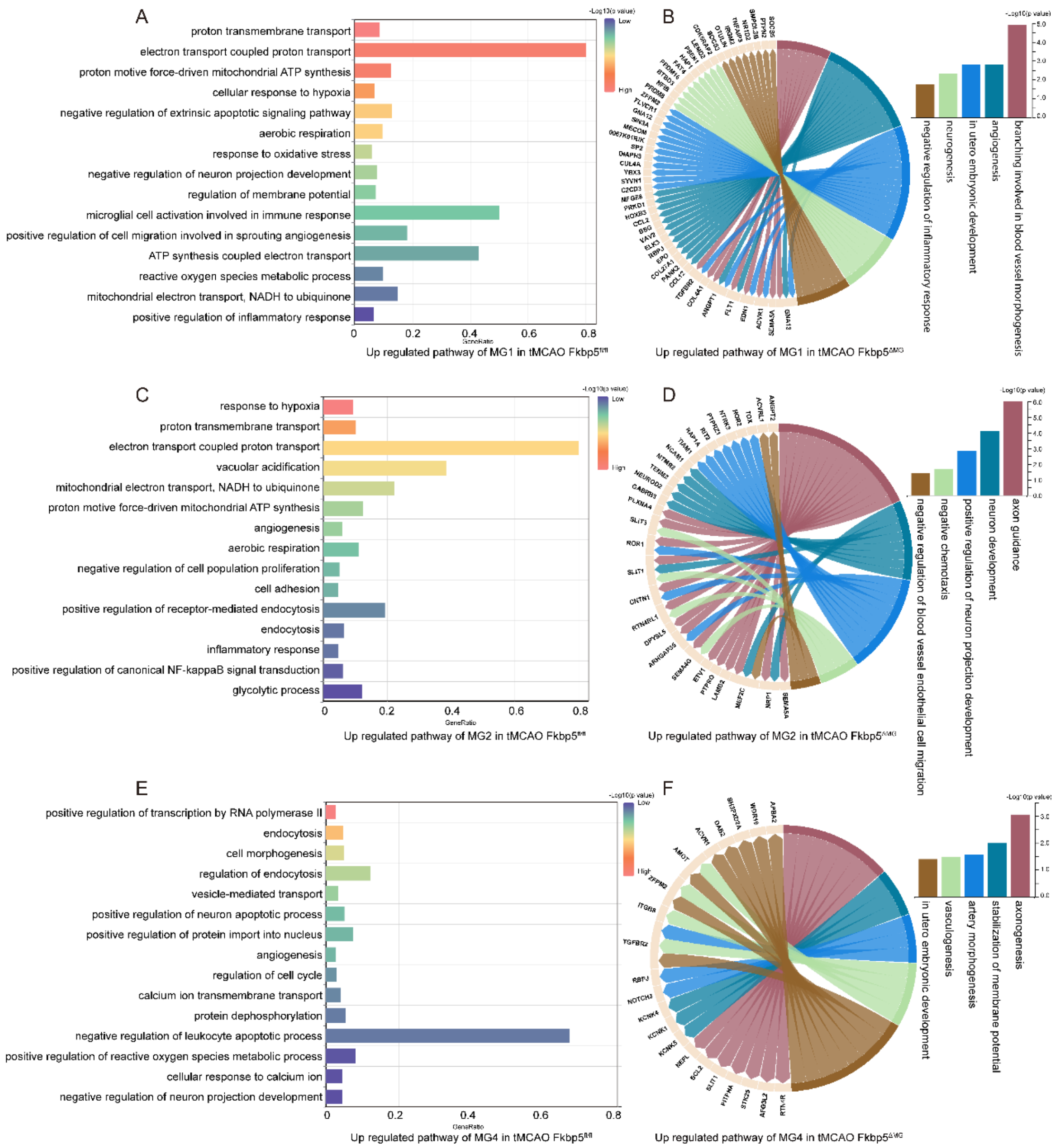

Figure S16, Exploring the role of Fkbp5 deficiency in microglia after ischemic stroke.

(A) GO-BP terms enriched of up regulated DEGs of MG1 in Fkbp5$^{fl/fl}$ mice after tMCAO under the threshold of P value < 0.05.

(B) GO-BP terms enriched of up regulated DEGs of MG1 in Fkbp5$^{\Delta MG}$ mice after tMCAO under the threshold of P value < 0.05.

(C) GO-BP terms enriched of up regulated DEGs of MG2 in Fkbp5$^{fl/fl}$ mice after tMCAO under the threshold of P value < 0.05.

(D) GO-BP terms enriched of up regulated DEGs of MG2 in Fkbp5$^{\Delta MG}$ mice after tMCAO under the threshold of P value < 0.05.

(E) GO-BP terms enriched of up regulated DEGs of MG4 in Fkbp5$^{fl/fl}$ mice after tMCAO under the threshold of P value < 0.05.

(F) GO-BP terms enriched of up regulated DEGs of MG4 in Fkbp5$^{\Delta MG}$ mice after tMCAO under the threshold of P value < 0.05.

GO-BP, Gene Ontology: Biological Process; tMCAO, transient Middle Cerebral Artery Occlusion; Fkbp5$^{fl/fl}$, *Fkbp5*$^{flox/flox}$ mice without Fkbp5 conditional deletion in microglia; Fkbp5$^{\Delta MG}$, *Fkbp5*$^{flox/flox}$ and *Cx3cr1*^{Cre} mice with Fkbp5 conditional deletion in microglia; DEGs, Differentially Expressed Genes.

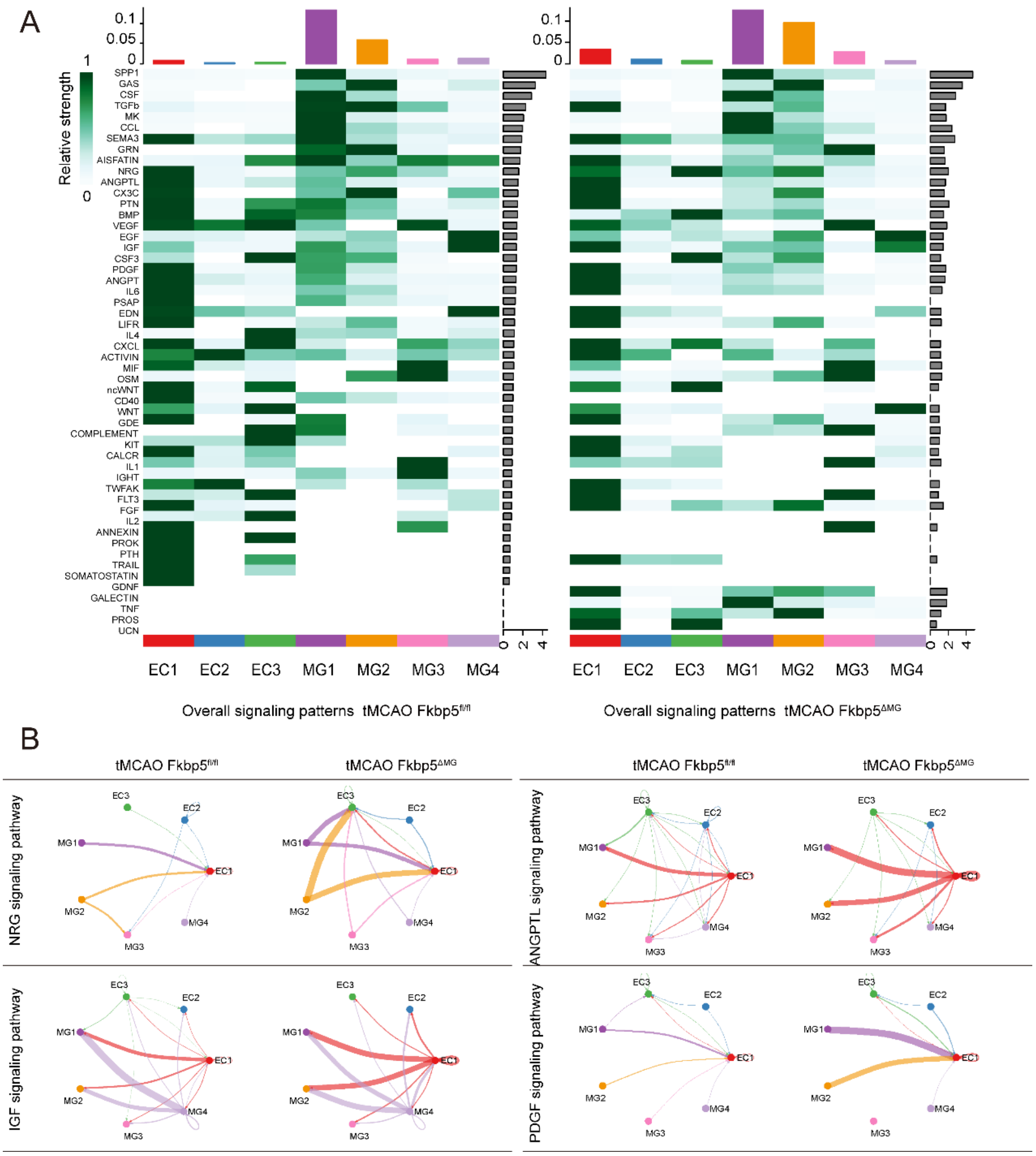

Fig S17, Cellchat interaction between microglia and endothelial cells in Fkbp5$^{\Delta MG}$ group and Fkbp5$^{fl/fl}$ group after ischemic stroke.

(A) ligands-receptors pair communication between microglial cells and endothelial cells in tMCAO Fkbp5$^{\Delta MG}$ mice and tMCAO Fkbp5$^{fl/fl}$ mice.

(B) Signaling pathways communication between microglial cells and endothelial cells, including NRG signaling pathway, IGF signaling pathway, ANGPTL signaling pathay, and PDGF signaling pathway.

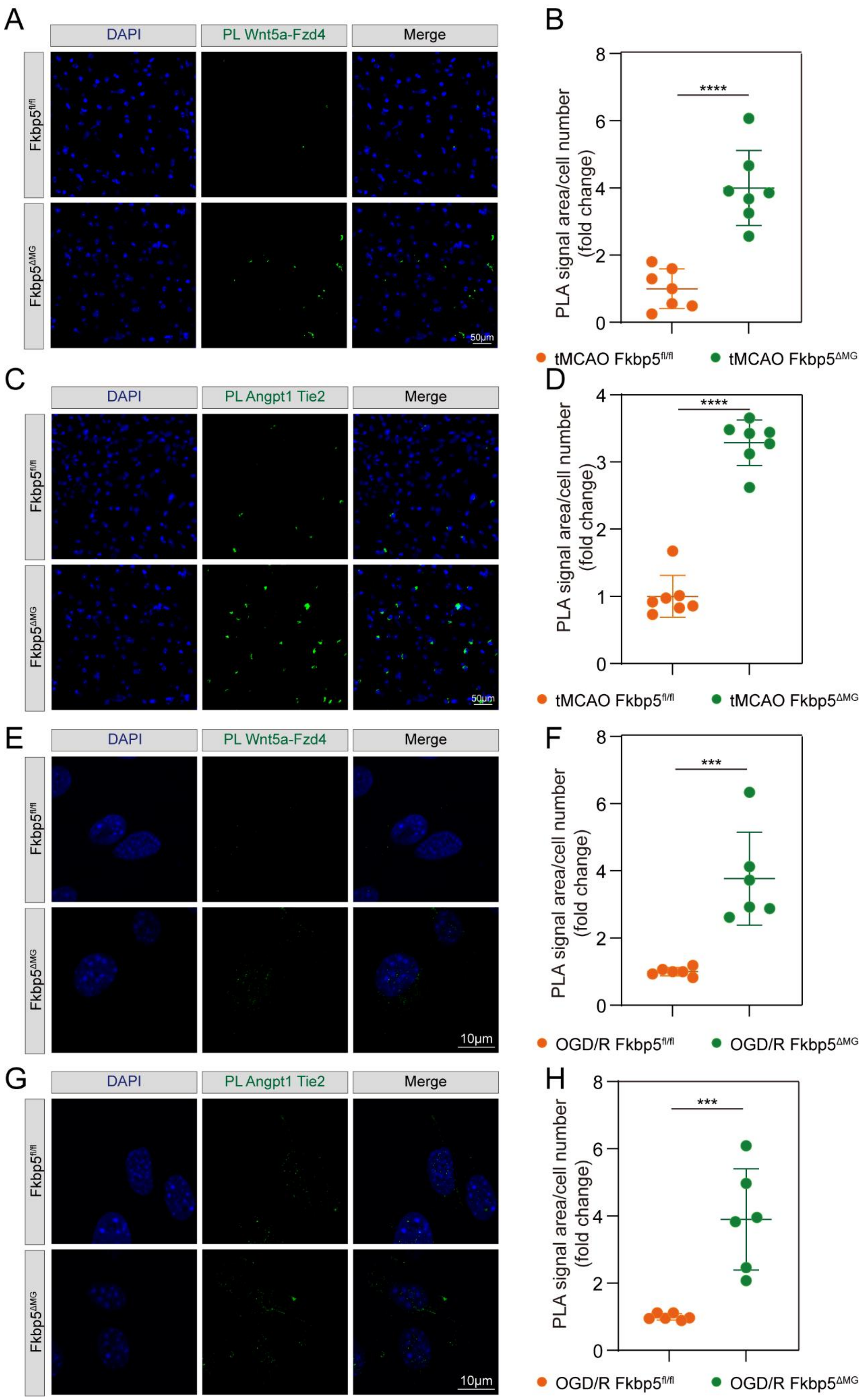
A
DAPI
PL Wnt5a-Fzd4
Merge
Fkbp5fl/fl
Fkbp5ΔMG
50μm
B
PLA signal area/cell number (fold change)
****
tMCAO Fkbp5fl/fl
tMCAO Fkbp5ΔMG
C
DAPI
PL Angpt1 Tie2
Merge
Fkbp5fl/fl
Fkbp5ΔMG
50μm
D
PLA signal area/cell number (fold change)
****
tMCAO Fkbp5fl/fl
tMCAO Fkbp5ΔMG
E
DAPI
PL Wnt5a-Fzd4
Merge
Fkbp5fl/fl
Fkbp5ΔMG
10μm
F
PLA signal area/cell number (fold change)
***
OGD/R Fkbp5fl/fl
OGD/R Fkbp5ΔMG
G
DAPI
PL Angpt1 Tie2
Merge
Fkbp5fl/fl
Fkbp5ΔMG
10μm
H
PLA signal area/cell number (fold change)
***
OGD/R Fkbp5fl/fl
OGD/R Fkbp5ΔMG

Fig S18, Validation of ligand-receptor pairs between microglia and endothelial cells by proximity ligation assay (PLA).

(A-B) PLA representative images (A) and quantitative data (B) showing the Wnt5a-Fzd4 interactions in *Fkbp5*$^{fl/fl}$ mice and *Fkbp5*$^{\Delta MG}$ mice at 24 hours post-tMCAO. n = 7 for each group.

(C-D) PLA representative images (C) and quantitative data (D) showing the Angpt1-Tie2 interactions in *Fkbp5*$^{fl/fl}$ mice and *Fkbp5*$^{\Delta MG}$ mice at 24 hours post-tMCAO. n = 7 for each group.

(E-F) PLA representative pictures (E) and quantitative data (F) of Wnt5a-Fzd4 interactions in co-culture of endothelial cells and microglia isolated from Fkbp5$^{fl/fl}$ mice or Fkbp5$^{\Delta MG}$ mice after OGD/R injury. n = 6 for each group.

(G-H) PLA representative pictures (G) and quantitative data (H) of Angpt1-Tie2 interactions in co-culture of endothelial cells and microglia isolated from Fkbp5$^{fl/fl}$ mice or Fkbp5$^{\Delta MG}$ mice after OGD/R injury. n = 6 for each group.

Data are presented as mean ± SD. one way ANOVA; * $P < 0.05$; ** $P < 0.01$; **** $P < 0.0001$. Fkbp5$^{fl/fl}$, *Fkbp5*$^{flox/flox}$ mice without Fkbp5 conditional deletion in microglia; Fkbp5$^{\Delta MG}$, *Fkbp5*$^{flox/flox}$ and *Cx3cr1*^{Cre} mice with Fkbp5 conditional deletion in microglia;

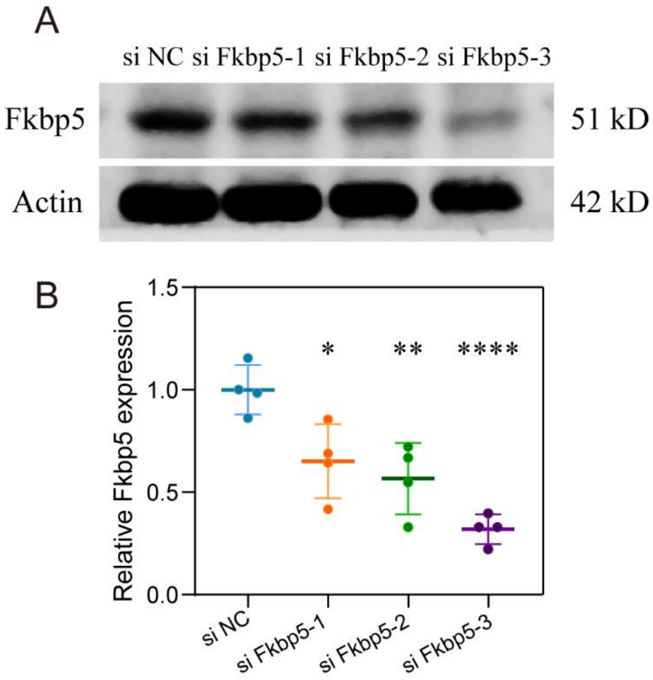

Figure S19. Identification of Fkbp5 knockdown efficacy by siRNA interfere in BV2 cells.

(A-B) Representative WB images (A) and quantitative data (B) of Fkbp5 protein expression in BV2 cells after incubating with NT siRNA, siFkbp5-1, siFkbp5-2, or siFkbp5-3 siRNAs for 24 hours. n = 4 for each administration. Data are presented as mean ± SD. one way ANOVA; * $P < 0.05$; ** $P < 0.01$; **** $P < 0.0001$.

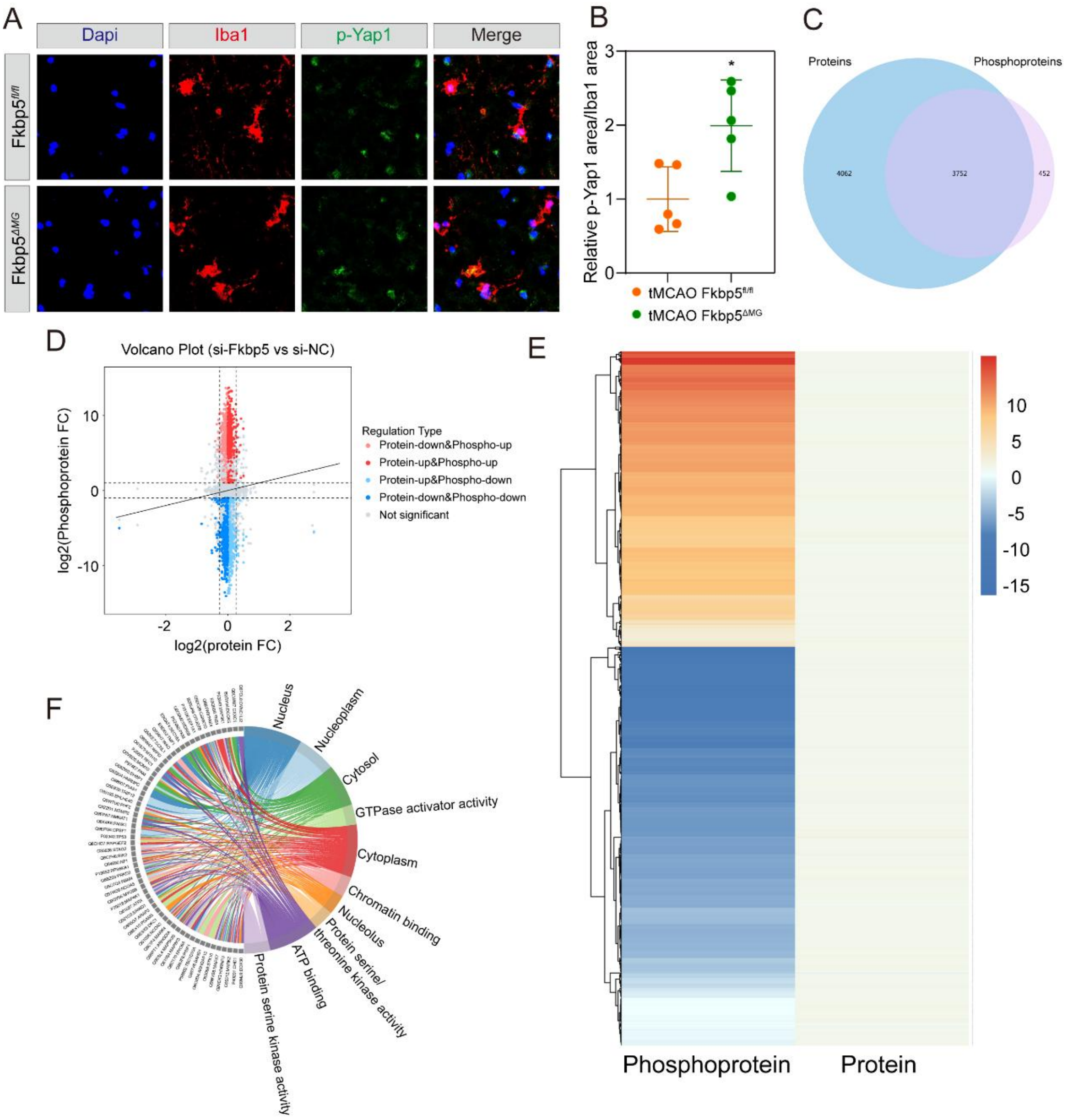

Fig S20. Quantitative phosphoproteomics and quantitative proteomics analysis of BV2 cells with or without Fkbp5 knockdown

(A) Representative micrograph of immunostaining of Iba1 and p-Yap1 in peri-infarct region of tMCAO Fkbp5$^{\Delta MG}$ mice and tMCAO Fkbp5$^{fl/fl}$ mice

(B) Quantitative data of immunostaining of Iba1 and p-Yap1 showing the p-Yap1 expression on microglial cells in peri-infarct region of tMCAO Fkbp5$^{\Delta MG}$ mice (n = 5) and tMCAO Fkbp5$^{fl/fl}$ mice (n = 5).

(C)Venn diagram illustrating the combined protein quantity from quantitative phosphoproteomics and quantitative proteomics

(D) Double volcano plot showing differentially expressed proteins in quantitative phosphoproteomics and quantitative proteomics

(E) Heatmap displaying differential proteins in quantitative phosphoproteomics and quantitative proteomics

(F) The chord diagram displays the GO enrichment analysis of differentially expressed proteins between quantitative phosphoproteomics and quantitative proteomics

Data are presented as mean ± SD. unpaired *t*-test; * $P < 0.05$; ** $P < 0.01$; *** $P < 0.001$; **** *P*

< 0.0001; DAPI, 4′,6-diamidino-2-phenylindole.

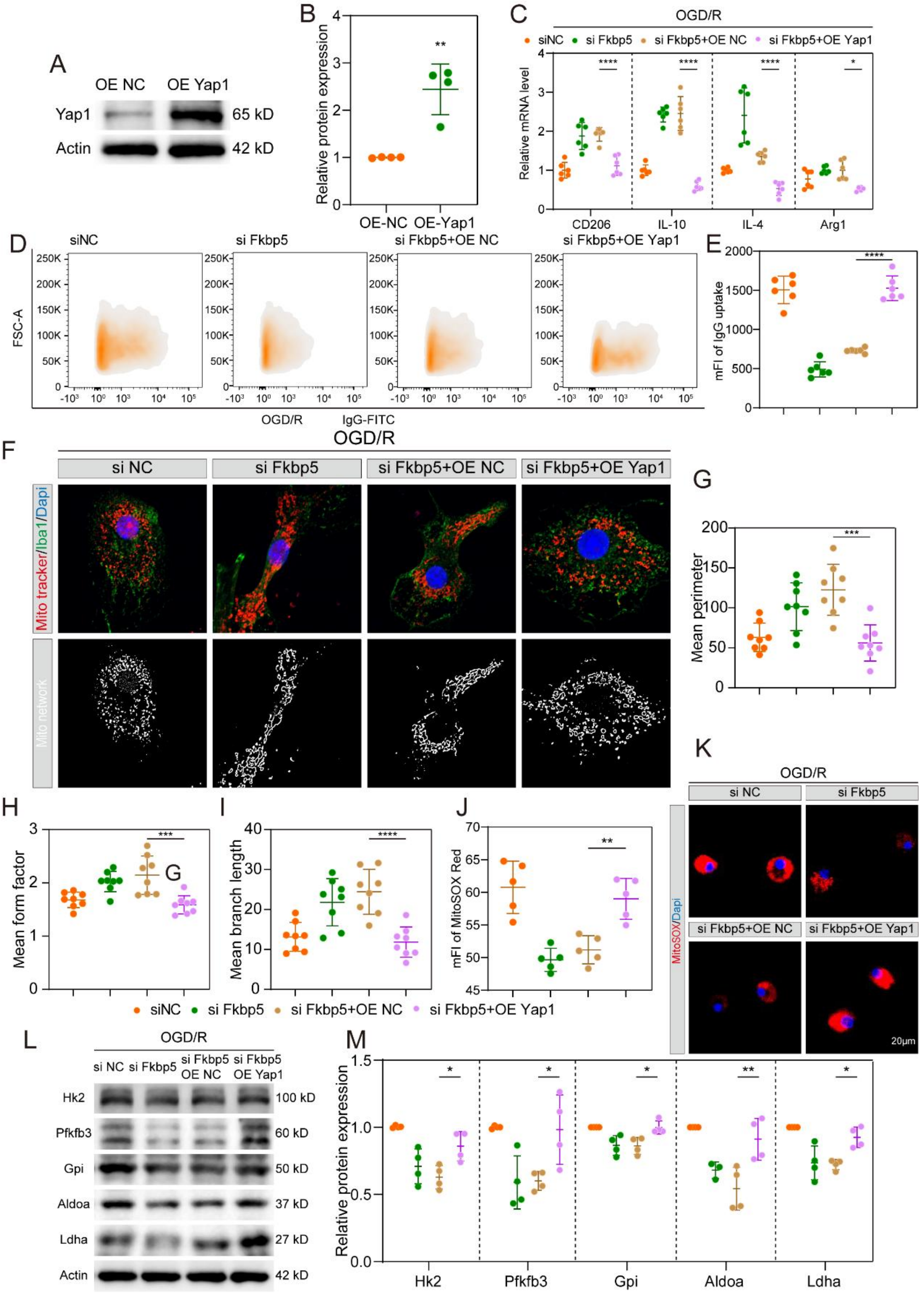

Figure S21. Yap1 overexpression counteracts the inhibitory effect of Fkbp5 knockdown on microglia.

(A-B) Immunoblots to verify the overexpression Yap1 efficacy in BV2 cells (n = 4 for each group). (C) qPCR data measuring M2 markers genes expression in BV2 cells after receiving Fkbp5

knockdown interfere alone or with Yap1 overexpression treatment under OGR/R condition. n = 6 per group.

(D) Representative flow plots of IgG-latex uptake in BV2 cells after receiving Fkbp5 knockdown interfere alone or with Yap1 overexpression treatment under OGR/R condition. n = 6 per group.

(E) Quantitative data showing the mFI of IgG-latex uptake in BV2 cells after receiving Fkbp5 knockdown interfere alone or with Yap1 overexpression treatment under OGR/R condition. n = 6 per group.

(F) Representative micrographs of mitochondrial morphology of primary microglial cells receiving Fkbp5 knockdown interfere alone or with Yap1 overexpression treatment under OGR/R condition. n = 8 per group.

(G-I) Quantitative data measuring the mitochondrial morphology of primary microglial cells, including mean perimeter (E), mean form factor (F), and mean branch length (G), receiving Fkbp5 knockdown interfere alone or with Yap1 overexpression treatment under OGR/R condition. n = 8 per group.

(J) Quantitative data showing the mFI of mitoSOX of BV2 cells receiving Fkbp5 knockdown interfere alone or with Yap1 overexpression treatment under OGR/R condition. n = 5 per group.

(K) Representative images of mitoSOX of BV2 cells receiving Fkbp5 knockdown interfere alone or with Yap1 overexpression treatment under OGR/R condition. n = 5 per group.

(L) Representative immunoblots of different expression of glycolysis enzyme of BV2 cells receiving Fkbp5 knockdown interfere alone or with Yap1 overexpression treatment under OGR/R condition. n = 4 per group.

(M) Quantitative data measuring the different expression of glycolysis enzyme of BV2 cells receiving Fkbp5 knockdown interfere alone or with Yap1 overexpression treatment under OGR/R condition. n = 4 per group.

Data are presented as mean ± SD. unpaired *t*-test; one way ANOVA; * $P < 0.05$; ** $P < 0.01$; *** $P < 0.001$; **** $P < 0.0001$. OGD/R, Oxygen Glucose Deprivation/Reperfusion; DAPI, 4′,6-diamidino-2-phenylindole.

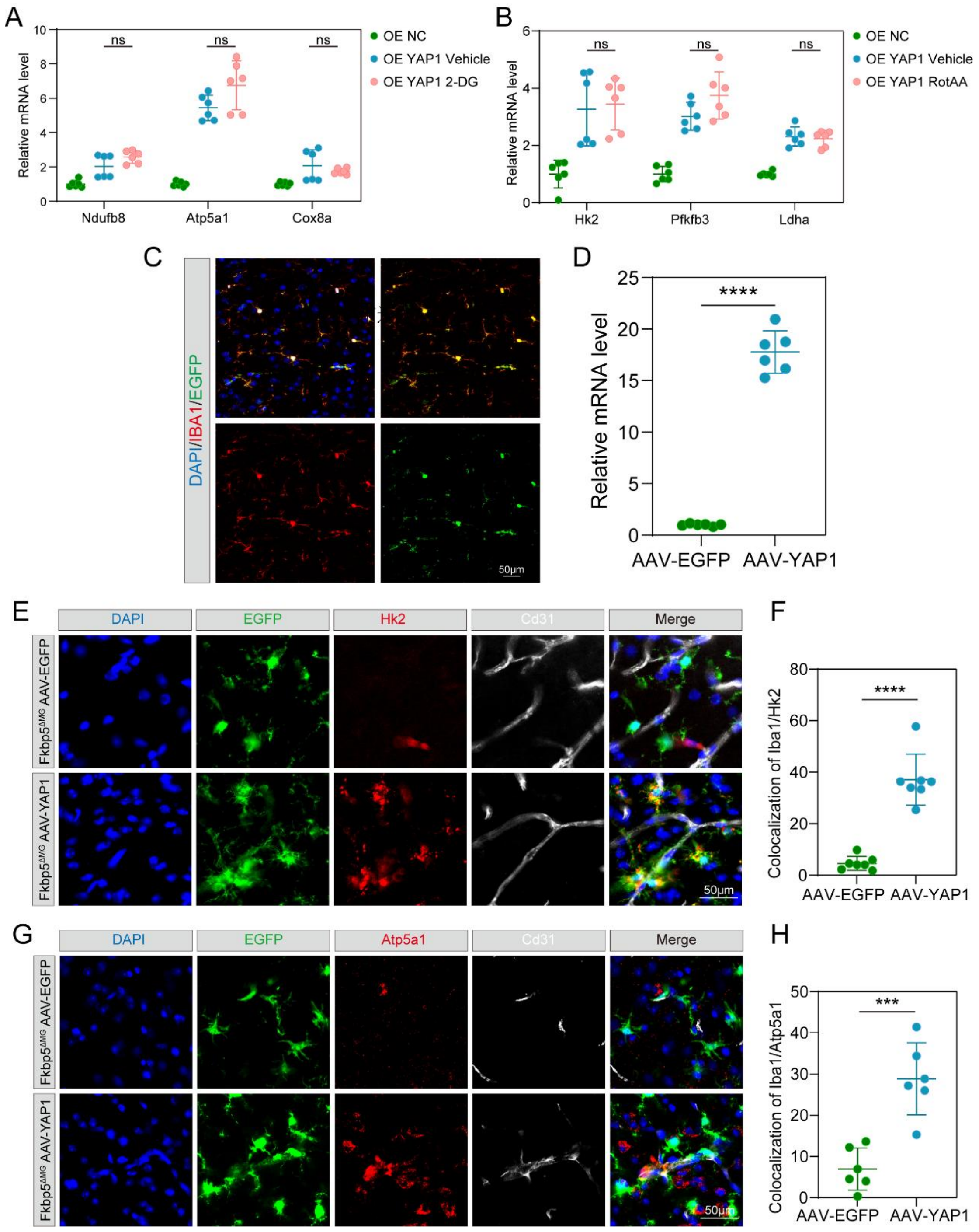

Fig S22. Elevated glycolysis and OXPHOS in stroke-VAM is co-activated by Yap1.

(A) qPCR identifying the gene expression of OXPHOS enzymes (Ndufb8, Atp5a1, and Cox8a) in BV2 cells treated with control plasmid, Yap1 overexpression plasmid and vehicle, Yap1 overexpression plasmid and glycolysis inhibitor (2-DG). n = 6 for each group.

(B) qPCR identifying the gene expression of glycolytic enzymes (Hk2, Pfkfb3, and Ldha) in BV2 cells treated with control plasmid, Yap1 overexpression plasmid and vehicle, Yap1 overexpression plasmid and RotAA. n = 6 for each group.

(C) Representative micrographs of EGFP and Iba1 in cerebral cortex

(D) qPCR identifying the Yap1 mRNA level in cerebral cortex from control AAV injected mice and

Yap1 overexpression AAV injected mice. n = 6 for each group.

(E-F) Representative micrograph (E) and quantitative data (F) of EGFP and Hk2 immunofluorescence in ipsilateral cortex from control AAV injected mice and Yap1 overexpression AAV injected mice.). n = 7 for each group.

(G-H) Representative micrograph (G) and quantitative data (H) of EGFP and Atp5a1 immunofluorescence in ipsilateral cortex from control AAV injected mice and Yap1 overexpression AAV injected mice.). n = 6 for each group.

Data are presented as mean ± SD. unpaired *t*-test; one way ANOVA; * $P < 0.05$; ** $P < 0.01$; *** $P < 0.001$; **** $P < 0.0001$. AAV, Adeno-Associated Virus; DAPI, 4′,6-diamidino-2-phenylindole; OE, overexpression.

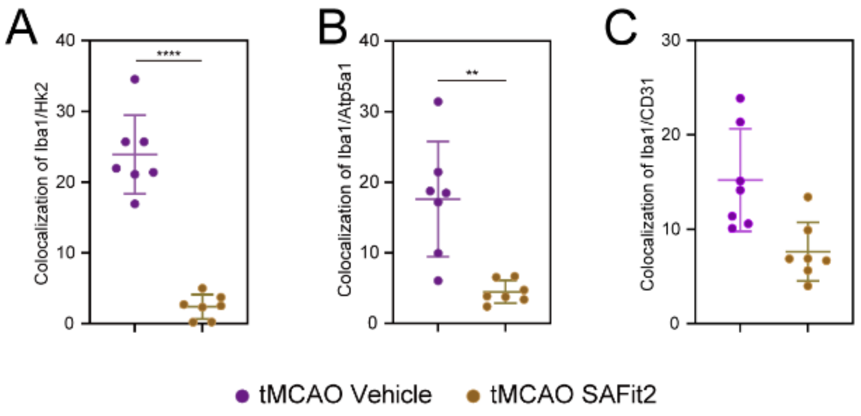

Fig S23. Quantitative data of figure 8R-8T

(A) Quantitative data of figure 8R measuring the expression of Hk2 in stroke-VAM from mice administrated with vehicle (n = 7) or SAFit2 (n = 7) after ischemic stroke.

(B) Quantitative data of figure 8S measuring the expression of Atp5a1 in stroke-VAM from mice administrated with vehicle (n = 7) or SAFit2 (n = 7) after ischemic stroke.

© Quantitative data of the number of stroke-VAM from mice administrated with vehicle (n = 7) or SAFit2 (n = 7) after ischemic stroke.

Data are presented as mean ± SD. Unpaired *t*-test; one way ANOVA; * $P < 0.05$; ** $P < 0.01$; *** $P < 0.001$; **** $P < 0.0001$. tMCAO, transient Middle Cerebral Artery Occlusion;

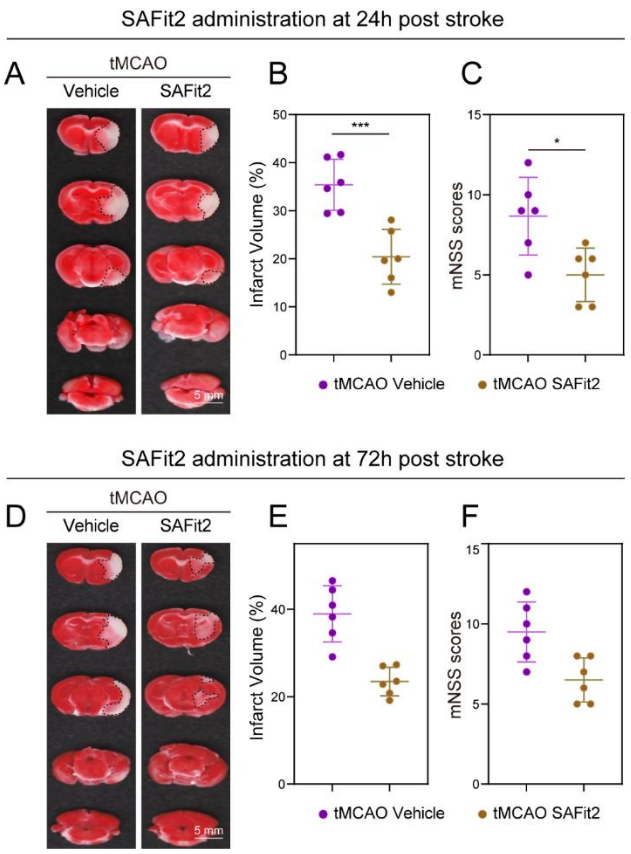

Fig S24. The therapeutic time window for SAFit2 can be extended up to 72 hours in murine tMCAO model.

(A-B) Representative pictures (A) and quantitative data (B) of infarct volume in tMCAO mice receiving vehicle or SAFit2 at 24 hours after tMCAO measured by TTC staining. n = 6 for each group.

(C) Neurological function evaluation by mNSS score system in tMCAO mice receiving vehicle or SAFit2 at 24 hours post stroke. n = 6 per group.

(D-E) Representative pictures (D) and quantitative data (E) of infarct volume in tMCAO mice receiving vehicle or SAFit2 at 72 hours after tMCAO measured by TTC staining. n = 6 for each group.

(F) Neurological function evaluation by mNSS score system in tMCAO mice receiving vehicle or SAFit2 at 72 hours post stroke. n = 6 per group.

Data are presented as mean ± SD. Unpaired *t*-test; $*p < 0.05$; $**p < 0.01$; $***p < 0.001$. $****p < 0.0001$. tMCAO, transient Middle Cerebral Artery Occlusion;

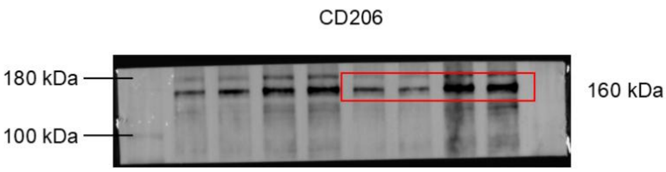

Figure S25. The complete Western blot images for CD206 in Figure 4F.

Supplemental Tables

Table S2. Baseline Characteristics of the Study Participants

Characteristic	Overall (n=184)	Good Outcome (n=92)	Poor Outcome (n=92)	p-value
Demographics				
Age (years), median (IQR)	68 (59-76)	67 (58-75)	69 (60-77)	0.45
Male, n (%)	112 (60.9%)	56 (60.9%)	56 (60.9%)	>0.99
Clinical Characteristics				
Baseline NIHSS score, median (IQR)	17 (15-19)	15 (13-17)	18 (16-21)	<0.001
BMI (kg/m²), mean ± SD	24.1 ± 3.2	23.9 ± 3.0	24.3 ± 3.4	0.38
Systolic BP (mmHg), mean ± SD	152.3 ± 18.7	150.1 ± 17.9	154.5 ± 19.3	0.11
Diastolic BP (mmHg), mean ± SD	86.5 ± 11.2	85.8 ± 10.7	87.2 ± 11.7	0.38
Medical History, n (%)				
Hypertension	138 (75.0%)	68 (73.9%)	70 (76.1%)	0.74
Diabetes mellitus	62 (33.7%)	30 (32.6%)	32 (34.8%)	0.76
Atrial fibrillation	45 (24.5%)	22 (23.9%)	23 (25.0%)	0.87
Smoking history	59 (32.1%)	30 (32.6%)	29 (31.5%)	0.87
Alcohol drinking history	48 (26.1%)	25 (27.2%)	23 (25.0%)	0.74
Laboratory Tests				
FKBP5 (ng/mL), median (IQR)	0.142 (0.095 - 0.327)	0.138 (0.091 - 0.256)	0.145 (0.098 - 0.379)	0.042
Fasting glucose (mmol/L), mean ± SD	6.8 ± 1.9	6.7 ± 1.8	6.9 ± 2.0	0.47
LDL-C (mmol/L), mean ± SD	2.7 ± 0.8	2.6 ± 0.7	2.8 ± 0.9	0.09

IQR, interquartile range; SD, standard deviation; NIHSS, National Institutes of Health Stroke Scale; BMI, body mass index; BP, blood pressure; LDL-C, low-density lipoprotein cholesterol.

Table S3: Single-Cell Analysis Reproducibility Checklist

Project	Version or Threshold	Verification location
R Software	R 4.5.1	R 4.5.1
Core Dependency Packages	Seurat, GSVA, etc.	Details in code
Code	**File S1**	Details in **File S1**
Clustering Resolution	0.4 for all cells clustering;<br>0.6 for microglia clustering	Details in code
Differential Gene Expression Threshold	adj. P<0.05, $\|\log_2FC\|$>0.5	Details in code
ssGSEA Parameters	tau=0.25, method='ssgsea'	Details in code
ssGSEA Geneset	Downloaded by Gene Set Enrichment Analysis database (https://www.gsea-msigdb.org/gsea/index.jsp)	Details in **Table S1**.
Raw Data	GEO: GSE174574	Details in Methods
Intermediate Data	Raw data after clustering and annotation	Provided in **File S2**

Table S4 Antibodies used in this study

**Antibodies used in the study**			
**Primary antibodies used for Immunofluorescence**			
**Reagent**	**Source**	**Identifier**	**Species**
Iba1	Abcam	ab5076	Goat
Iba1	Abcam	ab318302	Chicken
Hk2	Santa Cruz	sc-130358	Mouse
Atp5a1	Santa Cruz	sc-136178	Mouse
CD31	R&D Systems	AF3628	Goat
Fkbp5	Santa Cruz	sc-271547	Mouse
Neun	Proteintech	26975-1-AP	Rabbit
Gfap	Abcam	Ab4674	Chicken
Fibrinogen	Abcam	ab118533	Sheep
Lectin	Vector laboratories	DL-1177	594-conjugated
Cd68	Abcam	ab201340	Mouse
β catenin	Proteintech	17565-1-AP	Rabbit
Tie2	Proteintech	19157-1-AP	Rabbit
p-Yap1	CellSignaling Technology	13008	Rabbit

Yap1	Proteintech	13584-1-AP	Rabbit
**Secondary antibodies used for Immunofluorescence**			
**Target Species**	**Source**	**Identifier**	**conjugates**
anti-Goat IgG (H+L)	Invitrogen	A-32814	Alexa Fluor™ Plus 488
anti-Mouse IgG (H+L)	Jackson	715-585-150	Alexa Fluor® 594
anti-Goat IgG (H+L)	Invitrogen	A-21447	Alexa Fluor™ 647
anti-Chicken IgY (IgG) (H+L)	Jackson	703-095-155	FITC
anti-Chicken IgY (IgG) (H+L)	Jackson	103-585-155	Alexa Fluor® 594
anti-Mouse IgG (H+L)	Invitrogen	A-11001	Alexa Fluor™ 488
anti-Goat IgG (H+L)	Invitrogen	A-11058	Alexa Fluor™ 594
anti-Rabbit IgG (H+L)	Invitrogen	A-11012	Alexa Fluor™ 594
anti-Sheep IgG (H+L)	Invitrogen	A-11015	Alexa Fluor™ 488
anti-Rabbit IgG (H+L)	Invitrogen	A-11008	Alexa Fluor™ 488
**Antibodies used for Western Blot**			
**Reagent**	**Source**	**Identifier**	**Species**
Fkbp5	Proteintech	14155-1-AP	Rabbit
ZO-1	Proteintech	21773-1-AP	Rabbit
VE cadherin	R&D Systems	AF1002	Goat
Occludin	Proteintech	27260-1-AP	Rabbit
Claudin5	Abclonal	A10207	Rabbit
Actin	Abclonal	AC006	Rabbit
CD206	Proteintech	18704-1-AP	Rabbit
IL-10	Abclonal	A2171	Rabbit
IL-4	Abclonal	A14660	Rabbit
Hk2	Proteintech	22029-1-AP	Rabbit
Pfkfb3	Proteintech	13763-1-AP	Rabbit
Gpi	Proteintech	15171-1-AP	Rabbit
Aldoa	Proteintech	11217-1-AP	Rabbit
Ldha	Proteintech	19987-1-AP	Rabbit
Yap1	Abclonal	A1002	Rabbit
p-Yap1	Abclonal	AP0489	Rabbit
Lamin B1	Proteintech	12987-1-AP	Rabbit
p-Lats1	Abclonal	AP0904	Rabbit

Lats1	Abclonal	A17992	Rabbit
p-Mst1	Abclonal	AP0906	Rabbit
Mst1	Abclonal	A8043	Rabbit
β tubulin	Abclonal	A12289	Rabbit
**Antibodies used for Flow Cytometry**			
**Target (mouse)**	**Source**	**Identifier**	**conjugates**
Dead cells	Biolegend	420403	7-AAD
CD11b	Biolegend	101205	FITC
CD45	Biolegend	157617	APC/Cyanine7
Ly6g	Biolegend	127618	PE/Cyanine7
CD206	Biolegend	141705	PE
IL-10	Biolegend	505009	APC

Table S5 Primers used in this study

Gene	Forward sequence	Reward sequence
Fkbp5	TGAGGGCACCAGTAACAATGG	CAACATCCCTTTGTAGTGGACAT
Aif1	ATCAACAAGCAATTCCTCGATGA	CAGCATTCGCTTCAAGGACATA
Rbfox3	ATCGTAGAGGGACGGAAAATTGA	GTTCCCAGGCTTCTTATTGGTC
Pecam1	ACGCTGGTGCTCTATGCAAG	TCAGTTGCTGCCCATTCATCA
S100β	TGGTTGCCCTCATTGATGTCT	CCCATCCCCATCTTCGTCC
Arg1	CTCCAAGCCAAAGTCCTTAGAG	AGGAGCTGTCATTAGGGACATC
Cd206	CTCTGTTCAGCTATTGGACGC	CGGAATTTCTGGGATTCAGCTTC
Ym1	CAGGTCTGGCAATTCTTCTGAA	GTCTTGCTCATGTGTGTAAGTGA
Il10	GCTATGCTGCCTGCTCTTACT	CCTGCTGATCCTCATGCCA
Tgfb	CTCCCGTGGCTTCTAGTGC	GCCTTAGTTTGGACAGGATCTG
Cd163	ATGGGTGGACACAGAATGGTT	CAGGAGCGTTAGTGACAGCAG
Hk2	TGATCGCCTGCTTATTCACGG	AACCGCCTAGAAATCTCCAGA
Pfkfb3	CCCAGAGCCGGGTACAGAA	GGGGAGTTGGTCAGCTTCG
Aldoa	CGTGTGAATCCCTGCATTGG	CAGCCCCTGGGTAGTTGTC
Eno1	TGCGTCCACTGGCATCTAC	CAGAGCAGGCGCAATAGTTTTA
Pkm2	GCCGCCTGGACATTGACTC	CCATGAGAGAAATTCAGCCGAG
Ldha	TGTCTCCAGCAAAGACTACTGT	GACTGTACTTGACAATGTTGGGA
Sdha	GGAACACTCCAAAAACAGACCT	CCACCACTGGGTATTGAGTAGAA
Ndufb8	TGTTGCCGGGGTCATATCCTA	AGCATCGGGTAGTCGCCATA
Cox8a	GGTTCGGCCATCTTGACTCC	GCCCAATGGTGATATCCAGGACC
Cycs	CCAAATCTCCACGGTCTGTTC	ATCAGGGTATCCTCTCCCCAG
Atp5f1a	TCTCCATGCCTCTAACACTCG	CCAGGTCAACAGACGTGTCAG
Yap1	ACCCTCGTTTTGCCATGAAC	TGTGCTGGGATTGATATTCCGTA
Cyr61	CTCGCCTTAGTCGTCACCC	CGCCGAAGTTGCATTCCAG
Ctgf	CAGCATGGACGTTCGTCTG	AACCACGGTTTGGTCCTTGG
Ankrd1	AGTAGAGGAACTGGTCACTGG	TGTTTCTCGCTTTTCCACTGTT

Table S6: Checklist of the ARRIVE 2.0 guidelines (for animal experiments)

Section/Topic	ARRIVE 2.0 Guideline Item	Reported (YES/NA/NO)	Location in Manuscript
Study design	1a. The groups being compared, including control groups. If no control group, explain why.	YES	Details in the figure legends Groups: Sham, tMCAO; tMCAO Fkbp5$^{fl/fl}$, tMCAO Fkbp5$^{\Delta MG}$; tMCAO+Vehicle, tMCAO+SAFit2.
	1b. The experimental unit (e.g., a single animal, a litter, a cage).	YES	The experimental unit is a single animal. Sample size (n) per group is clearly stated in all figure legends.
Sample size	2a. Specify the total number of animals used in each experiment, and the number of animals in each experimental group.	YES	Figure legends explicitly state the n per group for each experiment.
	2b. Explain how the sample size was decided. Include details of any sample size calculation used.	YES	Details in the Method section Statistical analysis
Inclusion and exclusion criteria	3a. Describe any criteria used for including and excluding animals (or experimental units) during the experiment, and data points during the analysis. Specify if these criteria were established a priori. If no criteria were set, state this explicitly.	Yes	Details in Method section-tMCAO "Inclusion: Successful occlusion Exclusion: dead during tMCAO surgery"
	3b. For each experimental group, report any animals, experimental units or data points not included in the analysis and explain why. If there were no exclusions, state so.	YES	NO Exclusions
	3c. For each analysis, report the exact value of n in each experimental group.	YES	Figure legends explicitly state the n per group for each experiment.
Randomisation	4a. State whether randomisation was used to allocate experimental units to control and treatment groups. If done, provide the method used to generate the randomisation sequence.	YES	Details in the Method section Statistical analysis "All experiments applied unbiased design, randomized allocation..." Grouping was implemented using a computer-generated random number table.
	4b. Describe the strategy used to minimise potential confounders such as the order of treatments and measurements, or animal/cage location. If confounders were not controlled, state this explicitly.	YES	Details in the Method section Randomization, Standardization of Environment and Procedures, and Blinding
Blinding	5. Describe who was aware of the group allocation at the different stages of the experiment (during the allocation, the conduct of the experiment, the outcome assessment, and the data analysis).	YES	The surgeon was blinded to the group allocation information. Pathological analysis and histological staining assessments were independently performed by two professional researchers who were also blinded to the grouping details.
Outcome measures	6a. Clearly define all outcome measures assessed (e.g. cell death, molecular markers, or behavioural changes).	YES	All outcome measures are clearly defined in the Materials and Methods

			section.
	6b. For hypothesis-testing studies, specify the primary outcome measure, i.e. the outcome measure that was used to determine the sample size.	YES	Details in the Method section Statistical analysis
Statistical methods	7a. Provide details of the statistical methods used for each analysis, including software used.	YES	Details in the Method section Statistical analysis and figure legends
	7b. Describe any methods used to assess whether the data met the assumptions of the statistical approach, and what was done if the assumptions were not met.	YES	Details in the Method section Statistical analysis and figure legends
Experimental animals	8a. Provide species-appropriate details of the animals used, including species, strain and substrain, sex, age or developmental stage, and, if relevant, weight.	YES	Details in the Method Section-Animals C57BL/6J mice (8-12 weeks old, 20-25g) *Fkbp5*$^{flox/flox}$ mice (The Cyagen Biosciences Inc.) and *Cx3cr1*^{Cre} mice (The Jackson Laboratory)
	8b. Provide further relevant information on the provenance of animals, health/immune status, genetic modification status, genotype, and any previous procedures.	YES	Details in the Method Section-Animals C57BL/6J mice (Beijing Vital River) *kbp5*$^{flox/flox}$ mice (The Cyagen Biosciences Inc.) and *Cx3cr1*^{Cre} mice (The Jackson Laboratory)
Experimental procedures	9. For each experimental group, including controls, describe the procedures in enough detail to allow others to replicate them, including: a. What was done, how it was done and what was used. b. When and how often. c. Where (including detail of any acclimatisation periods). d. Why (provide rationale for procedures)	YES	Detailed protocols for tMCAO, behavior tests, tissue collection, etc., are in Materials and Methods.
Results	10. For each experiment conducted, including independent replications, report: a. Summary/descriptive statistics for each experimental group, with a measure of variability where applicable (e.g. mean and SD, or median and range). b. If applicable, the effect size with a confidence interval.	YES	Results are comprehensively reported for all outcome measures in the Results section and figures.

Table S7: Molecular Assay: Minimum Information for Publication of Quantitative Real-Time PCR Experiments (MIQE) Checklist

Section/Topic	**MIQE Guideline Item**	**Description or Location in Manuscript**
Experimental design	Definition of experimental and control groups	Clearly defined for each experiment (e.g., Sham vs. tMCAO time course; Fkbp5$^{fl/fl}$ vs. Fkbp5ΔMG)
	Number within each group	Sample size (*n*) per group is clearly stated in all figure legends
Sample	Description	The information for each sample and the grouping details are described in the Methods and Results sections
	Microdissection or macrodissection	Described in results and figure legends section “ipsilateral cortex”
	Processing procedure	Described in the Methods section- qRT-PCR

	If frozen, how and how quickly?	Samples were flash-frozen in liquid nitrogen within 10 minutes after obtained
	If fixed, with what and how quickly?	Not applicable
	Sample storage conditions and duration (especially for FFPE samples)	Stored in liquid nitrogen prior to RNA extraction within one week
Nucleic acid extraction	Procedure and/or instrumentation	For details, please refer to the manufacturer's instructions provided with the Total RNA extraction reagent (Cat#R401-01, Vazyme)
	Name of kit and details of any modifications	
	Details of DNase or RNase treatment	
	Contamination assessment (DNA or RNA)	
	Instrument and method	Eppendorf 5810R
	Nucleic acid quantification	A260/A280, A260/A230
	RNA integrity: method/instrument	Thermo NanoDrop
	RIN/RQI or C_q of 3′ and 5′ transcripts	RQI (8.0-8.8)
	Inhibition testing (C_q dilutions, spike, or other)	The ratio of Cq values for GAPDH and ACTIN remained consistent across different samples
Reverse transcription	Complete reaction conditions	For details, please refer to the manufacturer's instructions provided with the ABScript III RT Master Mix for qPCR (Cat. #RK20429, ABclonal, China).
	Amount of RNA and reaction volume	
	Priming oligonucleotide (if using GSP) and concentration	
	Reverse transcriptase and concentration	
	Temperature and time	
	Storage conditions of cDNA	
qPCR target information	Gene symbol; Sequence accession number; Amplicon length; In silico specificity screen (BLAST, and so on)	Details in **Table S4**
	What splice variants are targeted?	Not applicable
qPCR oligonucleotides	Primer sequences	Details in **Table S4**
qPCR protocol	Complete reaction conditions	For details, please refer to the manufacturer's instructions provided with the 2×Universal SYBR Green Fast qPCR Mix (RK21203, ABclonal, China)
	Reaction volume and amount of cDNA/DNA	
	Primer, (probe), Mg^{2+}, and dNTP concentrations	
	Polymerase identity and concentration	
	Buffer/kit identity and manufacturer	
	Additives (SYBR Green I, DMSO, and so forth)	
	Complete thermocycling parameters	
	Manufacturer of qPCR instrument	CFX Real-Time PCR System (Biorad)
qPCR validation	Specificity (gel, sequence, melt, or digest)	Single sharp melting peak
	For SYBR Green I, C_q of the NTC	**>38**
	Calibration curves with slope and *y* intercept	3.1~-3.6
	PCR efficiency calculated from slope	90%~110%
	r^2 of calibration curve	≥0.995
	Linear dynamic range	10^1~10^6 copy /μL
	C_q variation at LOD	≤5%

	Evidence for LOD	⩾3
Data analysis	qPCR analysis program (source, version)	Bio-Rad CFX Maestro
	Method of C_q determination	Auto Threshold (1-15 cycle)
	Outlier identification and disposition	Technical replicate Cq values: coefficient of variation (CV) >5%
	Justification of number and choice of reference genes	*Actin/Gapdh*
	Description of normalization method	Gene expression levels were calculated using the comparative ΔΔCt method, with *gapdh* and *actb* serving as internal control housekeeping genes.
	Number and stage (reverse transcription or qPCR) of technical replicates	Three technical replicates
	Repeatability (intraassay variation)	Intra-assay coefficient of variation ⩽5%
	Statistical methods for results significance	Unpaired Student's t-test, One-way or Two-way ANOVA details in Methods
	Software (source, version)	GraphPad Prism v9.0 (La Jolla, USA)
